# Novel splice variants derived from the receptor tyrosine kinase superfamily are potential therapeutics for rheumatoid arthritis

**DOI:** 10.1186/ar2447

**Published:** 2008-07-01

**Authors:** Pei Jin, Juan Zhang, Percy F Sumariwalla, Irene Ni, Brett Jorgensen, Damian Crawford, Suzanne Phillips, Marc Feldmann, H Michael Shepard, Ewa M Paleolog

**Affiliations:** 1 Receptor BioLogix, Inc., Palo Alto, CA 94303, USA; 2 Kennedy Institute of Rheumatology, Faculty of Medicine, Imperial College London, London W6 8LH, UK; 3 Gentris Corporation, Morrisville, NC 27560, USA

## Abstract

**Introduction:**

Despite the advent of biological therapies for the treatment of rheumatoid arthritis, there is a compelling need to develop alternative therapeutic targets for nonresponders to existing treatments. Soluble receptors occur naturally *in vivo*, such as the splice variant of the cell surface receptor for vascular endothelial growth factor (VEGF) – a key regulator of angiogenesis in rheumatoid arthritis. Bioinformatics analyses predict that the majority of human genes undergo alternative splicing, generating proteins – many of which may have regulatory functions. The objective of the present study was to identify alternative splice variants (ASV) from cell surface receptor genes, and to determine whether the novel proteins encoded exert therapeutic activity in an *in vivo *model of arthritis.

**Methods:**

To identify novel splice variants, we performed RT-PCR using an mRNA pool representing major human tissue types and tumors. Novel ASV were identified by alignment of each cloned sequence to its respective genomic sequence in comparison with full-length transcripts. To test whether these ASV have biologic activity, we characterized a subset of them for ligand binding, and for efficacy in an animal model of arthritis. The *in vivo *study was accomplished using adenoviruses expressing secreted ASV.

**Results:**

We cloned 60 novel human ASV from 21 genes, encoding cell surface receptors – many of which are known to be important in the regulation of angiogenesis. The ASV were characterized by exon extension, intron retention and alternative exon utilization. Efficient expression and secretion of selected ASV – corresponding to VEGF receptor type 1, VEGF receptor type 2, VEGF receptor type 3, angiopoietin receptor Tie1, Met (receptor for hepatocyte growth factor), colony-stimulating factor 1 receptor, platelet-derived growth factor receptor beta, fibroblast growth factor receptor 1, Kit, and RAGE – was demonstrated, together with binding to their cognate ligands. Importantly, ASV derived from VEGF receptor type 1 and Tie1, and to a lesser extent from VEGF receptor type 2 and fibroblast growth factor receptor 1, reduced clinical signs of arthritis *in vivo*. The reduction was paralleled by decreased joint inflammation and destruction.

**Conclusion:**

The present study shows that unique ASV derived from receptors that play key roles in angiogenesis – namely, VEGF receptor type 1 and, for the first time, Tie1 – can markedly reduce arthritis severity. More broadly, our results demonstrate that ASV are a source of novel proteins with therapeutic potential in diseases in which angiogenesis and cellular hyperplasia play a central role, such as rheumatoid arthritis.

## Introduction

Rheumatoid arthritis (RA) has a prevalence of about 1% in most parts of the world. While targeting TNFα using biological inhibitors has been an undoubted success, efficacy does not usually approach remission. Moreover, increasing usage of anti-TNFα biological agents in RA is associated with an augmented risk of infections, including tuberculosis [1-5]. As a consequence, initiatives to develop alternative targets in RA are desirable, especially for use in combination with TNFα inhibitors.

Cell surface receptors such as receptor tyrosine kinases (RTKs) mediate ligand-induced signal transduction from the extracellular to the intracellular environment. Dysregulation of RTK signaling is implicated in the pathogenesis of many human diseases, including cancer and autoimmune diseases [6,7]. The discovery that soluble forms of receptors can abrogate receptor–ligand interaction has fueled substantial interest in their potential application as biotherapeutics. Etanercept, a molecularly engineered fusion protein composed of the extracellular domain of TNF receptor type II, is an example of a clinically effective soluble receptor-based therapeutic, with potent activity in RA [8].

Soluble receptors are known to occur naturally *in vivo *[9]. Two major mechanisms involved in the formation of naturally occurring soluble receptors are proteolytic cleavage of membrane receptors and alternative pre-mRNA splicing. The latter is a process in which multiple proteins are created from a single pre-mRNA [10-13]. Bioinformatics analyses predict that the majority of human genes undergo alternative splicing, suggesting that alternative splicing is a significant component in generating diversity of function in the human genome [11]. The protein products of alternative splicing may serve as homeostatic regulators in physiology and disease [14-16]. This is illustrated by the splice variant of vascular endothelial growth factor receptor (VEGFR) type 1 (sVEGFR1 or sFlt-1). Vascular endothelial growth factor (VEGF) plays a pivotal role in regulating angiogenesis, and binds sFlt-1 *in vivo*. Suppression of endogenous sFlt-1 was found to abolish corneal avascularity in mice [17]. Conversely, sFlt-1 has been shown to modulate disease in other *in vivo *models, including animal models of RA [18-22].

To determine the frequency of functional soluble splice forms of cell surface receptors, we have developed a high-throughput method for gene scanning, cloning, and characterization that identified functional alternative splice variants (ASV). The present work describes the RT-PCR selection and molecular cloning of 60 novel soluble receptors as splice variants of 21 RTKs and other cell surface receptor genes, including VEGF and TNF receptors. These cell surface receptor-derived ASV differ from transmembrane proteins, or shed receptors, by the deletion or addition of unique amino acids as a result of alternative splicing events, including exon extensions and deletions. The novel ASV that we identified included splice variants of receptors for VEGF (VEGFR1, VEGFR2 and VEGFR3) and for angiopoietin-1 (Ang-1) receptor Tie1 (tyrosine kinase with immunoglobulin and epidermal growth factor homology domains 1), as well as for platelet-derived growth factor receptor beta (PDGFRβ) and fibroblast growth factor receptors (FGFRs).

We selected 10 ASV for further analysis, chosen on the basis of their potential effects on angiogenesis, which represent an attractive target for therapy in RA [23-28]. We confirmed that ASV derived from cell surface receptors retained their ligand binding ability and were transcribed in human normal and malignant tissues. Furthermore, using adenoviruses expressing secreted ASV, we demonstrated that these ASV exhibit differential effects in a murine model of RA – namely, collagen-induced arthritis (CIA), which is in widespread use as a tool for developing new therapeutics. Work in the acute CIA model formed the basis for the widespread clinical use of TNFα inhibitors for treatment of RA [29-32]. Moreover, we and other workers have shown that inhibition of angiogenesis ameliorates disease [18,20,33-38]. We observed that ASV corresponding to VEGFR1, and to a lesser extent VEGFR2, reduced arthritis severity, in agreement with our earlier findings using sFlt-1 [18,20]. We also observed for the first time that ASV corresponding to Tie1 significantly reduced arthritis severity and joint destruction. While expression of Ang-1 [39,40] and of Tie receptors [41-43] has been reported in RA, this is the first demonstration that Tie1 is effective in an *in vivo *model of arthritis. We also observed a modest effect of FGFR1 ASV in acute CIA.

These data establish that ASV derived from receptors that play key roles in angiogenesis – VEGFR1 and, for the first time, Tie1 – can reduce arthritis severity. More broadly, ASV are a source of novel proteins with therapeutic potential in diseases in which angiogenesis and cellular hyperplasia play a central role, such as RA.

## Materials and methods

### Materials

Human umbilical vein endothelial cells (HUVEC) and endothelial cell medium-2 were obtained from Cambrex (East Rutherford, NJ, USA). Tie1-751 was ^125^I-custom-labeled by GE-Amersham (Piscataway, NJ, USA). Anti-human Tie1 (C18) and Tie2 (C-20) rabbit polyclonal antibodies specific to the C-terminal receptor domains were obtained from Santa Cruz Biotechnology (Santa Cruz, CA, USA). Mouse penta-His antibody was obtained from Qiagen (Valencia, CA, USA). Anti-Myc mouse monoclonal antibody (9E10) was obtained from Roche Diagnostics (Indianapolis, IN, USA). Antibodies detecting extracellular domains of soluble receptors, human VEGFR1/Fc and VEGFR3/Fc chimeras, human VEGF-C, VEGF-D and VEGF_165_, and anti-human VEGF-D polyclonal antibody were obtained from R&D Systems (Minneapolis, MN, USA).

### RT-PCR cloning of novel alternative splice variants and generation of alternative splice variant adenoviruses

mRNAs that represent major human tissue types from healthy or diseased tissues and from cell lines were purchased from Clontech (Mountain View, CA, USA) and from Strategene (La Jolla, CA, USA), and were pooled. Synthesis of the first-strand cDNA was performed using STRATASCRIPT reverse transcriptase (Stratagene) following the manufacturer's instructions. For PCR amplification, gene-specific PCR primers were selected. The forward primers flanked the start codon. The reverse primers were selected from the transmembrane region of the receptors. PCR conditions were 35 cycles of 95°C for 45 seconds, 60°C for 50 seconds, and 72°C for 5 minutes. The reaction was terminated with an elongation step of 72°C for 10 minutes.

PCR products were electrophoresed on 1% agarose gel, and were stained with Gelstar (BioWhittaker, Walkersville, MD, USA). The DNA bands were extracted with the QiaQuick^® ^gel extraction kit (Qiagen), ligated into the pDrive UA-cloning vector (Qiagen), and transformed into *Escherichia coli*. Recombinant plasmids were selected on bacterial agar plates containing 100 μg/ml carbenicillin. For each transfection, 200 to 1,000 colonies were randomly picked and their cDNA insert sizes were determined by PCR with M13 forward vector and reverse vector primers. Representative clones from PCR products with distinguishable molecular masses as visualized by fluorescence imaging (Alpha Innotech, San Leandro, CA, USA) were completely sequenced.

For the bioinformatics analyses, computational analysis of alternative splicing was performed by alignment of each cDNA sequence to its respective genomic sequence using SIM4 (software for analysis of splice variants; Pennsylvania State University, Centre County, Pennsylvania, USA). Only transcripts with canonical (for example, GT–AG) donor–acceptor splicing sites were considered for further analysis.

The replication-deficient adenoviral expression system ViraPower was used for subcloning and expression of the ASV proteins following the manufacturer's instructions (Invitrogen, Carlsbad, CA, USA). Recombinant ASV-expressing adenoviruses were produced and amplified in HEK293A cells (Invitrogen), purified through a double-cesium chloride centrifugation procedure, and titrated by measuring the plaque-forming units or the infectious particle units in HEK293 cells. The Adv-Fc control virus, expressing a murine IgG_2a _Fc fragment, has been previously described [44]. Adv-LacZ virus was purchased from Welgen (Worcester, MA, USA).

### Alternative splice variant mRNA expression

Expression of ASV mRNA was analyzed using RT-PCR and quantitative RT-PCR. Human normal RNA and tumor RNA (Total RNA Master Panel II) was purchased from Clontech and was DNase treated. First-strand cDNA was synthesized using the ABI High Fidelity Kit (Applied Biosystems, Foster City, CA, USA). For PCR amplification, the primers were designed using Oligo 6 (Molecular Biology Insights, Inc., Cascade, CO, USA). The condition for PCR amplification of FGFR4 and FGFR4-ASV was 30 cycles of 95°C for 45 seconds, 60°C for 50 seconds, and 72°C for 1 minute. The reaction was terminated with an elongation step of 72°C for 10 minutes.

For quantitative RT-PCR, gene-specific primers and probes were designed and assayed for specificity and efficiency using a human universal RNA sample. Quantitative RT-PCR was performed using an ABI 7900 HT sequence detection system (Applied Biosystems, Foster City, CA, USA) and TaqMan^® ^chemistries. cDNA was amplified in triplicate wells for both the normal and variant gene on the same plate. Cycle threshold values were determined and the average cycle threshold values were calculated and analyzed using The Institute for Genomic Research, TIGR Multiexperiment Viewer hierarchical clustering module [45].

### Protein expression and secretion

Splice variant cDNAs were subcloned into pcDNA3.1 (Invitrogen) with a Myc-His tag fused at the C-terminus of the proteins. To facilitate secretion, the native signal sequences of ASV derived from Met, FGFR1, VEGFR1, and RAGE were replaced by the tissue plasminogen activator signal/pro sequence (GenBank accession number NM_000930) by PCR cloning. All constructs were sequence verified, and were transiently expressed in HEK293 cells using LipofectAmine 2000 following the manufacturer's instruction (Invitrogen). Cell culture supernatants were collected 48 hours after transfection. To analyze expression of the recombinant proteins, equal amounts (20 μl) of supernatants were separated on SDS-PAGE gels. The separated proteins were transferred to nitrocellulose membranes, and were probed with anti-Myc antibody.

### Purification of recombinant Tie1-751

Tie1-751 was subcloned into pcDNA3.1 as described above with a Myc-His tag fused at the C-terminus of the proteins (Tie1-751(6His)). To construct Tie1-751-Fc, the Fc fragment of human IgG_1 _(from Pro100 to Lys330) was PCR amplified and fused inframe to the 3' end of Tie1-751 in the pcDNA 3.1 vector via restriction digestion using the *Xho*I-*Age*I site. Tie1-751(6His) and Tie1-751-Fc were transiently expressed in HEK 293 cells. Conditioned media were collected 72 hours later. Tie1-751(6His) was purified using a Ni-Sepharose 6 Fast Flow column (GE-Amersham, Piscataway, NJ, USA) and Tie1-751-Fc was purified using a Protein-A Sepharose column (GE-Amersham), following the manufacturer's instructions. Purity of the recombinant proteins was >95% as determined by SDS-PAGE and Coomassie Blue staining.

### Ligand binding

To determine whether the ASV bound their cognate ligands, 96-well assay plates were coated with VEGF-A, VEGF-C, platelet-derived growth factor (PDGF)-AB, hepatocyte growth factor, colony-stimulating factor (CSF), and Ang-1, respectively, at 4 μg/ml in PBS. The immobilized ligand-coated plates were used for binding of matched ASV in the same order, as follows: VEGFR1-541, VEGFR2-712, PDGFRβ-336, Met-877, CSF1R-306, and Tie1-751. In the case of VEGFR1-541, VEGFR2-712, PDGFRβ-336, Met-877, and CSF1R-306, supernatants from the ASV-expressing HEK293 cells were used for binding assays. The purified Tie1-751(6His) was used for Ang-1 binding. Binding was performed for 1.5 hours at room temperature followed by three rapid rinses in PBS/0.05% Tween-20. Bound ASV were detected using biotin-labeled, extracellular domain-specific antibodies.

### Binding of Tie1-751 to human umbilical vein endothelial cells

For cell surface binding of ^125^I-Tie1-751(6His), HUVEC were seeded into a 96-well plate at 1.4 × 10^4 ^cell/well in endothelial growth medium-2. Next day, medium was replaced with an ice-cold binding buffer (Hanks' balanced salt solution supplemented with 20 mM Hepes and 0.25% bovine serum albumin, pH 7.5). ^125^I-Tie1-751 was added to the binding buffer in the presence or absence of unlabeled Tie1-751. Binding was performed at 4°C for 1 hour followed by four washes with ice-cold PBS/0.05% Tween-20. A scintillation cocktail OptiPhase 'SuperMix' (PerkinElmer, Waltham, MA, USA) was added to each well, and the plates were read by Microbeta Trilux (PerkinElmer).

For direct binding of Tie1-751 to transmembrane Tie1 and Tie2, HUVEC were seeded into a six-well plate at 0.5 × 10^6^/well in endothelial growth medium-2. Next day, binding was carried out at 4°C for 1 hour in an ice-cold binding buffer (as above) containing 1 μM purified Tie1-751(6His). At the end of the binding, cells were treated with or without the membrane-impermeable chemical amine-reactive cross-linking agent DTSSP (3,3'-dithiobis [sulfosuccinimidylpropionate] (Pierce Biotechnology Inc., Rockford, IL, USA) at 1 mM for 30 minutes. This treatment was followed by inactivation of 3,3'-dithiobis(sulfosuccinimidylpropionate) with 20 mM Tris buffer, pH 7.5, for 15 minutes. Cells were subsequently lysed and immunoprecipitated using a C-terminal-specific anti-Tie1 or anti-Tie2 antibody. The immunoprecipitated proteins were analyzed by western blotting using anti-His antibody that recognizes the His-tagged Tie1-751.

### Evaluation of the therapeutic potential of alternative splice variants in a mouse model of arthritis

Ten-week-old DBA/1-Ola/Hsd mice (H-2^q ^haplotype; Harlan Laboratories UK Limited, Bicester, Oxon, UK) were immunized with purified bovine type II collagen prepared inhouse, and were emulsified with Freund's complete adjuvant, containing paraffin oil, and lyophilized *Mycobacterium tuberculosis H37 Ra *(Difco Becton Dickinson, Oxford, UK) [46]. Onset of arthritic disease was around 2 to 3 weeks later. ASV adenoviruses were administered intravenously (10^7 ^plaque-forming units/0.1 ml per mouse) via tail vein injection to mice on day 1 of arthritis.

All limbs were assessed daily and scored as follows: 1 = slight edema or erythema; 1.5 = edema and erythema involving at least some digits; 2 = frank edema/erythema involving the entire paw; and 2.5 = pronounced edema and erythema leading to incapacitated mobility [37,38]. A spring-loaded caliper (least detectable measure = 0.1 mm; Rohm GB Limited, Kingston-Upon-Thames, UK) was employed to measure the hind-paw thickness (mm) daily, which was expressed as the degree of paw swelling from day 1 of arthritis (Δ^mm^).

All murine work procedures had the approval of the local ethical review process committee, which followed the Helsinki Declaration Principles, and were carried out under Project Licence 70/5446.

For pharmacokinetic analysis, mice received tail vein injection of 1 × 10^9 ^plaque-forming units of Adv-Tie1-751(6His). Sera were taken after injection at the indicated times and were analyzed by SDS-PAGE followed by western blotting with anti-Tie1 antibody. Signals exposed onto an X-ray film in a visually estimated linear range were scanned and quantitated using Typhoon Trio instrument (GE-Amersham) and were compared with a known concentration of purified Tie1-751(6His).

### Histological evaluation of joint architecture

At the end of the 10-day period of monitoring, the hind feet of the mice were fixed in 10% buffered formalin solution, decalcified (Rapid-Cal™; BBC Biochemical, Dallas, TX, USA), embedded in paraffin wax positioned laterally and sagittally sectioned. Serial sections of 5 to 6 μm thickness were obtained, dewaxed and stained with H & E or toluidine blue.

The stained sections were scored for changes to joint architecture by an observer blinded to the study groups. Each section was screened for changes to the joint architecture, and every joint was scored as follows: normal; mild (minimal synovitis, some cartilage loss, shrinkage in the size of cartilage chondrocytes with denucleation, and bone erosions limited to discrete foci); moderate (more extensive synovial hyperplasia, destruction of large segments of the cartilage and considerable bone erosions caused by an invasive pannus front); and severe (complete destruction of the joint architecture).

### Statistical analysis

*P *values were determined using a two-tailed *t *test assuming unequal variances. Data on the progression of arthritic disease were analyzed using two-way analysis of variance. Histology data were analyzed by the chi-square test for trend.

## Results

### Cloning of novel alternative splice variants coding for secreted receptor isoforms

To identify novel splice variants from cell surface receptor genes, we performed RT-PCR using a complex mRNA pool representing major human tissue types and tumors. We intended to identify novel splice patterns that lead to the formation of secreted receptor isoforms. To do so, we selected forward PCR primers that flank the start codon and reverse primers that are located in the transmembrane regions. The amplified PCR products were separated on agarose gels and the DNA bands were extracted, purified, and individually cloned to generate gene-specific plasmid cDNA libraries. Two hundred to 1,000 random recombinant clones within each library were screened using PCR amplification to analyze the insert sizes. Clones with subtle differences in insert sizes on agarose gel electrophoresis were selected for complete DNA sequencing. Novel splice variants were identified by alignment of each cloned sequence to its respective genomic sequence in comparison with full-length transcripts of sequence databases of National Center for Biotechnology Information (NCBI) using the splice variant analysis software SIM4 [47]. Only transcripts with canonical donor–acceptor splicing sites (for example, GT–AG) were considered for further analysis, so that potential PCR artifacts were excluded. We defined a novel splice variant as an alteration in splice patterns to the existing full-length transcript sequences from available sequence databases, including Geneseq and other public databases.

**Figure 1:**
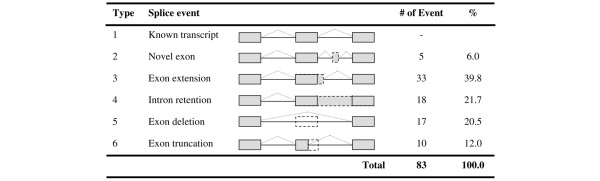
**Splice events categorized by type**. A total of 83 alternative splicing events were identified in the 21-gene array (Table 1). The identified splicing events fell into five listed types. The splice pattern of the known transcript is depicted as type 1.

A total of 60 full-length splice variants, derived from the extracellular domains of the 21 type 1 receptor genes, were confirmed to be novel – with variants from the c-Met proto-oncogene being the most diverse (Table 1). Sequences of the 60 full-length novel splice variants were deposited with GenBank (accession numbers EU826561 to EU826620; see also Additional files 1 and 2). Alignment of the cloned splice variant cDNA sequences with the corresponding genomic and known transcript sequences in available databases revealed that a total of 83 alternative splice events occurred in the 60 novel variants (Figure 1). We categorized the alternative splice events, and found that 67.5% led to intron fusion (intron sequences inserted into mature mRNA). These include novel exon insertion, exon extension, and intron retention. The remaining 32.5% of alternative splice events resulted in exon loss (a portion or whole exon was skipped). A total of 18% of the exon extensions and 50% of the exon truncations identified in this study occurred at the 5' end of the alternatively spliced exons. All of the 60 transcript variants encounter a stop codon within the extracellular regions. As a result, these variants encode soluble receptor isoforms, and were subsequently referred to as ASV.

**Table 1:** Cloned alternative splice variant mRNAs

Receptor (*n *= 21)	NCBI accession number	Novel alternative splice variants
VEGFR1	NM_002019	2
VEGFR2	NM_002253	1
VEGFR3	NM_002020	3
Met	NM_000246	15
Ron	NM_002447	4
Tie1	NM_005424	5
Tie2	NM_000459	2
CSF1R	NM_005211	1
Kit	NM_000222	1
PDGFRβ	NM_002609	1
FGFR1	M34641	2
FGFR2	NM_000141	4
FGFR4	NM_002011	2
EPHA1	NM_005232	2
EPHA2	NM_004431	1
EPHB1	NM_004441	1
EPHB4	NM_004444	3
IGFR1	NM_000875	2
DDR1	NM_013993	2
TNFR1β	NM_001066	1
RAGE	NP_001127	5
Total		60

Sixty novel alternative splice variants were cloned from 21 cell surface receptor genes by RT-PCR amplification followed by extensive colony screening. The number of novel alternative splice variants is presented for each receptor tested. NCBI, National Center for Biotechnology Information.

### Detection of alternative splice variant mRNA expression

**Figure 2:**
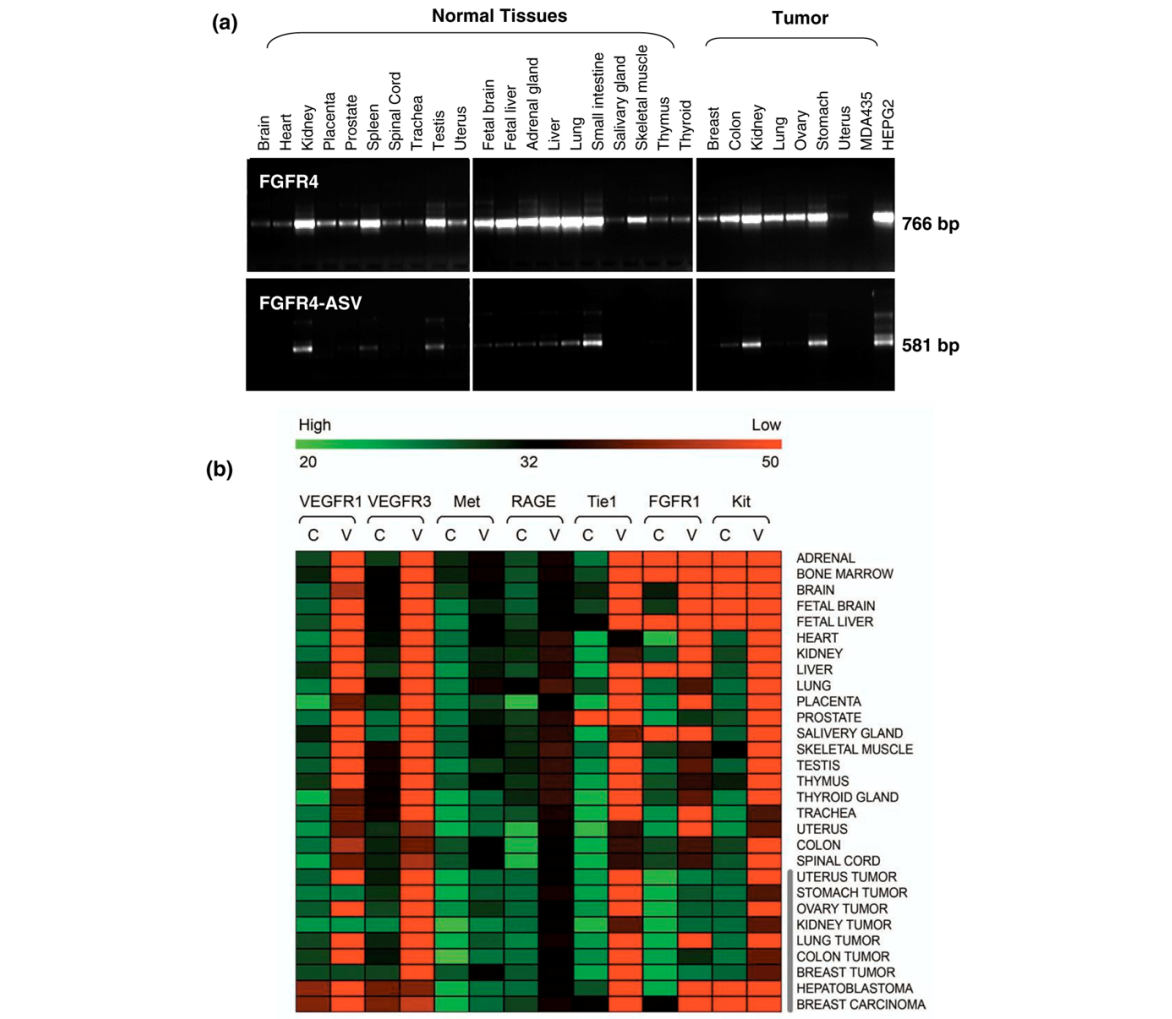
**Alternative splice variant mRNA expression**. **(a) **RT-PCR detection of mRNA expression of FGFR4 (top panels) and FGFR4-ASV (bottom panels) across 20 normal tissues and nine cancers, including two cancer cell lines. The amplified RT-PCR products were separated on 1% agarose gels and visualized by ethidium bromide staining. bp, base pairs. **(b) **Expression profile heat map of the constitutively expressed (C) and matched splice variant (V) mRNAs. Transcripts were analyzed across 20 normal tissues and nine cancers, including two cancer cell lines. Amplification of the constitutive and splice variant sequences was performed using real-time PCR. Bar shows a color shift from green (high-level expression) to red (low-level expression), with the corresponding cycle threshold values indicated.

Expression of ASV mRNA relative to their corresponding constitutively spliced transcripts was analyzed by both RT-PCR and quantitative RT-PCR. Amplification of each target sequence was performed across 29 distinct normal tissues as well as cancer tissues including two cancer cell lines. For PCR amplification of ASV, one primer was selected within the intron fusion sequence and the other from a remote exon encompassing several introns. This approach ensured that only the variant-specific mRNA transcript was amplified. An example of typical ASV mRNA expression (FGFR4) detected by RT-PCR is shown in Figure 2a.

For a better comparison of mRNA expression and tissue distribution, quantitative RT-PCR was performed to analyze ASV and their corresponding constitutively spliced transcripts. Our results demonstrated that expression of seven alternative splice variant mRNAs (VEGFR1, VEGFR3, Met, RAGE, Tie1, FGFR1, and Kit) is present in multiple normal and tumor tissues (Figure 2b). Levels of expression varied among tissues, with the ASV derived from VEGFR1, Met, and FGFR1 being predominantly expressed in tumor tissues. In contrast, ASV derived from VEGFR3 had the most restricted expression, and were observed only in a few normal tissues and cancer cell lines. These preliminary results indicate that expression of ASV is tissue specific and occurs more frequently in tumor than normal tissues.

### Ligand binding potential of recombinant alternative splice variants

Among the 60 ASV cloned, we selected 10 for initial functional testing (Table 2). The selected ASV (corresponding to ASV derived from VEGFR1, VEGFR2, VEGFR3, Tie1, Met, Kit, CSF1R, PDGFRβ, FGFR1, and RAGE) represent diverse members of gene families, possess known functional domains such as ligand binding domains, and encode novel amino acids compared with previously reported splice variant sequences.

**Table 2:** Alternative splice variants selected for functional testing

	Splice variant	Clone	Length of ORF^a^	Length of ECD^a^	C-terminal novel amino acids^b^
1	VEGFR1-541	018C02	541	758	LPPANSSFML PPTSFSSNYF HFLP*
2	VEGFR2-712	015F01	712	764	E*
3	VEGFR3-765	015G09	765	775	REGGPGEGQV RRPARPTIPN PGGPAPPPHP LQESTWRTPT RS*
4	Met-877	020H07	877	932	VRNALNTVLN HQLKLN*
5	Tie1-751	016G03	751	759	ERAGPTGPPG L*
6	CSF1R-306	005A06	306	512	GTPSPSLCPA *
7	c-Kit-413	002H01	413	520	SL*
8	PDGFRβ-336	007C09	336	531	RAATCGSWER WAHYNLLSCI GAGHCR*
9	FGFR1-320	022C02	320	374	GTHCNFSSRC PALATGTGGA CISRLGETQR QESWKNGLLP AWCHILPQL*
10	RAGE-387	021C06	387	342	IGETSPQALQ TLGLGCRTAQ ALISCPILAL SLTATPPLPP CTHTQASPAP PAFCQESSQA SPFFPLS*

Ten alternative splice variants were selected for functional testing. ^a^Lengths of the alternative splice variant open-reading frames (ORF) and lengths of the wildtype receptor extracellular domains (ECD) are indicated by the numbers of amino acids. ^b^Novel C-terminal amino acids of each alternative splice variant are shown. *Stop codon.

**Figure 3:**
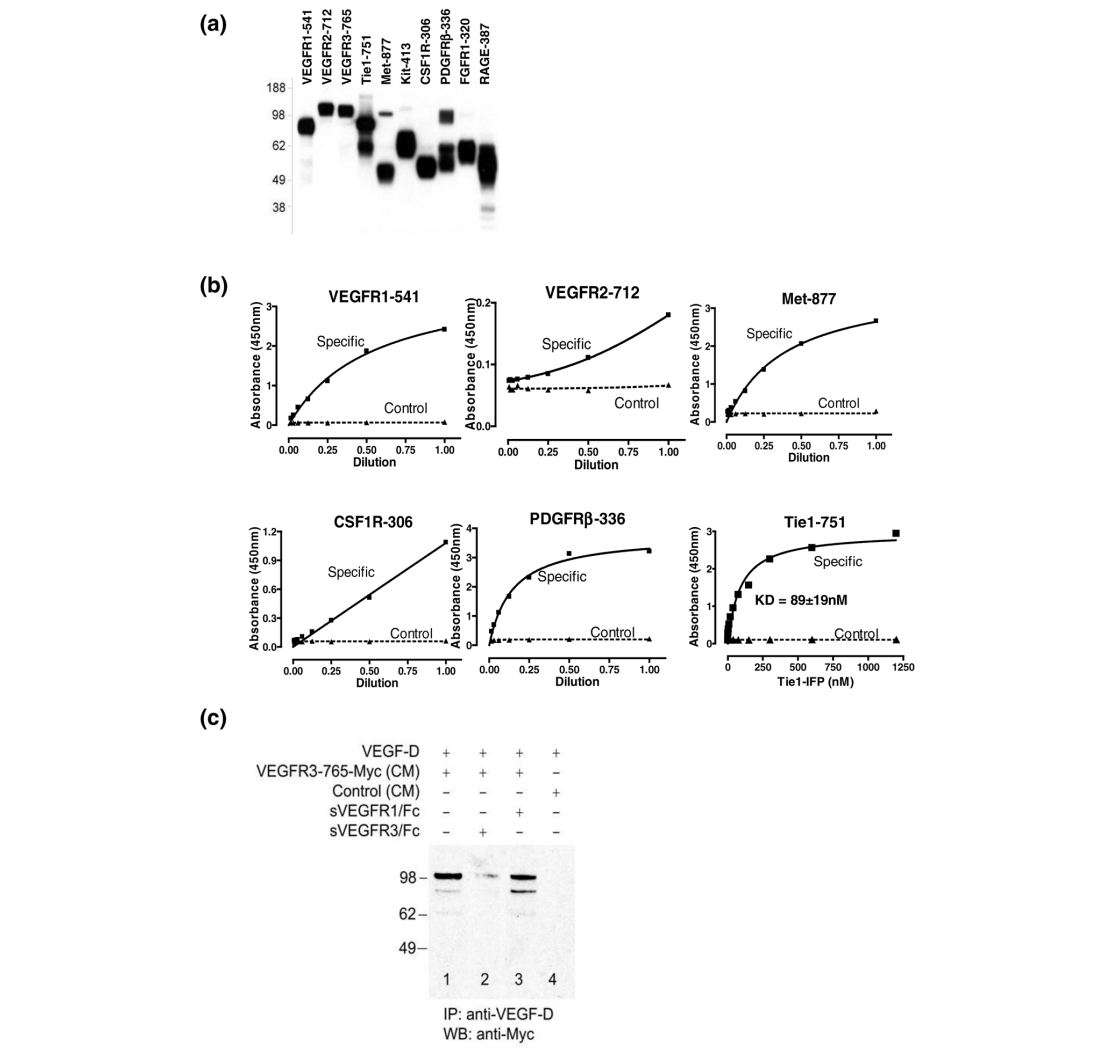
**Expression and ligand binding of recombinant alternative splice variants**. **(a) **HEK293 cells were transiently transfected with the indicated cDNA constructs. Conditioned media of HEK293 cells were collected after 48 hours, separated on SDS-PAGE gels and probed with an anti-Myc antibody to detect the Myc-tagged alternative splice variants (ASV). Molecular weights (kDa) are indicated. **(b) **For VEGFR1-541, VEGFR2-712, PDGFRβ-336, Met-877 and CSF1R-306, conditioned media from untransfected (Control, dashed lines) or ASV-transfected (Specific, solid lines) HEK293 cells were applied to plates precoated with the receptor-specific ligands. Unbound ASV were detected using antibodies against the extracellular domains of the receptors. Purified Tie1-751(6His) was used for Ang-1 binding, as above. Kd, dissociation constant. **(c) **Solution binding of VEGF-D to VEGFR3-765-Myc. Binding was carried out by combining VEGF-D with conditioned medium from either VEGFR3-765-Myc-expressing cells (lanes 1 to 3) or untransfected cells (lane 4). Subsequent immunoprecipitation was performed using anti-VEGF-D antibody and detected using anti-Myc antibody. To confirm the specificity of interaction between VEGF-D and VEGFR3-765-Myc, binding was performed in the presence of fivefold molar excess of either recombinant human VEGFR3/Fc chimera (lane 2) or soluble recombinant human VEGFR1/Fc chimera (lane 3). Molecular weights (kDa) are indicated. CM, Conditioned medium; IP, Immunprecipitation; WB, Western blot.

Efficient expression and secretion of the selected 10 recombinant ASV (VEGFR1, VEGFR2, VEGFR3, Tie1, Met, Kit, CSF1R, PDGFRβ, FGFR1, and RAGE) from HEK293 cells was confirmed by western blot analysis of the cell culture supernatants, using anti-Myc antibody to detected the Myc-tagged ASV (Figure 3a). Furthermore, we observed ligand binding by ASV proteins derived from VEGFR1, VEGFR2, PDGFRβ, Met, and CSF1R – which bound to VEGF-A, VEGF-C, PDGF, hepatocyte growth factor, and CSF, respectively (Figure 3b). For evaluation of Tie1-751, purified recombinant protein was used for binding to Ang-1, and a dissociation constant (Kd) of approximately 89nM was measured (Figure 3b).

Not all receptor–ligand interactions could be detected by plate-based binding, which may be a consequence of steric issues associated with binding receptor or ligand to the surface of the plate. Binding of VEGF-D to VEGFR3-765, for example, was demonstrated only when the assay was performed in solution (Figure 3c). Specificity of VEGF-D binding to VEGFR3-765 was confirmed using a soluble VEGFR3/Fc chimera, which was able to compete with VEGFR3-765 binding to VEGF-D – unlike a soluble VEGFR1/Fc chimera (Figure 3c).

### Tie1-751 binds to membrane Tie1 and Tie2 on human umbilical vein endothelial cells

**Figure 4:**
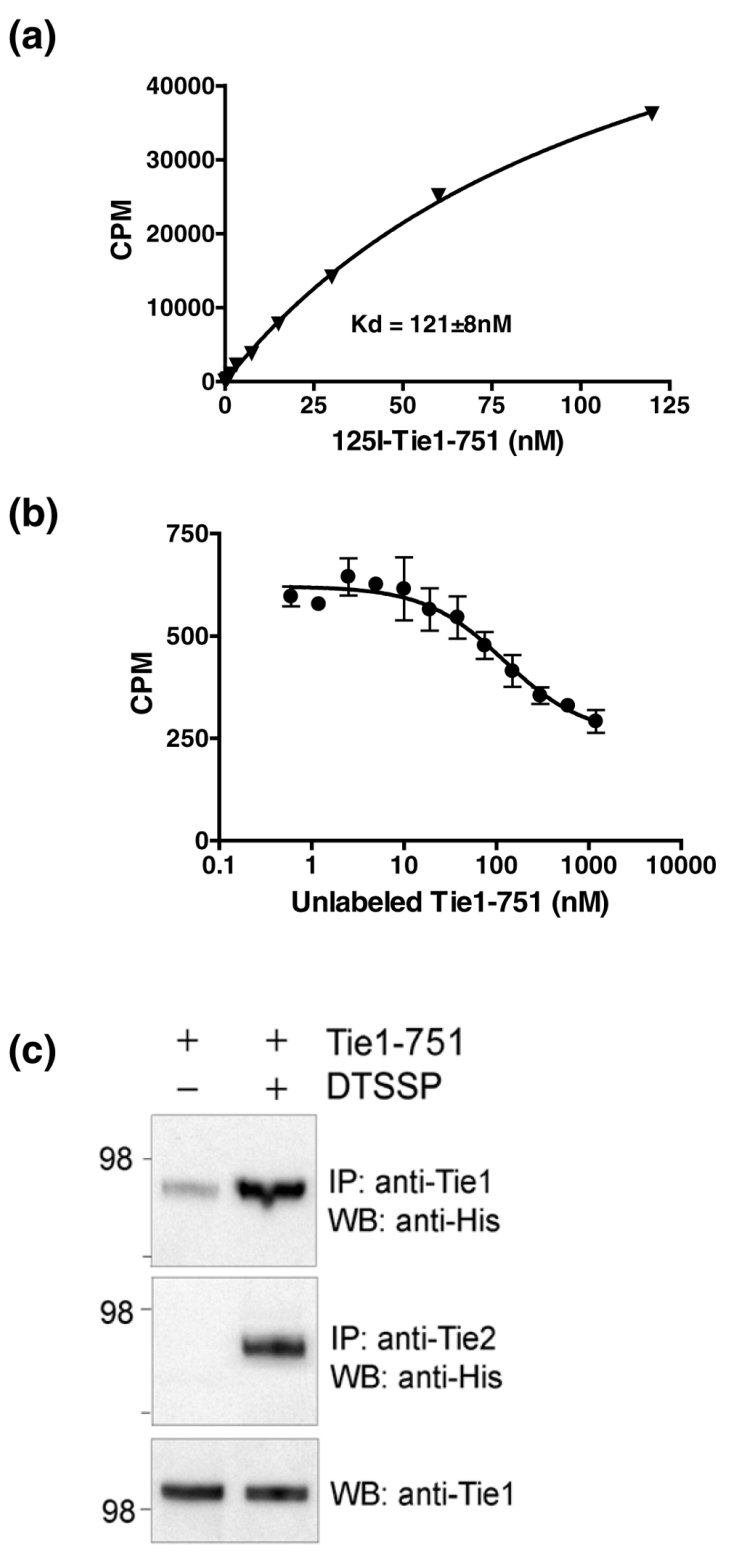
**Tie1-751 interacts with Tie1 and Tie2**. **(a) **Specific binding of ^125^I-Tie1-751(6His) to human umbilical vein endothelial cells (HUVEC). Nonspecific binding was determined in the presence of 100-fold excess of unlabelled Tie1-751 and was subtracted from the total binding. CPM, counts per minute; Kd, dissociation constant. **(b) **Binding of ^125^I-Tie1-751(6His) to HUVEC was competed by increasing amounts of cold Tie1-751. Data are the mean ± standard error of the mean. **(c) **Binding of Tie1-751(6His) to HUVEC. At the end of binding, cells were treated with or without the cross-linker 3,3'-dithiobis(sulfosuccinimidylpropionate) (DTSSP), immunoprecipitated using a C-terminal-specific anti-Tie1 (top panel) or anti-Tie2 (middle panel) antibody, and were analyzed by western blotting using anti-His antibody. To confirm equal loading, cell lysates were blotted with anti-Tie1 antibody (bottom panel). IP, Immunprecipitation; WB, Western blot.

Some soluble receptor splice variants have been shown to bind cognate cell surface receptors and to modulate response to ligand [48]. Tie1-751 comprises most of the extracellular domain of Tie1 plus 11 C-terminal intron-derived amino acids. To begin understanding the functionality of Tie1-751, we tested whether Tie1-751 binds to endothelial cells. Proliferating endothelial cells (HUVEC) were incubated with ^125^I-labeled Tie1-751. Our results showed that ^125^I-Tie1-751 specifically bound to HUVEC, with an estimated dissociation constant (Kd) of 121 nM (Figure 4a). Binding of ^125^I-Tie1-751 to HUVEC was competed by increasing amounts of unlabeled Tie1-751 (Figure 4b).

Direct binding of Tie1-751(6His) to Tie1 and Tie2 on HUVEC was also examined. Our results demonstrated interaction of Tie1-751(6His) with the transmembrane Tie1, as well as with the transmembrane Tie2 (Figure 4c).

### Evaluation of alternative splice variant activity in an *in vivo* model of arthritis

Since angiogenesis plays a key role in RA, we next evaluated the therapeutic potential of ASV in an extensively validated mouse model of arthritis – namely, acute CIA. On the day of disease onset, replication-incompetent alternative splice variant-expressing adenoviruses were administered as a single dose of 1 × 10^7 ^plaque-forming units. The severity of arthritis in the mice was consecutively recorded for the following 10 days.

**Figure 5:**
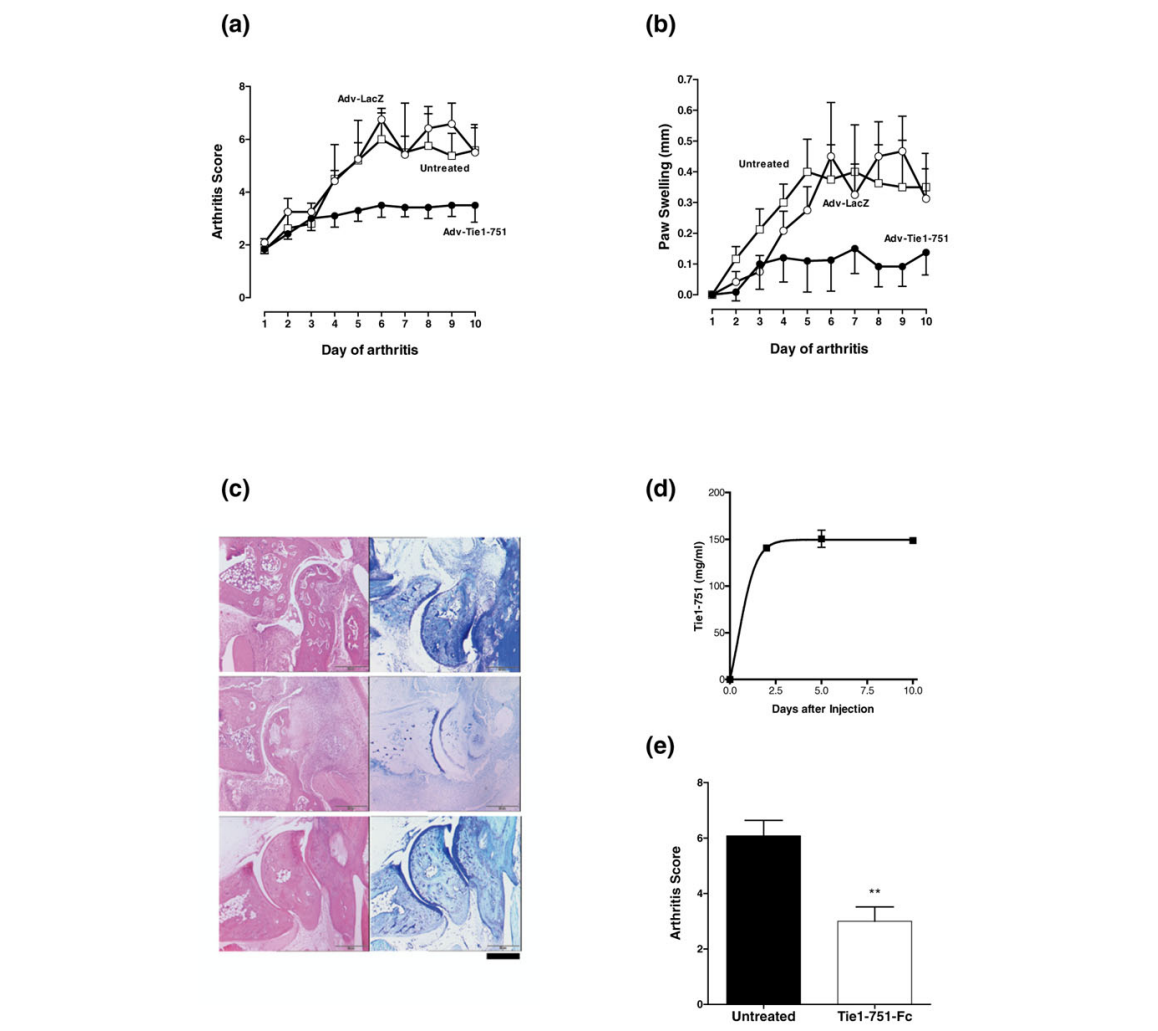
**Inhibition of murine collagen-induced arthritis by Tie1-751**. On the day of arthritis onset, mice received intravenously 1 × 10^7 ^plaque-forming units of adenoviruses expressing either LacZ (○) or Tie1-751 alternative splice variants (ASV) (●), or remained untreated (□) as indicated. **(a) **Clinical score was recorded daily, and data were analyzed by two-way analysis of variance versus untreated mice. LacZ, not significant (*P *= 0.3734); Tie1-751, *P *< 0.001; *n* = 6 per group. **(b) **Paw swelling was recorded using calipers daily, and data were analyzed by two-way analysis of variance versus untreated mice. LacZ, not significant (*P *= 0.5134); Tie1-751, *P *< 0.001. Data are means of *n *= 6. **(c) **Serial sections of mouse hind feet were stained with either H & E (left panels) or toluidine blue (right panels). Figure shows tibia–tarsus joint sections from untreated mice (top panels), from LacZ adenovirus-treated mice (middle panels), and from Tie1-751 ASV adenovirus-treated mice (bottom panels). Sections are shown at 40× magnification; scale bar = 20 μm. **(d) **Pharmacokinetics of Tie1-751 from the ASV-expressing adenovirus. Sera from untreated mice or mice treated intravenously with 1 × 10^9 ^plaque-forming units of Tie1-751 ASV adenovirus were analyzed after the indicated times by western blot, followed by scanning and quantitation using Tie1-751 standard. **(e) **Effect of recombinant Tie1-751-Fc protein on clinical score. Results are from mice on day 10 of arthritis. Filled bars, untreated mice; empty bars, mice treated with recombinant Tie1-751-Fc 30 mg/kg, three times weekly. Data are means of *n* = 6. **P < 0.01 for Tie-751-Fc treated mice versus untreated mice.

**Figure 6:**
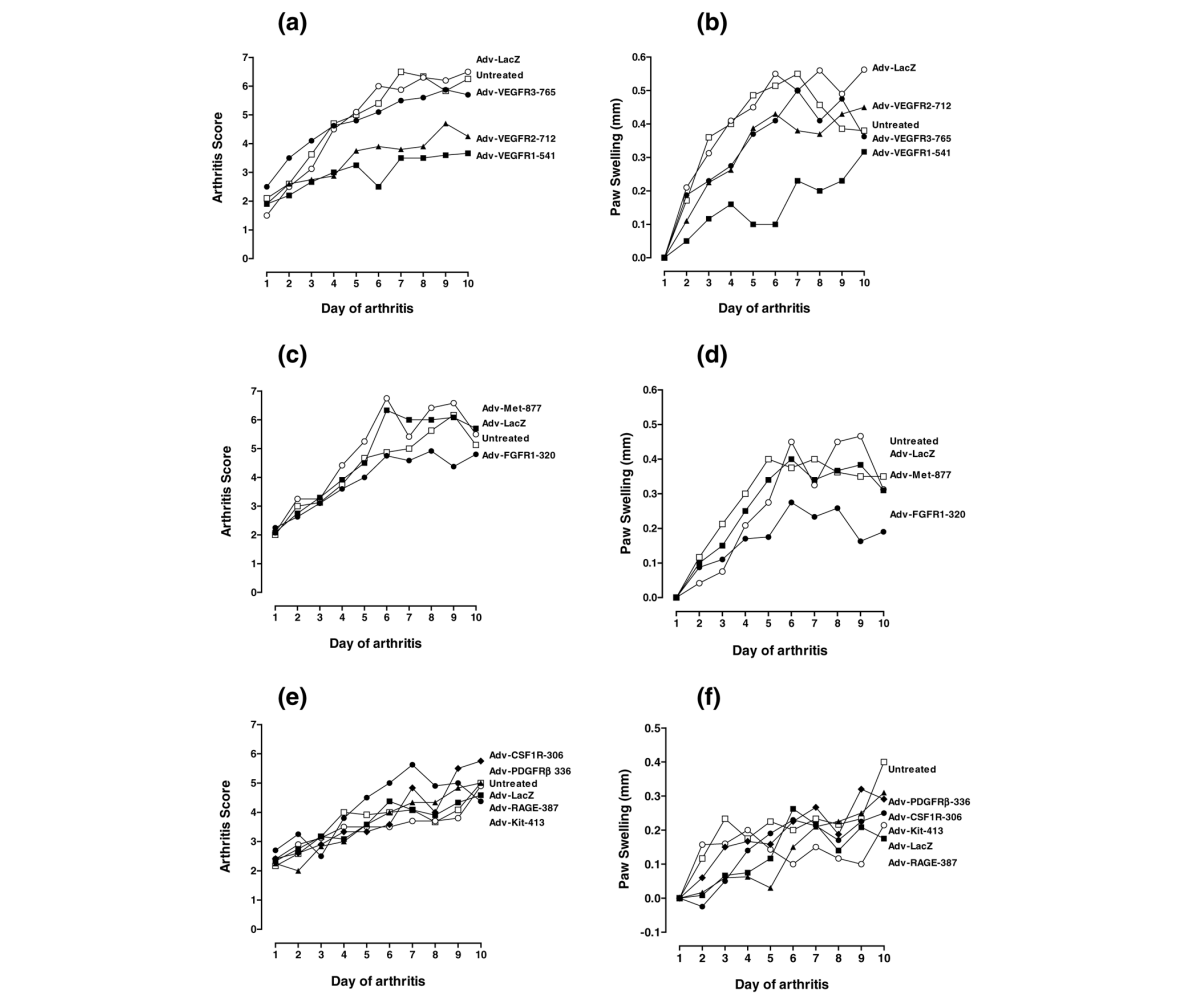
**Differential effects of alternative splice variant-expressing adenoviruses on collagen-induced arthritis mice**. On the day of arthritis onset, mice received intravenously 1 × 10^7 ^plaque-forming units of the indicated alternative splice variant (ASV)-expressing adenoviruses. Clinical scores (**(a)**, **(c)**, and** (e)**)and paw thickness measured by calipers (**(b)**, **(d)**, and** (f)**)were recorded daily. Data were analyzed by two-way analysis of variance versus untreated mice (Table 3). (a) and (b) Mice received adenoviruses expressing either LacZ (○), VEGFR1-541 (■), VEGFR2-712 (▲) or VEGFR3-765 (●), or remained untreated (□). Data are means of *n* = 5 per group. (c) and (d) Mice received adenoviruses expressing either LacZ (○), Met-877 (■), Tie1-751 (▲) or FGFR1-320 (●), or remained untreated (□). Data are means of *n* = 6 per group. (e) and (f) Mice received adenoviruses expressing either LacZ (○), RAGE-387 (■), PDGFRβ-366 (▲), c-Kit-413 (●), or CSF1R-306 (◆), or remained untreated (□). Data are means of *n* = 6 per group.

Control adenovirus (LacZ) was without significant effect on disease severity (Table 3 and Figures 5 and 6). In contrast, treatment with either Tie1-751 (Table 3 and Figure 5) or VEGFR1-541 (Table 3 and Figure 6) alternative splice variant adenoviruses significantly reduced disease severity, as evidenced by decreased clinical scores (*P *< 0.001), reduced paw thickness (*P *< 0.001), and reduced joint inflammation and destruction (*P *< 0.01 and *P *< 0.001 for VEGFR1-541 and Tie1-751, respectively). An example of the joint histology for untreated, LacZ ASV-treated and Tie1-751 ASV-treated mice is shown in Figure 5c, with quantitative analysis of the histology depicted in Table 3.

**Table 3:** Effect of alternative splice variant-expressing adenoviruses on joint inflammation and destruction

Alternative splice variant adenovirus	Mice per group (n)	Joints assessed (n)	*P *value		
			
			Clinical score	Paw swelling	Histological evaluation
Untreated	6	120	-	-	-
LacZ	6	164	0.4549	0.3759	0.3797
VEGFR1-541	5	53	<0.0001	<0.0001	0.0096
VEGFR2-712	5	44	<0.0001	0.1762	0.7340
VEGFR3-765	5	68	0.9366	0.2228	0.8148
Tie1-751	6	63	<0.0001	<0.0001	<0.001
Met-877	6	64	0.2924	0.6603	0.5038
c-Kit-413	6	55	0.0587	0.1501	0.1046
CSF1R-306	6	50	0.2448	0.5581	0.1510
PDGFRβ-336	6	41	0.8498	0.0632	0.8258
FGFR1-320	6	55	0.0044	0.0087	0.0568
RAGE-387	6	53	0.8543	0.1141	0.9799

Following onset of arthritis, mice were treated with the alternative splice variant adenovirus indicated. Data presented as *P *values of mice treated with the indicated recombinant alternative splice variant adenoviruses as compared with untreated mice, and are expressed as the *P *value of clinical scores, paw swelling, and histological evaluation. For clinical scores and paw swelling, data were analyzed using two-way analysis of variance versus untreated mice. For histological evaluation, H & E and toluidine blue stained sections were scored for pannus formation, synovitis, and bone and cartilage erosion. Data were analyzed using the chi-square test for trend *versus *untreated mice.

The presence of Tie1-751 in mouse sera was confirmed by western blotting (Figure 5d). The effectiveness of Tie1-751 in CIA was confirmed using recombinant Tie1-751-Fc protein (Figure 5e).

A less marked disease-modifying effect was seen with the adenovirus encoding FGFR1-320 (Table 3 and Figure 6), which reduced clinical scores and paw thickness (*P *< 0.01) but without achieving a statistically significant improvement of joint histological evaluation (*P *< 0.057). Similarly, VEGFR2-712 reduced the clinical score (*P *< 0.001) but failed to affect the paw thickness and the histological scores (Table 3 and Figure 6).

Treatment with ASV derived from VEGFR3, RAGE, Met, c-Kit, PDGFRβ, and CSF1R adenoviruses did not generate a significant effect on any of the disease parameters (Table 3 and Figure 6).

## Discussion

The proliferative and invasive nature of RA synovium has frequently led to comparisons with tumor development, and therefore the usefulness of VEGF blockade for treatment of certain cancers might be extrapolated to RA. Heterologous CIA in mice shares many features with RA, and has been widely used to study mechanisms involved in the arthritic process and to identify new strategies for RA treatment, such as TNFα inhibitors.

VEGF inhibition has been the focus of considerable clinically oriented research, and angiogenesis blockade has been shown to be effective in different *in vivo *models of arthritis, including CIA [18,20,36,49,50]. VEGF inhibition *in vivo*, however, is associated with side effects, such as impaired wound healing, hemorrhage, and gastrointestinal perforation. This is not surprising, given the heterozygous lethal phenotype of VEGF knockout mice [51], which suggests a strategic role for this molecule. Other positive regulators of angiogenesis expressed in RA include hepatocyte growth factor and PDGF [52,53]. To date, however, there have been no concerted efforts to compare a range of different antiangiogenic approaches side by side in a single study.

Bioinformatics surveys [11] and exon profiling [13,54] reveal that the majority of pre-mRNAs are alternatively spliced. As such, use of these soluble receptor variants might prove invaluable in designing new therapeutic strategies. We report here that, using an efficient approach, we cloned 60 novel ASV of 21 genes encoding RTKs and other cell surface receptors. The discovery of so many novel splice variants from a small group of well-characterized drug target genes is consistent with reports suggesting that alternative splicing is one of the most significant components generating protein and functional diversity in the human genome [13,54,55].

*In vivo*, soluble receptors are generated by both alternative pre-mRNA splicing and proteolytic cleavage (shedding) of membrane-anchored receptors, resulting in truncated molecules lacking a transmembrane domain and an intracellular segment. Soluble receptors may retain their ability to bind ligands and function as ligand antagonists [9]; for example, soluble TNF receptors [8] and soluble VEGFR1 [56]. Soluble receptors are often generated through rational engineering. A major difference between splice variant-derived soluble receptors and engineered soluble receptors is that the former contains novel amino acids and domain structures typically derived from intron fusion. These alterations may subsequently alter the functionality of the ASV as compared with the engineered or metalloprotease-generated soluble receptors. An example of altered function via alternative splicing is VEGF_165_b, an antiangiogenic factor derived from the alternative splicing of VEGF pre-mRNA [57]. VEGF_165_b antagonizes the angiogenic effect of VEGF_165_, which is also encoded by the VEGF gene. Further studies are required to elucidate the endogenous expression and function of the ASV described in this report.

Inhibiting angiogenesis is a promising strategy for treatment of neovascularization-related diseases [58], including RA [26]. Prior to anti-TNF therapeutics, 50% of RA patients become moderately disabled within 2 years and become severely disabled within 10 years of disease onset. The increasing use of anti-TNFα biological agents in RA is a major step forward, but its use is restricted by an associated risk of infection, including tuberculosis [1]. Most importantly, efficacy in longstanding treatment does not usually result in remission. As a consequence, initiatives to develop alternative treatments that control disease progression in RA are desirable.

A well-documented feature of RA is an alteration in the density of synovial blood vessels. Several angiogenic factors are expressed in RA, including VEGF, PDGF, fibroblast growth factor 1, and fibroblast growth factor 2, as well as Ang-1. Angiogenesis is a multistep process, however, and – while VEGF is important – other proangiogenic factors are also expressed in RA and CIA. The contribution of other proangiogenic factors to arthritic disease progression has not been well defined or compared directly within the same disease model. In the present study, 10 RTK-derived ASV were screened side by side in the high-throughput CIA model, using replication-incompetent adenoviruses as a delivery and *in vivo *expression system. This method allows for screening many candidate biologics quickly in a relevant disease model, without first expressing and purifying the target molecules, and will select for proteins that are significantly expressed and are bioactive across species barriers. Some candidate proteins may give false negative results because of issues related to expression and stability *in vivo*, a species barrier, or a lack of activity in the particular disease model.

*In vivo *screening of the ASV demonstrated clear differential effects. Among them, ASV derived from VEGFR1 and Tie1 were found to be the most potent. The effect of VEGFR1-541 ASV confirms our own previous data and that of others, demonstrating the effectiveness of VEGFR1 blockade in models of arthritis [18,20,33,50]. In contrast, blockade of VEGFR2 in models of arthritis has in general not been effective [33,50]. The effect of VEGFR2-712 ASV was modest in our study, with inhibition of clinical score but not of paw swelling or histological change. As the ultimate benefit of a potential therapeutic in RA would be joint protection and reduced edema, the fact that VEGFR2-712 ASV does not affect either paw swelling or joint inflammation/destruction supports the view that VEGFR2 blockade is not likely to be beneficial in RA.

Expression of both Ang-1 [39,40] and angiopoietin receptors Tie1 and Tie2 [41-43] in RA synovial tissue has been described. Ang-1 is chemotactic and weakly mitogenic for HUVEC [59,60], promotes formation of endothelial sprouts [61], and has been proposed to act in concert with VEGF to promote vascular network maturation [62,63]. Furthermore, Ang-1 was found to be a survival factor for endothelial cells, protecting HUVEC from apoptosis induced by serum withdrawal [64]. Angiopoietin signaling was until recently considered to be mediated via Tie2. The embryonic lethality of Tie1 knockout mice, however, suggested that Tie1 signaling is important in vascular network formation. It is now thought that Tie1 may modulate signaling through Tie2 [65-67]. Marron and colleagues reported that activation of Tie1 ectodomain cleavage increased activation of Tie2, which could potentially control signaling via Tie2 [68].

The novel activity of Tie1-751 in the CIA model [35,69] motivated us to further examine its mechanism of action. Our results demonstrate that Tie1-751 directly binds to Tie1 and Tie2 on the surface of endothelial cells. Binding of Tie1-Fc to transmembrane Tie1 and the interaction of transmembrane Tie1 and Tie2 at the cell surface have recently been reported [66]. The mechanism of binding Tie1-751 to Tie1 and to Tie2, however, is currently unknown. Our initial characterization also revealed that Tie1-751 inhibits Ang-1-induced Tie1 phosphorylation and the prosurvival effect of Ang-1 on HUVEC (data not shown). These results suggest that Tie1-751 may inhibit activation of the angiopoietin–Tie system by both sequestering ligand and forming nonsignaling heterodimers with cell surface receptors. It is possible that the C-terminal intron-encoded domain of Tie1-751 expands its functionality. Blocking Tie2 has been reported effective in CIA, but no such data are available for Tie1 inhibition [70].

Further work is needed to confirm the function of novel domains generated by alternative splicing. The differential effects of the 10 ASV in arthritis *in vivo*, however, suggest that selected ASV may have potential therapeutic application in RA and in other angiogenesis-dependent conditions.

## Conclusion

In summary, we describe an efficient method for the identification and determination of biologic activity of novel ASV derived from the cell surface receptor genes. Sixty ASV were identified. The variants identified commonly include unique amino acids forming additional protein domains. Those ASV tested were shown to bind cognate ligand. An alternative splice variant derived from Tie1 (Tie1-751) was shown to bind not only Ang-1 but also cell surface Tie1 and Tie2. Using replication-deficient adenoviruses as a means of screening for biologic activity, we showed that RTK-derived ASV have selective potential therapeutic activity in a murine model of RA. Furthermore, we have shown for the first time that inhibition of the angiopoietin–Tie axis can markedly reduce arthritis severity. The present work demonstrates that ASV are a potential source of novel regulatory proteins, which may have therapeutic potential in animal models of disease and warrant testing in humans.

## Abbreviations

Adv = adenovirus; Ang-1 = angiopoietin-1; ASV = alternative splice variants; CIA = collagen-induced arthritis; CSF = colony-stimulating factor; Fc = crystallizable fragment; FGFR = fibroblast growth factor receptor; H & E = hematoxylin and eosin; HUVEC = human umbilical vein endothelial cells; PBS = phosphate-buffered saline; PCR = polymerase chain reaction; PDGF = platelet-derived growth factor; PDGFRβ = platelet-derived growth factor receptor beta; RA = rheumatoid arthritis; RT = reverse transcriptase; RTK = receptor tyrosine kinase; Tie = tyrosine kinase with immunoglobulin and epidermal growth factor homology domains; TNF = tumor necrosis factor; VEGF = vascular endothelial growth factor; VEGFR = vascular endothelial growth factor receptor.

## Competing interests

PJ, JZ, IN, BJ, and HMS are employees of Receptor BioLogix, Inc. and hold stocks in the company, and declare competing financial interests. MF and EMP have acted as consultants for Receptor BioLogix, Inc. The other authors declare that they have no competing interests. Receptor BioLogix, Inc. holds the patents related to the content of the manuscript.

## Authors' contributions

HMS designed the study. PJ assisted in the study design, oversaw the project running and data analysis, and drafted the manuscript. PJ, JZ, IN, and BJ performed the alternative splice variant cloning, sequence analysis, protein expression and purification, and ligand/receptor binding assays. SP performed and analyzed the quantitative PCR experiment. EMP and MF assisted in the study design and coordination, and oversaw the data analysis and drafting of the manuscript. PFS and DC designed and carried out the *in vivo *arthritis studies. All authors read and approved the final manuscript.

## Supplementary Material

Additional file 1 A Word file Summarizing the information of the 60 full-length novel splice variants with GenBank accession numbers. Click here for file

Additional file 2 A Word file containing a table presenting the cDNA sequences of the 60 full-length novel splice variants that have been deposited with GenBank. Click here for file
