# Supplementary material for: Novel splice variants derived from the receptor tyrosine kinase superfamily are potential therapeutics for rheumatoid arthritis
Source: Arthritis Res Ther. 2008 Jul 1;10(4):R73. doi: 10.1186/ar2447 (PMC2575619; doi:10.1186/ar2447)
Supplement: Additional file 1 — A Word file Summarizing the information of the 60 full-length novel splice variants with GenBank accession numbers. [file ar2447-S1.doc]

**Supplemental Table 1**

| **GenBank Accession #** | **RBLX Clone ID** | **Splice Variant of Gene** | **Name of Splice Variant** |
| --- | --- | --- | --- |
| EU826561 | 018C02 | Splice Variant of VEGFR1 | VEGFR1-541 |
| EU826562 | 004C05 | Splice Variant of VEGFR1 | VEGFR1-174 |
| EU826563 | 015F01 | Splice Variant of VEGFR2 | VEGFR2-712 |
| EU826564 | 015G09 | Splice Variant of VEGFR3 | VEGFR3-765 |
| EU826565 | 007F05 | Splice Variant of VEGFR3 | VEGFR3-295 |
| EU826566 | 007E10 | Splice Variant of VEGFR3 | VEGFR3-227 |
| EU826567 | 020H07 | Splice Variant of MET | MET-877 |
| EU826568 | 020H06 | Splice Variant of MET | MET-823 |
| EU826569 | 020H03 | Splice Variant of MET | MET-755 |
| EU826570 | 020H08 | Splice Variant of MET | MET-764 |
| EU826571 | 020G07 | Splice Variant of MET | MET-661 |
| EU826572 | 020F11 | Splice Variant of MET | MET-719 |
| EU826573 | 020F12 | Splice Variant of MET | MET-697 |
| EU826574 | 020G03 | Splice Variant of MET | MET-691 |
| EU826575 | 020F08 | Splice Variant of MET | MET-664 |
| EU826576 | 020E11 | Splice Variant of MET | MET-621 |
| EU826577 | 020D07 | Splice Variant of MET | MET-598 |
| EU826578 | 020D11 | Splice Variant of MET | MET-408 |
| EU826579 | 020D04 | Splice Variant of MET | MET-518 |
| EU826580 | 020C12 | Splice Variant of MET | MET-468 |
| EU826581 | 020C10 | Splice Variant of MET | MET-413 |
| EU826582 | 004C11 | Splice Variant of RON | RON-495 |
| EU826583 | 014C01 | Splice Variant of RON | RON-541 |
| EU826584 | 014C09 | Splice Variant of RON | RON-908 |
| EU826585 | 014E12 | Splice Variant of RON | RON-647 |
| EU826586 | 006A04 | Splice Variant of TIE1 | TIE1-251 |
| EU826587 | 006B07 | Splice Variant of TIE1 | TIE1-379 |
| EU826588 | 006B10 | Splice Variant of TIE1 | TIE1-317 |
| EU826589 | 006B06 | Splice Variant of TIE1 | TIE1-161 |
| EU826590 | 016G03 | Splice Variant of TIE1 | TIE1-751 |
| EU826591 | 007G02 | Splice Variant of TIE2 | TIE2-367 |
| EU826592 | 007H03 | Splice Variant of TIE2 | TIE2-468 |

continued……….

| **GenBank Accession #** | **RBLX Clone ID** | **Splice Variant of Gene** | **Name of Splice Variant** |
| --- | --- | --- | --- |
| EU826593 | 005A06 | Splice Variant of CSF1R | CSF1R-306 |
| EU826594 | 002H01 | Splice Variant of KIT | KIT-413 |
| EU826595 | 007C09 | Splice Variant of PDGFRB | PDGFRB-336 |
| EU826596 | 001E12 | Splice Variant of FGFR1 | FGFR1-228 |
| EU826597 | 022C02 | Splice Variant of FGFR1 | FGFR1-320 |
| EU826598 | 022D06 | Splice Variant of FGFR2 | FGFR2-396 |
| EU826599 | 022C11 | Splice Variant of FGFR2 | FGFR2-317 |
| EU826600 | 022C10 | Splice Variant of FGFR2 | FGFR2-266 |
| EU826601 | 022D04 | Splice Variant of FGFR2 | FGFR2-281 |
| EU826602 | 002A11 | Splice Variant of FGFR4 | FGFR4-72 |
| EU826603 | 002A10 | Splice Variant of FGFR4 | FGFR4-446 |
| EU826604 | 004G03 | Splice Variant of EPHA1 | EPHA1-474 |
| EU826605 | 004H03 | Splice Variant of EPHA1 | EPHA1-490 |
| EU826606 | 016E12 | Splice Variant of EPHA2 | EPHA2-497 |
| EU826607 | 005D06 | Splice Variant of EPHB1 | EPHB1-242 |
| EU826608 | 012D11 | Splice Variant of EPHB4 | EPHB4-516 |
| EU826609 | 012C08 | Splice Variant of EPHB4 | EPHB4-306 |
| EU826610 | 012E11 | Splice Variant of EPHB4 | EPHB4-414 |
| EU826611 | 024B04 | Splice Variant of IGF1R | IGF1R-831 |
| EU826612 | 024A03 | Splice Variant of IGF1R | IGF1R-759 |
| EU826613 | 005A11 | Splice Variant of DDR1 | DDR1-286 |
| EU826614 | 005A10 | Splice Variant of DDR1 | DDR1-243 |
| EU826615 | 003H02 | Splice Variant of TNFR1B | TNFR1B-155 |
| EU826616 | 021A05 | Splice Variant of RAGE | RAGE-146 |
| EU826617 | 021F06 | Splice Variant of RAGE | RAGE-172 |
| EU826618 | 021C06 | Splice Variant of RAGE | RAGE-387 |
| EU826619 | 021C02 | Splice Variant of RAGE | RAGE-266 |
| EU826620 | 021A11 | Splice Variant of RAGE | RAGE-128 |
