# Supplementary material for: Novel splice variants derived from the receptor tyrosine kinase superfamily are potential therapeutics for rheumatoid arthritis
Source: Arthritis Res Ther. 2008 Jul 1;10(4):R73. doi: 10.1186/ar2447 (PMC2575619; doi:10.1186/ar2447)
Supplement: Additional file 2 — A Word file containing a table presenting the cDNA sequences of the 60 full-length novel splice variants that have been deposited with GenBank. [file ar2447-S2.doc]

>EU826561,018C02,splice variant of VEGFR1,VEGFR1-541

ATG GTC AGC TAC TGG GAC ACC GGG GTC CTG CTG TGC GCG CTG CTC

M V S Y W D T G V L L C A L L

AGC TGT CTG CTT CTC ACA GGA TCT AGT TCA GGT TCA AAA TTA AAA

S C L L L T G S S S G S K L K

GAT CCT GAA CTG AGT TTA AAA GGC ACC CAG CAC ATC ATG CAA GCA

D P E L S L K G T Q H I M Q A

GGC CAG ACA CTG CAT CTC CAA TGC AGG GGG GAA GCA GCC CAT AAA

G Q T L H L Q C R G E A A H K

TGG TCT TTG CCT GAA ATG GTG AGT AAG GAA AGC GAA AGG CTG AGC

W S L P E M V S K E S E R L S

ATA ACT AAA TCT GCC TGT GGA AGA AAT GGC AAA CAA TTC TGC AGT

I T K S A C G R N G K Q F C S

ACT TTA ACC TTG AAC ACA GCT CAA GCA AAC CAC ACT GGC TTC TAC

T L T L N T A Q A N H T G F Y

AGC TGC AAA TAT CTA GCT GTA CCT ACT TCA AAG AAG AAG GAA ACA

S C K Y L A V P T S K K K E T

GAA TCT GCA ATC TAT ATA TTT ATT AGT GAT ACA GGT AGA CCT TTC

E S A I Y I F I S D T G R P F

GTA GAG ATG TAC AGT GAA ATC CCC GAA ATT ATA CAC ATG ACT GAA

V E M Y S E I P E I I H M T E

GGA AGG GAG CTC GTC ATT CCC TGC CGG GTT ACG TCA CCT AAC ATC

G R E L V I P C R V T S P N I

ACT GTT ACT TTA AAA AAG TTT CCA CTT GAC ACT TTG ATC CCT GAT

T V T L K K F P L D T L I P D

GGA AAA CGC ATA ATC TGG GAC AGT AGA AAG GGC TTC ATC ATA TCA

G K R I I W D S R K G F I I S

AAT GCA ACG TAC AAA GAA ATA GGG CTT CTG ACC TGT GAA GCA ACA

N A T Y K E I G L L T C E A T

GTC AAT GGG CAT TTG TAT AAG ACA AAC TAT CTC ACA CAT CGA CAA

V N G H L Y K T N Y L T H R Q

ACC AAT ACA ATC ATA GAT GTC CAA ATA AGC ACA CCA CGC CCA GTC

T N T I I D V Q I S T P R P V

AAA TTA CTT AGA GGC CAT ACT CTT GTC CTC AAT TGT ACT GCT ACC

K L L R G H T L V L N C T A T

ACT CCC TTG AAC ACG AGA GTT CAA ATG ACC TGG AGT TAC CCT GAT

T P L N T R V Q M T W S Y P D

GAA AAA AAT AAG AGA GCT TCC GTA AGG CGA CGA ATT GAC CAA AGC

E K N K R A S V R R R I D Q S

AAT TCC CAT GCC AAC ATA TTC TAC AGT GTT CTT ACT ATT GAC AAA

N S H A N I F Y S V L T I D K

ATG CAG AAC AAA GAC AAA GGA CTT TAT ACT TGT CGT GTA AGG AGT

M Q N K D K G L Y T C R V R S

GGA CCA TCA TTC AAA TCT GTT AAC ACC TCA GTG CAT ATA TAT GAT

G P S F K S V N T S V H I Y D

AAA GCA TTC ATC ACT GTG AAA CAT CGA AAA CAG CAG GTG CTT GAA

K A F I T V K H R K Q Q V L E

ACC GTA GCT GGC AAG CGG TCT TAC CGG CTC TCT ATG AAA GTG AAG

T V A G K R S Y R L S M K V K

GCA TTT CCC TCG CCG GAA GTT GTA TGG TTA AAA GAT GGG TTA CCT

A F P S P E V V W L K D G L P

GCG ACT GAG AAA TCT GCT CGC TAT TTG ACT CGT GGC TAC TCG TTA

A T E K S A R Y L T R G Y S L

ATT ATC AAG GAC GTA ACT GAA GAG GAT GCA GGG AAT TAT ACA ATC

I I K D V T E E D A G N Y T I

TTG CTG AGC ATA AAA CAG TCA AAT GTG TTT AAA AAC CTC ACT GCC

L L S I K Q S N V F K N L T A

ACT CTA ATT GTC AAT GTG AAA CCC CAG ATT TAC GAA AAG GCC GTG

T L I V N V K P Q I Y E K A V

TCA TCG TTT CCA GAC CCG GCT CTC TAC CCA CTG GGC AGC AGA CAA

S S F P D P A L Y P L G S R Q

ATC CTG ACT TGT ACC GCA TAT GGT ATC CCT CAA CCT ACA ATC AAG

I L T C T A Y G I P Q P T I K

TGG TTC TGG CAC CCC TGT AAC CAT AAT CAT TCC GAA GCA AGG TGT

W F W H P C N H N H S E A R C

GAC TTT TGT TCC AAT AAT GAA GAG TCC TTT ATC CTG GAT GCT GAC

D F C S N N E E S F I L D A D

AGC AAC ATG GGA AAC AGA ATT GAG AGC ATC ACT CAG CGC ATG GCA

S N M G N R I E S I T Q R M A

ATA ATA GAA GGA AAG AAT AAG CTT CCA CCA GCT AAC AGT TCT TTC

I I E G K N K L P P A N S S F

ATG TTG CCA CCT ACA AGC TTC TCT TCC AAC TAC TTC CAT TTC CTT

M L P P T S F S S N Y F H F L

CCG TGA

P *

>EU826562,004C05,splice variant of VEGFR1,VEGFR1-174

ATG GTC AGC TAC TGG GAC ACC GGG GTC CTG CTG TGC GCG CTG CTC

M V S Y W D T G V L L C A L L

AGC TGT CTG CTT CTC ACA GGA TCT AGT TCA GGT TCA AAA TTA AAA

S C L L L T G S S S G S K L K

GAT CCT GAA CTG AGT TTA AAA GGC ACC CAG CAC ATC ATG CAA GCA

D P E L S L K G T Q H I M Q A

GGC CAG ACA CTG CAT CTC CAA TGC AGG GGG GAA GCA GCC CAT AAA

G Q T L H L Q C R G E A A H K

TGG TCT TTG CCT GAA ATG GTG AGT AAG GAA AGC GAA AGG CTG AGC

W S L P E M V S K E S E R L S

ATA ACT AAA TCT GCC TGT GGA AGA AAT GGC AAA CAA TTC TGC AGT

I T K S A C G R N G K Q F C S

ACT TTA ACC TTG AAC ACA GCT CAA GCA AAC CAC ACT GGC TTC TAC

T L T L N T A Q A N H T G F Y

AGC TGC AAA TAT CTA GCT GTA CCT ACT TCA AAG AAG AAG GAA ACA

S C K Y L A V P T S K K K E T

GAA TCT GCA ATC TAT ATA TTT ATT AGT GAT ACA GGT AGA CCT TTC

E S A I Y I F I S D T G R P F

GTA GAG ATG TAC AGT GAA ATC CCC GAA ATT ATA CAC ATG ACT GAA

V E M Y S E I P E I I H M T E

GGA AGG GAG CTC GTC ATT CCC TGC CGG GTT ACG TCA CCT AAC ATC

G R E L V I P C R V T S P N I

ACT GTT ACT TTA AAA AAG AAa AAG GCA TAA

T V T L K K K K A *

>EU826563,015F01,splice variant of VEGFR2,VEGFR2-712

ATG CAG AGC AAG GTG CTG CTG GCC GTC GCC CTG TGG CTC TGC GTG

M Q S K V L L A V A L W L C V

GAG ACC CGG GCC GCC TCT GTG GGT TTG CCT AGT GTT TCT CTT GAT

E T R A A S V G L P S V S L D

CTG CCC AGG CTC AGC ATA CAA AAA GAC ATA CTT ACA ATT AAG GCT

L P R L S I Q K D I L T I K A

AAT ACA ACT CTT CAA ATT ACT TGC AGG GGA CAG AGG GAC TTG GAC

N T T L Q I T C R G Q R D L D

TGG CTT TGG CCC AAT AAT CAG AGT GGC AGT GAG CAA AGG GTG GAG

W L W P N N Q S G S E Q R V E

GTG ACT GAG TGC AGC GAT GGC CTC TTC TGT AAG ACA CTC ACA ATT

V T E C S D G L F C K T L T I

CCA AAA GTG ATC GGA AAT GAC ACT GGA GCC TAC AAG TGC TTC TAC

P K V I G N D T G A Y K C F Y

CGG GAA ACT GAC TTG GCC TCG GTC ATT TAT GTC TAT GTT CAA GAT

R E T D L A S V I Y V Y V Q D

TAC AGA TCT CCA TTT ATT GCT TCT GTT AGT GAC CAA CAT GGA GTC

Y R S P F I A S V S D Q H G V

GTG TAC ATT ACT GAG AAC AAA AAC AAA ACT GTG GTG ATT CCA TGT

V Y I T E N K N K T V V I P C

CTC GGG TCC ATT TCA AAT CTC AAC GTG TCA CTT TGT GCA AGA TAC

L G S I S N L N V S L C A R Y

CCA GAA AAG AGA TTT GTT CCT GAT GGT AAC AGA ATT TCC TGG GAC

P E K R F V P D G N R I S W D

AGC AAG AAG GGC TTT ACT ATT CCC AGC TAC ATG ATC AGC TAT GCT

S K K G F T I P S Y M I S Y A

GGC ATG GTC TTC TGT GAA GCA AAA ATT AAT GAT GAA AGT TAC CAG

G M V F C E A K I N D E S Y Q

TCT ATT ATG TAC ATA GTT GTC GTT GTA GGG TAT AGG ATT TAT GAT

S I M Y I V V V V G Y R I Y D

GTG GTT CTG AGT CCG TCT CAT GGA ATT GAA CTA TCT GTT GGA GAA

V V L S P S H G I E L S V G E

AAG CTT GTC TTA AAT TGT ACA GCA AGA ACT GAA CTA AAT GTG GGG

K L V L N C T A R T E L N V G

ATT GAC TTC AAC TGG GAA TAC CCT TCT TCG AAG CAT CAG CAT AAG

I D F N W E Y P S S K H Q H K

AAA CTT GTA AAC CGA GAC CTA AAA ACC CAG TCT GGG AGT GAG ATG

K L V N R D L K T Q S G S E M

AAG AAA TTT TTG AGC ACC TTA ACT ATA GAT GGT GTA ACC CGG AGT

K K F L S T L T I D G V T R S

GAC CAA GGA TTG TAC ACC TGT GCA GCA TCC AGT GGG CTG ATG ACC

D Q G L Y T C A A S S G L M T

AAG AAG AAC AGC ACA TTT GTC AGG GTC CAT GAA AAA CCT TTT GTT

K K N S T F V R V H E K P F V

GCT TTT GGA AGT GGC ATG GAA TCT CTG GTG GAA GCC ACG GTG GGG

A F G S G M E S L V E A T V G

GAG CGT GTC AGA ATC CCT GCG AAG TAC CTT GGT TAC CCA CCC CCA

E R V R I P A K Y L G Y P P P

GAA ATA AAA TGG TAT AAA AAT GGA ATA CCC CTT GAG TCC AAT CAC

E I K W Y K N G I P L E S N H

ACA ATT AAA GCG GGG CAT GTA CTG ACG ATT ATG GAA GTG AGT GAA

T I K A G H V L T I M E V S E

AGA GAC ACA GGA AAT TAC ACT GTC ATC CTT ACC AAT CCC ATT TCA

R D T G N Y T V I L T N P I S

AAG GAG AAG CAG AGC CAT GTG GTC TCT CTG GTT GTG TAT GTC CCA

K E K Q S H V V S L V V Y V P

CCC CAG ATT GGT GAG AAA TCT CTA ATC TCT CCT GTG GAT TCC TAC

P Q I G E K S L I S P V D S Y

CAG TAC GGC ACC ACT CAA ACG CTG ACA TGT ACG GTC TAT GCC ATT

Q Y G T T Q T L T C T V Y A I

CCT CCC CCG CAT CAC ATC CAC TGG TAT TGG CAG TTG GAG GAA GAG

P P P H H I H W Y W Q L E E E

TGC GCC AAC GAG CCC AGC CAA GCT GTC TCA GTG ACA AAC CCA TAC

C A N E P S Q A V S V T N P Y

CCT TGT GAA GAA TGG AGA AGT GTG GAG GAC TTC CAG GGA GGA AAT

P C E E W R S V E D F Q G G N

AAA ATT GAA GTT AAT AAA AAT CAA TTT GCT CTA ATT GAA GGA AAA

K I E V N K N Q F A L I E G K

AAC AAA ACT GTA AGT ACC CTT GTT ATC CAA GCG GCA AAT GTG TCA

N K T V S T L V I Q A A N V S

GCT TTG TAC AAA TGT GAA GCG GTC AAC AAA GTC GGG AGA GGA GAG

A L Y K C E A V N K V G R G E

AGG GTG ATC TCC TTC CAC GTG ACC AGG GGT CCT GAA ATT ACT TTG

R V I S F H V T R G P E I T L

CAA CCT GAC ATG CAG CCC ACT GAG CAG GAG AGC GTG TCT TTG TGG

Q P D M Q P T E Q E S V S L W

TGC ACT GCA GAC AGA TCT ACG TTT GAG AAC CTC ACA TGG TAC AAG

C T A D R S T F E N L T W Y K

CTT GGC CCA CAG CCT CTG CCA ATC CAT GTG GGA GAG TTG CCC ACA

L G P Q P L P I H V G E L P T

CCT GTT TGC AAG AAC TTG GAT ACT CTT TGG AAA TTG AAT GCC ACC

P V C K N L D T L W K L N A T

ATG TTC TCT AAT AGC ACA AAT GAC ATT TTG ATC ATG GAG CTT AAG

M F S N S T N D I L I M E L K

AAT GCA TCC TTG CAG GAC CAA GGA GAC TAT GTC TGC CTT GCT CAA

N A S L Q D Q G D Y V C L A Q

GAC AGG AAG ACC AAG AAA AGA CAT TGC GTG GTC AGG CAG CTC ACA

D R K T K K R H C V V R Q L T

GTC CTA GAG CGT GTG GCA CCC ACG ATC ACA GGA AAC CTG GAG AAT

V L E R V A P T I T G N L E N

CAG ACG ACA AGT ATT GGG GAA AGC ATC GAA GTC TCA TGC ACG GCA

Q T T S I G E S I E V S C T A

TCT GGG AAT CCC CCT CCA CAG ATC ATG TGG TTT AAA GAT AAT GAG

S G N P P P Q I M W F K D N E

ACC CTT GTA GAA GAC TCA GAG tga

T L V E D S E *

>EU826564,015G09,splice variant of VEGFR3,VEGFR3-765

ATG CAG CGG GGC GCC GCG CTG TGC CTG CGA CTG TGG CTC TGC CTG

M Q R G A A L C L R L W L C L

GGA CTC CTG GAC GGC CTG GTG AGT GGC TAC TCC ATG ACC CCC CCG

G L L D G L V S G Y S M T P P

ACC TTG AAC ATC ACG GAG GAG TCA CAC GTC ATC GAC ACC GGT GAC

T L N I T E E S H V I D T G D

AGC CTG TCC ATC TCC TGC AGG GGA CAG CAC CCC CTC GAG TGG GCT

S L S I S C R G Q H P L E W A

TGG CCA GGA GCT CAG GAG GCG CCA GCC ACC GGA GAC AAG GAC AGC

W P G A Q E A P A T G D K D S

GAG GAC ACG GGG GTG GTG CGA GAC TGC GAG GGC ACA GAC GCC AGG

E D T G V V R D C E G T D A R

CCC TAC TGC AAG GTG TTG CTG CTG CAC GAG GTA CAT GCC AAC GAC

P Y C K V L L L H E V H A N D

ACA GGC AGC TAC GTC TGC TAC TAC AAG TAC ATC AAG GCA CGC ATC

T G S Y V C Y Y K Y I K A R I

GAG GGC ACC ACG GCC GCC AGC TCC TAC GTG TTC GTG AGA GAC TTT

E G T T A A S S Y V F V R D F

GAG CAG CCA TTC ATC AAC AAG CCT GAC ACG CTC TTG GTC AAC AGG

E Q P F I N K P D T L L V N R

AAG GAC GCC ATG TGG GTG CCC TGT CTG GTG TCC ATC CCC GGC CTC

K D A M W V P C L V S I P G L

AAT GTC ACG CTG CGC TCG CAA AGC TCG GTG CTG TGG CCA GAC GGG

N V T L R S Q S S V L W P D G

CAG GAG GTG GTG TGG GAT GAC CGG CGG GGC ATG CTC GTG TCC ACG

Q E V V W D D R R G M L V S T

CCA CTG CTG CAC GAT GCC CTG TAC CTG CAG TGC GAG ACC ACC TGG

P L L H D A L Y L Q C E T T W

GGA GAC CAG GAC TTC CTT TCC AAC CCC TTC CTG GTG CAC ATC ACA

G D Q D F L S N P F L V H I T

GGC AAC GAG CTC TAT GAC ATC CAG CTG TTG CCC AGG AAG TCG CTG

G N E L Y D I Q L L P R K S L

GAG CTG CTG GTA GGG GAG AAG CTG GTC CTG AAC TGC ACC GTG TGG

E L L V G E K L V L N C T V W

GCT GAG TTT AAC TCA GGT GTC ACC TTT GAC TGG GAC TAC CCA GGG

A E F N S G V T F D W D Y P G

AAG CAG GCA GAG CGG GGT AAG TGG GTG CCC GAG CGA CGC TCC CAG

K Q A E R G K W V P E R R S Q

CAG ACC CAC ACA GAA CTC TCC AGC ATC CTG ACC ATC CAC AAC GTC

Q T H T E L S S I L T I H N V

AGC CAG CAC GAC CTG GGC TCG TAT GTG TGC AAG GCC AAC AAC GGC

S Q H D L G S Y V C K A N N G

ATC CAG CGA TTT CGG GAG AGC ACC GAG GTC ATT GTG CAT GAA AAT

I Q R F R E S T E V I V H E N

CCC TTC ATC AGC GTC GAG TGG CTC AAA GGA CCC ATC CTG GAG GCC

P F I S V E W L K G P I L E A

ACG GCA GGA GAC GAG CTG GTG AAG CTG CCC GTG AAG CTG GCA GCG

T A G D E L V K L P V K L A A

TAC CCC CCG CCC GAG TTC CAG TGG TAC AAG GAT GGA AAG GCA CTG

Y P P P E F Q W Y K D G K A L

TCC GGG CGC CAC AGT CCA CAT GCC CTG GTG CTC AAG GAG GTG ACA

S G R H S P H A L V L K E V T

GAG GCC AGC ACA GGC ACC TAC ACC CTC GCC CTG TGG AAC TCC GCT

E A S T G T Y T L A L W N S A

GCT GGC CTG AGG CGC AAC ATC AGC CTG GAG CTG GTG GTG AAT GTG

A G L R R N I S L E L V V N V

CCC CCC CAG ATA CAT GAG AAG GAG GCC TCC TCC CCC AGC ATC TAC

P P Q I H E K E A S S P S I Y

TCG CGT CAC AGC CGC CAG GCC CTC ACC TGC ACG GCC TAC GGG GTG

S R H S R Q A L T C T A Y G V

CCC CTG CCT CTC AGC ATC CAG TGG CAC TGG CGG CCC TGG ACA CCC

P L P L S I Q W H W R P W T P

TGC AAG ATG TTT GCC CAG CGT AGT CTC CGG CGG CGG CAG CAG CAA

C K M F A Q R S L R R R Q Q Q

GAC CTC ATG CCA CAG TGC CGT GAC TGG AGG GCG GTG ACC ACG CAG

D L M P Q C R D W R A V T T Q

GAT GCC GTG AAC CCC ATC GAG AGC CTG GAC ACC TGG ACC GAG TTT

D A V N P I E S L D T W T E F

GTG GAG GGA AAG AAT AAG ACT GTG AGC AAG CTG GTG ATC CAG AAT

V E G K N K T V S K L V I Q N

GCC AAC GTG TCT GCC ATG TAC AAG TGT GTG GTC TCC AAC AAG GTG

A N V S A M Y K C V V S N K V

GGC CAG GAT GAG CGG CTC ATC TAC TTC TAT GTG ACC ACC ATC CCC

G Q D E R L I Y F Y V T T I P

GAC GGC TTC ACC ATC GAA TCC AAG CCA TCC GAG GAG CTA CTA GAG

D G F T I E S K P S E E L L E

GGC CAG CCG GTG CTC CTG AGC TGC CAA GCC GAC AGC TAC AAG TAC

G Q P V L L S C Q A D S Y K Y

GAG CAT CTG CGC TGG TAC CGC CTC AAC CTG TCC ACG CTG CAC GAT

E H L R W Y R L N L S T L H D

GCG CAC GGG AAC CCG CTT CTG CTC GAC TGC AAG AAC GTG CAT CTG

A H G N P L L L D C K N V H L

TTC GCC ACC CCT CTG GCC GCC AGC CTG GAG GAG GTG GCA CCT GGG

F A T P L A A S L E E V A P G

GCG CGC CAC GCC ACG CTC AGC CTG AGT ATC CCC CGC GTC GCG CCC

A R H A T L S L S I P R V A P

GAG CAC GAG GGC CAC TAT GTG TGC GAA GTG CAA GAC CGG CGC AGC

E H E G H Y V C E V Q D R R S

CAT GAC AAG CAC TGC CAC AAG AAG TAC CTG TCG GTG CAG GCC CTG

H D K H C H K K Y L S V Q A L

GAA GCC CCT CGG CTC ACG CAG AAC TTG ACC GAC CTC CTG GTG AAC

E A P R L T Q N L T D L L V N

GTG AGC GAC TCG CTG GAG ATG CAG TGC TTG GTG GCC GGA GCG CAC

V S D S L E M Q C L V A G A H

GCG CCC AGC ATC GTG TGG TAC AAA GAC GAG AGG CTG CTG GAG GAA

A P S I V W Y K D E R L L E E

AAG TCT Ggt AGG GAG GGT GGC CCT GGC GAA GGG CAG GTC CGG AGG

K S G R E G G P G E G Q V R R

CCC GCG AGG CCG ACG ATC CCA AAC CCA GGT GGA CCC GCA CCT CCA

P A R P T I P N P G G P A P P

CCC CAC CCC CTG Cag GAG TCG ACT TGG CGG ACT CCA ACC AGA AGC

P H P L Q E S T W R T P T R S

tga

*

>EU826565,007F05,splice variant of VEGFR3,VEGFR3-295

ATG CAG CGG GGC GCC GCG CTG TGC CTG CGA CTG TGG CTC TGC CTG

M Q R G A A L C L R L W L C L

GGA CTC CTG GAC GGC CTG GTG AGT GGC TAC TCC ATG ACC CCC CCG

G L L D G L V S G Y S M T P P

ACC TTG AAC ATC ACG GAG GAG TCA CAC GTC ATC GAC ACC GGT GAC

T L N I T E E S H V I D T G D

AGC CTG TCC ATC TCC TGC AGG GGA CAG CAC CCC CTC GAG TGG GCT

S L S I S C R G Q H P L E W A

TGG CCA GGA GCT CAG GAG GCG CCA GCC ACC GGA GAC AAG GAC AGC

W P G A Q E A P A T G D K D S

GAG GAC ACG GGG GTG GTG CGA GAC TGC GAG GGC ACA GAC GCC AGG

E D T G V V R D C E G T D A R

CCC TAC TGC AAG GTG TTG CTG CTG CAC GAG GTA CAT GCC AAC GAC

P Y C K V L L L H E V H A N D

ACA GGC AGC TAC GTC TGC TAC TAC AAG TAC ATC AAG GCA CGC ATC

T G S Y V C Y Y K Y I K A R I

GAG GGC ACC ACG GCC GCC AGC TCC TAC GTG TTC GTG AGA GAC TTT

E G T T A A S S Y V F V R D F

GAG CAG CCA TTC ATC AAC AAG CCT GAC ACG CTC TTG GTC AAC AGG

E Q P F I N K P D T L L V N R

AAG GAC GCC ATG TGG GTG CCC TGT CTG GTG TCC ATC CCC GGC CTC

K D A M W V P C L V S I P G L

AAT GTC ACG CTG CGC TCG CAA AGC TCG GTG CTG TGG CCA GAC GGG

N V T L R S Q S S V L W P D G

CAG GAG GTG GTG TGG GAT GAC CGG CGG GGC ATG CTC GTG TCC ACG

Q E V V W D D R R G M L V S T

CCA CTG CTG CAC GAT GCC CTG TAC CTG CAG TGC GAG ACC ACC TGG

P L L H D A L Y L Q C E T T W

GGA GAC CAG GAC TTC CTT TCC AAC CCC TTC CTG GTG CAC ATC ACA

G D Q D F L S N P F L V H I T

GGC AAC GAG CTC TAT GAC ATC CAG CTG TTG CCC AGG AAG TCG CTG

G N E L Y D I Q L L P R K S L

GAG CTG CTG GTA GGG GAG AAG CTG GTC CTG AAC TGC ACC GTG TGG

E L L V G E K L V L N C T V W

GCT GAG TTT AAC TCA GGT GTC ACC TTT GAC TGG GAC TAC CCA GGG

A E F N S G V T F D W D Y P G

AAG CAG AAA ATC CCT TCA TCA GCG TCG AGT GGC TCA AAG GAC CCA

K Q K I P S S A S S G S K D P

TCC TGG AGG CCA CGG CAG GAG ACG AGC TGG TGA

S W R P R Q E T S W *

>EU826566,007E10,splice variant of VEGFR3,VEGFR3-227

ATG CAG CGG GGC GCC GCG CTG TGC CTG CGA CTG TGG CTC TGC CTG

M Q R G A A L C L R L W L C L

GGA CTC CTG GAC GGC CTG GTG AGT GGC TAC TCC ATG ACC CCC CCG

G L L D G L V S G Y S M T P P

ACC TTG AAC ATC ACG GAG GAG TCA CAC GTC ATC GAC ACC GGT GAC

T L N I T E E S H V I D T G D

AGC CTG TCC ATC TCC TGC AGG GGA CAG CAC CCC CTC GAG TGG GCT

S L S I S C R G Q H P L E W A

TGG CCA GGA GCT CAG GAG GCG CCA GCC ACC GGA GAC AAG GAC AGC

W P G A Q E A P A T G D K D S

GAG GAC ACG GGG GTG GTG CGA GAC TGC GAG GGC ACA GAC GCC AGG

E D T G V V R D C E G T D A R

CCC TAC TGC AAG GTG TTG CTG CTG CAC GAG GTA CAT GCC AAC GAC

P Y C K V L L L H E V H A N D

ACA GGC AGC TAC GTC TGC TAC TAC AAG TAC ATC AAG GCA CGC ATC

T G S Y V C Y Y K Y I K A R I

GAG GGC ACC ACG GCC GCC AGC TCC TAC GTG TTC GTG AGA GGA AGG

E G T T A A S S Y V F V R G R

ACG CCA TGT GGG TGC CCT GTC TGG TGT CCA TCC CCG GCC TCA ATG

T P C G C P V W C P S P A S M

TCA CGC TGC GCT CGC AAA GCT CGG TGC TGT GGC CAG ACG GGC AGG

S R C A R K A R C C G Q T G R

AGG TGG TGT GGG ATG ACC GGC GGG GCA TGC TCG TGT CCA CGC CAC

R W C G M T G G A C S C P R H

TGC TGC ACG ATG CCC TGT ACC TGC AGT GCG AGA CCA CCT GGG GAG

C C T M P C T C S A R P P G E

ACC AGG ACT TCC TTT CCA ACC CCT TCC TGG TGC ACA TCA CAG GCA

T R T S F P T P S W C T S Q A

ACG AGC TCT ATG ACA TCC AGC TGT TGC CCA GGA AGT CGC TGG AGC

T S S M T S S C C P G S R W S

TGC TGG TAG

C W *

>EU826567,020H07,splice variant of MET,MET-877

ATG AAG GCC CCC GCT GTG CTT GCA CCT GGC ATC CTC GTG CTC CTG

M K A P A V L A P G I L V L L

TTT ACC TTG GTG CAG AGG AGC AAT GGG GAG TGT AAA GAG GCA CTA

F T L V Q R S N G E C K E A L

GCA AAG TCC GAG ATG AAT GTG AAT ATG AAG TAT CAG CTT CCC AAC

A K S E M N V N M K Y Q L P N

TTC ACC GCG GAA ACA CCC ATC CAG AAT GTC ATT CTA CAT GAG CAT

F T A E T P I Q N V I L H E H

CAC ATT TTC CTT GGT GCC ACT AAC TAC ATT TAT GTT TTA AAT GAG

H I F L G A T N Y I Y V L N E

GAA GAC CTT CAG AAG GTT GCT GAG TAC AAG ACT GGG CCT GTG CTG

E D L Q K V A E Y K T G P V L

GAA CAC CCA GAT TGT TTC CCA TGT CAG GAC TGC AGC AGC AAA GCC

E H P D C F P C Q D C S S K A

AAT TTA TCA GGA GGT GTT TGG AAA GAT AAC ATC AAC ATG GCT CTA

N L S G G V W K D N I N M A L

GTT GTC GAC ACC TAC TAT GAT GAT CAA CTC ATT AGC TGT GGC AGC

V V D T Y Y D D Q L I S C G S

GTC AAC AGA GGG ACC TGC CAG CGA CAT GTC TTT CCC CGC AAT CAT

V N R G T C Q R H V F P R N H

ACT GCT GAC ATA CAG TCG GAG GTT CAC TGC ATA TTC TCC CCA CAG

T A D I Q S E V H C I F S P Q

ATA GAA GAG CCC AGC CAG TGT CCT GAC TGT GTG GTG AGC GCC CTG

I E E P S Q C P D C V V S A L

GGA GCC AAA GTC CTT TCA TCT GTA AAG GAC CGG TTC ACC AAC TTC

G A K V L S S V K D R F T N F

TTT GTA GGC AAT ACC ATA AAT TCT TCT TAT TTC CCA GAT CAT CCA

F V G N T I N S S Y F P D H P

TTG CAT TCG ATA TCA GTG AGA AGG CTA AAG GAA ACG AAA GAT GGT

L H S I S V R R L K E T K D G

TTT ATG TTT TTG ACG GAC CAG TCC TAC ATT GAT GTT TTA CCT GAG

F M F L T D Q S Y I D V L P E

TTC AGA GAT TCT TAC CCC ATT AAG TAT GTC CAT GCC TTT GAA AGC

F R D S Y P I K Y V H A F E S

AAC AAT TTT ATT TAC TTC TTG ACG GTC CAA AGG GAA ACT CTA GAT

N N F I Y F L T V Q R E T L D

GCT CAG ACT TTT CAC ACA AGA ATA ATC AGG TTC TGT TCC ATA AAC

A Q T F H T R I I R F C S I N

TCT GGA TTG CAT TCC TAC ATG GAA ATG CCT CTG GAG TGT ATT CTC

S G L H S Y M E M P L E C I L

ACA GAA AAG AGA AAA AAG AGA TCC ACA AAG AAG GAA GTG TTT AAT

T E K R K K R S T K K E V F N

ATA CTT CAG GCT GCG TAT GTC AGC AAG CCT GGG GCC CAG CTT GCT

I L Q A A Y V S K P G A Q L A

AGA CAA ATA GGA GCC AGC CCG AAT GAT GAC ATT CTT TTC GGA GTG

R Q I G A S P N D D I L F G V

TTC GCA CAA AGC AAG CCA GAT TCT GCC GAA CCA ATG GAT CGA TCT

F A Q S K P D S A E P M D R S

GCC ATG TGT GCA TTC CCT ATC AAA TAT GTC AAC GAC TTC TTC AAC

A M C A F P I K Y V N D F F N

AAG ATC GTC AAC AAA AAC AAT GTG AGA TGT CTC CAG CAT TTT TAC

K I V N K N N V R C L Q H F Y

GGA CCC AAT CAT GAG CAC TGC TTT AAT AGG ACA CTT CTG AGA AAT

G P N H E H C F N R T L L R N

TCA TCA GGC TGT GAA GCG CGC CGT GAT GAA TAT CGA ACA GAG TTC

S S G C E A R R D E Y R T E F

ACC ACA GCT TTG CAG CGC GTT GAC TTA TTC ATG GGT CAA TTC AGC

T T A L Q R V D L F M G Q F S

GAA GTC CTC TTA ACA TCT ATA TCC ACC TTC ATT AAA GGA GAC CTC

E V L L T S I S T F I K G D L

ACC ATA GCT AAT CTT GGG ACA TCA GAG GGT CGC TTC ATG CAG GTT

T I A N L G T S E G R F M Q V

GTG GTT TCT CGA TCA GGA CCA TCA ACC CCT CAT GTG AAT TTT CTC

V V S R S G P S T P H V N F L

CTG GAC TCC CAT CCA GTG TCT CCA GAA GTG ATT GTG GAG CAT ACA

L D S H P V S P E V I V E H T

TTA AAC CAA AAT GGC TAC ACA CTG GTT ATC ACT GGG AAG AAG ATC

L N Q N G Y T L V I T G K K I

ACG AAG ATC CCA TTG AAT GGC TTG GGC TGC AGA CAT TTC CAG TCC

T K I P L N G L G C R H F Q S

TGC AGT CAA TGC CTC TCT GCC CCA CCC TTT GTT CAG TGT GGC TGG

C S Q C L S A P P F V Q C G W

TGC CAC GAC AAA TGT GTG CGA TCG GAG GAA TGC CTG AGC GGG ACA

C H D K C V R S E E C L S G T

TGG ACT CAA CAG ATC TGT CTG CCT GCA ATC TAC AAG GTT TTC CCA

W T Q Q I C L P A I Y K V F P

AAT AGT GCA CCC CTT GAA GGA GGG ACA AGG CTG ACC ATA TGT GGC

N S A P L E G G T R L T I C G

TGG GAC TTT GGA TTT CGG AGG AAT AAT AAA TTT GAT TTA AAG AAA

W D F G F R R N N K F D L K K

ACT AGA GTT CTC CTT GGA AAT GAG AGC TGC ACC TTG ACT TTA AGT

T R V L L G N E S C T L T L S

GAG AGC ACG ATG AAT ACA TTG AAA TGC ACA GTT GGT CCT GCC ATG

E S T M N T L K C T V G P A M

AAT AAG CAT TTC AAT ATG TCC ATA ATT ATT TCA AAT GGC CAC GGG

N K H F N M S I I I S N G H G

ACA ACA CAA TAC AGT ACA TTC TCC TAT GTG GAT CCT GTA ATA ACA

T T Q Y S T F S Y V D P V I T

AGT ATT TCG CCG AAA TAC GGT CCT ATG GCT GGT GGC ACT TTA CTT

S I S P K Y G P M A G G T L L

ACT TTA ACT GGA AAT TAC CTA AAC AGT GGG AAT TCT AGA CAC ATT

T L T G N Y L N S G N S R H I

TCA ATT GGT GGA AAA ACA TGT ACT TTA AAA AGT GTG TCA AAC AGT

S I G G K T C T L K S V S N S

ATT CTT GAA TGT TAT ACC CCA GCC CAA ACC ATT TCA ACT GAG TTT

I L E C Y T P A Q T I S T E F

GCT GTT AAA TTG AAA ATT GAC TTA GCC AAC CGA GAG ACA AGC ATC

A V K L K I D L A N R E T S I

TTC AGT TAC CGT GAA GAT CCC ATT GTC TAT GAA ATT CAT CCA ACC

F S Y R E D P I V Y E I H P T

AAA TCT TTT ATT AGT GGT GGG AGC ACA ATA ACA GGT GTT GGG AAA

K S F I S G G S T I T G V G K

AAC CTG AAT TCA GTT AGT GTC CCG AGA ATG GTC ATA AAT GTG CAT

N L N S V S V P R M V I N V H

GAA GCG GGA AGG AAC TTT ACA GTG GCA TGT CAA CAT CGC TCT AAT

E A G R N F T V A C Q H R S N

TCA GAG ATA ATC TGT TGT ACC ACT CCT TCC CTG CAA CAG CTG AAT

S E I I C C T T P S L Q Q L N

CTG CAA CTC CCC CTG AAA ACC AAA GCC TTT TTC ATG TTA GAT GGG

L Q L P L K T K A F F M L D G

ATC CTT TCC AAA TAC TTT GAT CTC ATT TAT GTA CAT AAT CCT GTG

I L S K Y F D L I Y V H N P V

TTT AAG CCT TTT GAA AAG CCA GTG ATG ATC TCA ATG GGC AAT GAA

F K P F E K P V M I S M G N E

AAT GTA CTG GAA ATT AAG GTA AGA AAT GCT TTA AAC ACT GTC TTA

N V L E I K V R N A L N T V L

AAT CAT CAG CTC AAA CTT AAT TGA

N H Q L K L N *

>EU826568,020H06,splice variant of MET,MET-823

ATG AAG GCC CCC GCT GTG CTT GCA CCT GGC ATC CTC GTG CTC CTG

M K A P A V L A P G I L V L L

TTT ACC TTG GTG CAG AGG AGC AAT GGG GAG TGT AAA GAG GCA CTA

F T L V Q R S N G E C K E A L

GCA AAG TCC GAG ATG AAT GTG AAT ACG AAG TAT CAG CTT CCC AAC

A K S E M N V N T K Y Q L P N

TTC ACC GCG GAA ACA CCC ATC CAG AAT GTC ATT CTA CAT GAG CAT

F T A E T P I Q N V I L H E H

CAC ATT TTC CTT GGT GCC ACT AAC TAC ATT TAT GTT TTA AAT GAG

H I F L G A T N Y I Y V L N E

GAA GAC CTT CAG AAG GTT GCT GAG TAC AAG ACT GGG CCT GTG CTG

E D L Q K V A E Y K T G P V L

GAA CAC CCA GAT TGT TTC CCA TGT CAG GAC TGC AGC AGC AAA GCC

E H P D C F P C Q D C S S K A

AAT TTA TCA GGA GGT GTT TGG AAA GAT AAC ATC AAC ATG GCT CTA

N L S G G V W K D N I N M A L

GTT GTC GAC ACC TAC TAT GAT GAT CAA CTC ATT AGC TGT GGC AGC

V V D T Y Y D D Q L I S C G S

GTC AAC AGA GGG ACC TGC CAG CGA CAT GTC TTT CCC CAC AAT CAT

V N R G T C Q R H V F P H N H

ACT GCT GAC ATA CAG TCG GAG GTT CAC TGC ATA TTC TCC CCA CAG

T A D I Q S E V H C I F S P Q

ATA GAA GAG CCC AGC CAG TGT CCT GAC TGT GTG GTG AGC GCC CTG

I E E P S Q C P D C V V S A L

GGA GCC AAA GTC CTT TCA TCT GTA AAG GAC CGG TTC ATC AAC TTC

G A K V L S S V K D R F I N F

TTT GTA GGC AAT ACC ATA AAT TCT TCT TAT TTC CCA GAT CAT CCA

F V G N T I N S S Y F P D H P

TTG CAT TCG ATA TCA GTG AGA AGG CTA AAG GAA ACG AAA GAT GGT

L H S I S V R R L K E T K D G

TTT ATG TTT TTG ACG GAC CAG TCC TAC ATT GAT GTT TTA CCT GAG

F M F L T D Q S Y I D V L P E

TTC AGA GAT TCT TAC CCC ATT AAG TAT GTC CAT GCC TTT GAA AGC

F R D S Y P I K Y V H A F E S

AAC AAT TTT ATT TAC TTC TTG ACG GTC CAA AGG GAA ACT CTA GAT

N N F I Y F L T V Q R E T L D

GCT CAG ACT TTT CAC ACA AGA ATA ATC AGG TTC TGT TCC ATA AAC

A Q T F H T R I I R F C S I N

TCT GGA TTG CAT TCC TAC ATG GAA ATG CCT CTG GAG TGT ATT CTC

S G L H S Y M E M P L E C I L

ACA GAA AAG AGA AAA AAG AGA TCC ACA AAG AAG GAA GTG TTT AAT

T E K R K K R S T K K E V F N

ATA CTT CAG GCT GCG TAT GTC AGC AAG CCT GGG GCC CAG CTT GCT

I L Q A A Y V S K P G A Q L A

AGA CAA ATA GGA GCC AGC CTG AAT GAT GAC ATT CTT TTC GGG GTG

R Q I G A S L N D D I L F G V

TTC GCA CAA AGC AAG CCA GAT TCT GCC GAA CCA ATG GAT CGA TCT

F A Q S K P D S A E P M D R S

GCC ATG TGT GCA TTC CCT ATC AAA TAT GTC AAC GAC TTC TTC AAC

A M C A F P I K Y V N D F F N

AAG ATC GTC AAC AAA AAC AAT GTG AGA TGT CTC CAG CAT TTT TAC

K I V N K N N V R C L Q H F Y

GGA CCC AAT CAT GAG CAC TGC TTT AAT AGG ACA CTT CTG AGA AAT

G P N H E H C F N R T L L R N

TCA TCA GGC TGT GAA GCG CGC CGT GAT GAA TAT CGA ACA GAG TTT

S S G C E A R R D E Y R T E F

ACC ACA GCT TTG CAG CGC GTT GAC TTA TTC ATG GGT CAA TTC AGC

T T A L Q R V D L F M G Q F S

GAA GTC CTC TTA ACA TCT ATA TCC ACC TTC ATT AAA GGA GAC CTC

E V L L T S I S T F I K G D L

ACC ATA GCT AAT CTT GGG ACA TCA GAG GGT CGC TTC ATG CAG GTT

T I A N L G T S E G R F M Q V

GTG GTT TCT CGA TCA GGA CCA TCA ACC CCT CAT GTG AAT TTT CTC

V V S R S G P S T P H V N F L

CTG GAC TCC CAT CCA GTG TCT CCA GAA GTG ATT GTG GAG CAT ACA

L D S H P V S P E V I V E H T

TTA AAC CAA AAT GGC TAC ACA CTG GTT ATC ACT GGG AAG AAG ATC

L N Q N G Y T L V I T G K K I

ACG AAG ATC CCA TTG AAT GGC TTG GGC TGC AGA CAT TTC CAG TCC

T K I P L N G L G C R H F Q S

TGC AGT CAA TGC CTC TCT GCC CCA CCC TTT GTT CAG TGT GGC TGG

C S Q C L S A P P F V Q C G W

TGC CAC GAC AAA TGT GTG CGA TCG GAG GAA TGC CTG AGC GGG ACA

C H D K C V R S E E C L S G T

TGG ACT CAA CAG ATC TGT CTG CCT GCA ATC TAC AAG GTT TTC CCA

W T Q Q I C L P A I Y K V F P

AAT AGT GCA CCC CTT GAA GGA GGG ACA AGG CTG ACC ATA TGT GGC

N S A P L E G G T R L T I C G

TGG GAC TTT GGA TTT CGG AGG AAT AAT AAA TTT GAT TTA AAG AAA

W D F G F R R N N K F D L K K

ACT AGA GTT CTC CTT GGA AAT GAG AGC TGC ACC TTG ACT TTA AGT

T R V L L G N E S C T L T L S

GAG AGC ACG ATG AAT ACA TTG AAA TGC ACA GTT GGT CCT GCC ATG

E S T M N T L K C T V G P A M

AAT AAG CAT TTC AAT ATG TCC ATA ATT ATT TCA AAT GGC CAC GGG

N K H F N M S I I I S N G H G

ACA ACA CAA TAC AGT ACA TTC TCC TAT GTG GAT CCT GTA ATA ACA

T T Q Y S T F S Y V D P V I T

AGT ATT TCG CCG AAA TAC GGT CCT ATG GCT GGT GGC ACT TTA CTT

S I S P K Y G P M A G G T L L

ACT TTA ACT GGA AAT TAC CTA AAC AGT GGG AAT TCT AGA CAC ATT

T L T G N Y L N S G N S R H I

TCA ATT GGT GGA AAA ACA TGT ACT TTA AAA AGT GTG TCA AAC AGT

S I G G K T C T L K S V S N S

ATT CTT GAA TGT TAT ACC CCA GCC CAA ACC ATT TCA ACT GAG TTT

I L E C Y T P A Q T I S T E F

GCT GTT AAA TTG AAA ATT GAC TTA ACC AAC CGA GAG ACA AGC ATC

A V K L K I D L T N R E T S I

TTC AGT TAC CGT GAA GAT CCC ATT GTC TAT GAA ATT CAT CCA ACC

F S Y R E D P I V Y E I H P T

AAA TCT TTT ATT AGT GGT GGG AGC ACA ATA ACA GGT GTT GGG AAA

K S F I S G G S T I T G V G K

AAC CTG AAT TCA GTT AGT GTC CCG AGA ATG GTC ATA AAT GTG CAT

N L N S V S V P R M V I N V H

GAA GCA GGA AGG AAC TTT ACA GTG GTA AGT CCT TTG AGC AAT GGT

E A G R N F T V V S P L S N G

TCT ACT CAG AGC TCT GCA TCT TTG CCT CTA ACC ATG TGG CTT TCA

S T Q S S A S L P L T M W L S

TGG TAC CTG AGA CAT CTC AGT TTC GCC TTT AAG GTT TGC TAG

W Y L R H L S F A F K V C *

>EU826569,020H03,splice variant of MET,MET-755

ATG AAG GCC CCC GCT GTG CTT GCA CCT GGC ATC CTC GTG CTC CTG

M K A P A V L A P G I L V L L

TTT ACC TTG GTG CAG AGG AGC AAT GGG GAG TGT AAA GAG GCA CTA

F T L V Q R S N G E C K E A L

GCA AAG TCC GAG ATG AAT GTG AAT ATG AAG TAT CAG CTT CCC AAC

A K S E M N V N M K Y Q L P N

TTC ACC GCG GAA ACA CCC ATC CAG AAT GTC ATT CTA CAT GAG CAT

F T A E T P I Q N V I L H E H

CAC ATT TTC CTT GGT GCC ACT AAC TAC ATT TAT GTT TTA AAT GAG

H I F L G A T N Y I Y V L N E

GAA GAC CTT CAG AAG GTT GCT GAG TAC AAG ACT GGG CCT GTG CTG

E D L Q K V A E Y K T G P V L

GAA CAC CCA GAT TGT TTC CCA TGT CAG GAC TGC AGC AGC AAA GCC

E H P D C F P C Q D C S S K A

AAT TTA TCA GGA GGT GTT TGG AAA GAT AAC ATC AAC ATG GCT CTA

N L S G G V W K D N I N M A L

GTT GTC GAC ACC TAC TAT GAT GAT CAA CTC ATT AGC TGT GGC AGC

V V D T Y Y D D Q L I S C G S

GTC AAC AGA GGG ACC TGC CAG CGA CAT GTC TTT CCC CAC AAT CAT

V N R G T C Q R H V F P H N H

ACT GCT GAC ATA CAG TCG GAG GTT CAC TGC ATA TTC TCC CCA CAG

T A D I Q S E V H C I F S P Q

ATA GAA GAG CCC AGC CAG TGT CCT GAC TGT GTG GTG AGC GCC CTG

I E E P S Q C P D C V V S A L

GGA GCC AAA GTC CTT TCA TCT GTA AAG GAC CGG TTC ATC AAC TTC

G A K V L S S V K D R F I N F

TTT GTA GGC AAT ACC ATA AAT TCT TCT TAT TTC CCA GAT CAT CCA

F V G N T I N S S Y F P D H P

TTG CAT TCG ATA TCA GTG AGA AGG CTA AAG GAA ACG AAA GAT GGT

L H S I S V R R L K E T K D G

TTT ATG TTT TTG ACG GAC CAG TCC TAC ATT GAT GTT TTA CCT GAG

F M F L T D Q S Y I D V L P E

TTC AGA GAT TCT TAC CCC ATT AAG TAT GTC CAT GCC TTT GAA AGC

F R D S Y P I K Y V H A F E S

AAC AAT TTT ATT TAC TTC TTG ACG GTC CAA AGG GAA ACT CTA GAT

N N F I Y F L T V Q R E T L D

GCT CAG ACT TTT CAC ACA AGA ATA ATC AGG TTC TGT TCC ATA AAC

A Q T F H T R I I R F C S I N

TCT GGA TTG CAT TCC TAC ATG GAA ATG CCT CTG GAG TGT ATT CTC

S G L H S Y M E M P L E C I L

ACA GAA AAG AGA AAA AAG AGA TCC ACA AAG AAG GAA GTG TTT AAT

T E K R K K R S T K K E V F N

ATA CTT CAG GCT GCG TAT GTC AGC AAG CCT GGG GCC CAG CTT GCT

I L Q A A Y V S K P G A Q L A

AGA CAA ATA GGA GCC AGC CTG AAT GAT GAC ATT CTT TTC GGG GTG

R Q I G A S L N D D I L F G V

TTC GCA CAA AGC AAG CCA GAT TCT GCC GAA CCA ATG GAT CGA TCT

F A Q S K P D S A E P M D R S

GCC ATG TGT GCA TTC CCT ATC AAA TAT GTC AAC GAC TTC TTC AAC

A M C A F P I K Y V N D F F N

AAG ATC GTC AAC AAA AAC GAT GTG AGA TGT CTC CAG CAT TTT TAC

K I V N K N D V R C L Q H F Y

GGA CCC AAT CAT GAG CAC TGC TTT AAT GGG ACA CTT CTG AGA AAT

G P N H E H C F N G T L L R N

TCA TCA GGC TGT GAA GCG CGC CGT GAT GAA TAT CGA ACA GAG TTT

S S G C E A R R D E Y R T E F

ACC ACA GCT TTG CAG CGC GTT GAC TTA TTC ATG GGT CAA TTC AGC

T T A L Q R V D L F M G Q F S

GAA GTC CTC TTA ACA TCT ATA TCC ACC TTC ATT AAA GGA GAC CTC

E V L L T S I S T F I K G D L

ACC ATA GCT AAT CTT GGG ACA TCA GAG GGT CGC TTC ATG CAG GTT

T I A N L G T S E G R F M Q V

GTG GTT TCT CGA TCA GGA CCA TCA ACC CCT CGT GTG AAT TTT CTC

V V S R S G P S T P R V N F L

CTG GAC TCC CAT CCA GTG TCT CCA GAA GTG ATT GTG GAG CAT ACA

L D S H P V S P E V I V E H T

TTA AAC CAA AAT GGC TAC ACA CTG GTT ATC ACT GGG AAG AAG ATC

L N Q N G Y T L V I T G K K I

ACG AAG ATC CCA TTG AAT GGT TTG GGC TGC AGA CAT TTC CAG TCC

T K I P L N G L G C R H F Q S

TGC AGT CAA TGC CTC TCT GCC CCA CCC TTT GTT CAG TGT GGC TGG

C S Q C L S A P P F V Q C G W

TGC TAC GAC AAA TGT GTG CGA TCG GAG GAA TGC CTG AGC GGG ACA

C Y D K C V R S E E C L S G T

TGG ACT CAA CAG ATC TGT CTG CCT GCA ATC TAC AAG GTT TTC CCA

W T Q Q I C L P A I Y K V F P

AAT AGT GCA CCC CTT GAA GGA GGG ACA AGG CTG ACC ATA TGT GGC

N S A P L E G G T R L T I C G

TGG GAC TTT GGA TTT CGG AGG AAT AAT AAA TTT GAT TTA AAG AAA

W D F G F R R N N K F D L K K

ACT AGA GTT CTC CTT GGA AAT GAG AGC TGC ACC TTG ACT TTA AGT

T R V L L G N E S C T L T L S

GAG AGC ACG ATG AAT ACA TCG AAA TGC ACA GTT GGT CCT GCC ATG

E S T M N T S K C T V G P A M

AAT AAG CAT TTC AAT ATG TCC ATA ATT ATT TCA AAT GGC CAC GGG

N K H F N M S I I I S N G H G

ACA ACA CAA TAC AGT ACA TTC TCC TAT GTG GAT CCT GTA ATA ACA

T T Q Y S T F S Y V D P V I T

AGT ATT TCG CCG AAA TAC GGT CCT ATG GCT GGT GGC ACT TTA CTT

S I S P K Y G P M A G G T L L

ACT TTA ACT GGA AAT TAC CTA AAC AGT GGG AAT TCT AGA CAC ATT

T L T G N Y L N S G N S R H I

TCA ATT GGT GGG AAA ACA TGT ACT TTA AAA AGT GTG TCA AAC AGT

S I G G K T C T L K S V S N S

ATT CTT GAA TGT TAT ACC CCA GCC CAA ACC ATT TCA ACT GAG TTT

I L E C Y T P A Q T I S T E F

GCT GTT AAA TTG AAA ATT GAC TTA GCC AAC CGA GAG ACA AGC ATC

A V K L K I D L A N R E T S I

TTC AGT TAC CGT GAA GAT CCC ATT GTC TAT GTA ATT CAT CCA ACC

F S Y R E D P I V Y V I H P T

AAA TCT TTT ATT AGG TAA

K S F I R *

>EU826570,020H08,splice variant of MET,MET-764

ATG AAG GCC CCC GCT GTG CTT GCA CCT GGC ATC CTC GTG CTC CTG

M K A P A V L A P G I L V L L

TTT ACC TTG GTG CAG AGG AGC AAT GGG GAG TGT AAA GAG GCA CTA

F T L V Q R S N G E C K E A L

GCA AAG TCC GAG ATG AAT GTG AAT ATG AAG TAT CAG CTT CCC AAC

A K S E M N V N M K Y Q L P N

TTC ACC GCG GAA ACA CCC ATC CAG AAT GTC ATT CTA CAT GAG CAT

F T A E T P I Q N V I L H E H

CAC ATT TTC CTT GGT GCC ACT AAC TAC ATT TAT GTT TTA AAT GAG

H I F L G A T N Y I Y V L N E

GAA GAC CTT CAG AAG GTT GCT GAG TAC AAG ACT GGG CCT GTG CTG

E D L Q K V A E Y K T G P V L

GAA CAC CCA GAT TGT TTC CCA TGT CAG GAC TGC AGC AGC AAA GCC

E H P D C F P C Q D C S S K A

AAT TTA TCA GGA GGT GTT TGG AAA GAT AAC ATC AAC ATG GCT CTA

N L S G G V W K D N I N M A L

GTT GTC GAC ACC TAC TAT GAT GAT CAA CTC ATT AGC TGT GGC AGC

V V D T Y Y D D Q L I S C G S

GTC AAC AGA GGG ACC TGC CAG CGA CAT GTC TTT CCC CAC AAT CAT

V N R G T C Q R H V F P H N H

ACT GCT GAC ATA CAG TCG GAG GTT CAC TGC ATA TTC TCC CCA CAG

T A D I Q S E V H C I F S P Q

ATA GAA GAG CCC AGC CAG TGT CCT GAC TGT GTG GTG AGC GCC CTG

I E E P S Q C P D C V V S A L

GGA GCC AAA GTC CTT TCA TCT GTA AAG GAC CGG TTC ATC AAC TTC

G A K V L S S V K D R F I N F

TTT GTA GGC AAT ACC ATA AAT TCT TCT TAT TTC CCA GAT CAT CCA

F V G N T I N S S Y F P D H P

TTG CAT TCG ATA TCA GTG AGA AGG CTA AAG GAA ACG AAA GAT GGT

L H S I S V R R L K E T K D G

TTT ATG TTT TTG ACG GAC CAG TCC TAC ATT GAT GCT TTA CCT GAG

F M F L T D Q S Y I D A L P E

TTC AGA GAT TCT TAC CCC ATT AAG TAT GTC CAT GCC TTT GAA AGC

F R D S Y P I K Y V H A F E S

AAC AAT TTT ATT TAC TTC TTG ACG GTC CAA AGG GAA ACT CTA GAT

N N F I Y F L T V Q R E T L D

GCT CAG ACT TTT CAC ACA AGA ATA ATC AGG TTC TGT TCC ATA AAC

A Q T F H T R I I R F C S I N

TCT GGA TTG CAT TCC TAC ATG GAA ATG CCT CTG GAG TGT ATT CTC

S G L H S Y M E M P L E C I L

ACA GAA AAG AGA AAA AAG AGA TCC ACA AAG AAG GAA GTG TTT AAT

T E K R K K R S T K K E V F N

ATA CTT CAG GCT GCG TAT GTC AGC AAG CCT GGG GCC CAG CTT GCT

I L Q A A Y V S K P G A Q L A

AGA CAA ATA GGA GCC AGC CTG AAT GAT GAC ATT CTT TTC GGG GTG

R Q I G A S L N D D I L F G V

TTC GCA CAA AGC AAG CCA GAT TCT GCC GAA CCA ATG GAT CGA TCT

F A Q S K P D S A E P M D R S

GCC ATG TGT GCA TTC CCT ATC AAA TAT GTC AAC GAC TTC TTC AAC

A M C A F P I K Y V N D F F N

AAG ATC GTC AAC AAA AAC AAT GTG AGA TGT CTC CAG CAT TTT TAC

K I V N K N N V R C L Q H F Y

GGA CCC AAT CAT GAG CAC TGC TTT AAT AGG ACA CTT CTG AGA AAT

G P N H E H C F N R T L L R N

TCA TCA GGC TGT GAA GCG CGC CGT GAT GAA TAT CGA ACA GAG TTT

S S G C E A R R D E Y R T E F

ACC ACA GCT TTG CAG CGC GTT GAC TTA TTC ATG GGT CAA TTC AGC

T T A L Q R V D L F M G Q F S

GAA GTC CTC TTA ACA TCT ATA TCC ACC TTC ATT AAA GGA GAC CTC

E V L L T S I S T F I K G D L

ACC ATA GCT AAT CTT GGG ACA TCA GAG GGT CGC TTC ATG CAG GTT

T I A N L G T S E G R F M Q V

GTG GTT TCT CGA TCA GGA CCA TCA ACC CCT CAT GTG AAT TTT CTC

V V S R S G P S T P H V N F L

CTG GAC TCC CAT CCA GTG TCT CCA GAA GTG ATT GTG GAG CAT ACA

L D S H P V S P E V I V E H T

TTA AAC CAA AAT GGC TAC ACA CTG GTT ATC ACT GGG AGG AAG ATC

L N Q N G Y T L V I T G R K I

ACG AAG ATC CCA TTG AAT GGC TTG GGC TGC AGA CAT TTC CAG TCC

T K I P L N G L G C R H F Q S

TGC AGT CAA TGC CTC TCT GCC CCA CCC TTT GTT CAG TGT GGC TGG

C S Q C L S A P P F V Q C G W

TGC CAC GAC AAA TGT GTG CGA TCG GAG GAA TGC CTG AGC GGG ACA

C H D K C V R S E E C L S G T

TGG ACT CAG CAG ATC TGT CTG CCT GCA ATC TAC AAG GTT TTC CCA

W T Q Q I C L P A I Y K V F P

AAT AGT GCA CCC CTT GAA GGA GGG ACA AGG CTG ACC ATA TGT GGC

N S A P L E G G T R L T I C G

TGG GAC TTT GGA TTT CGG AGG AAT AAT AAA TTT GAT TTA AAG AAA

W D F G F R R N N K F D L K K

ACT AGA GTT CTC CTT GGA AAT GAG AGC TGC ACC TTG ACT TTA AGT

T R V L L G N E S C T L T L S

GAG AGC ACG ATG AAT ACA TTG AAA TGC ACA GTT GGT CCT GCC ATG

E S T M N T L K C T V G P A M

AAT AAG CAT TTC AAT ATG TCC ATA ATT ATT TCA AAT GGC CAC GGG

N K H F N M S I I I S N G H G

ACA ACA CAA TAC AGT ACA TTC TCC TAT GTG GAT CCT GTA ATA ACA

T T Q Y S T F S Y V D P V I T

AGT ATT TCG CCG AAA TAC GGT CCT ATG GCT GGT GGC ACT TTA CTT

S I S P K Y G P M A G G T L L

ACT TTA ACT GGA AAT TAC CTA AAC AGT GGG AAT TCT AGA CAC ATT

T L T G N Y L N S G N S R H I

TCA ATT GGT GGA AAA ACG TGT ACT TTA AAA AGT GTG TCA AAC AGT

S I G G K T C T L K S V S N S

ATT CTT GAA TGT TAT ACC CCA GCC CAA ACC ATT TCA ACT GAG TCT

I L E C Y T P A Q T I S T E S

GCT GTT AAA TTG AAA ATT GAC TTA GCC AAC CGA GAG ACA AGC ATC

A V K L K I D L A N R E T S I

TTC AGT TAC CGT GAA GAT CCC ATT GTC TAT GAA ATT CAT CCA ACC

F S Y R E D P I V Y E I H P T

AAA TCT TTT ATT AGG CAT GTC AAC ATC GCT CTA ATT CAG AGA TAA

K S F I R H V N I A L I Q R *

>EU826571,020G07,splice variant of MET,MET-661

ATG AAG GCC CCC GCT GTG CTT GCA CCT GGC ATC CTC GTG CTC CTG

M K A P A V L A P G I L V L L

TTT ACC TTG GTG CAG AGG AGC AAT GGG GAG TGT AAA GAG GCA CTA

F T L V Q R S N G E C K E A L

GCA AAG TCC GAG ATG AAT GTG AAT ATG AAG TAT CAG CTT CCC AAC

A K S E M N V N M K Y Q L P N

TTC ACC GCG GAA ACA CCC ATC CAG AAT GTC ATT CTA CAT GAG CAT

F T A E T P I Q N V I L H E H

CAC ATT TTC CTT GGT GCC ACT AAC TAC ATT TAT GTT TTA AAT GAG

H I F L G A T N Y I Y V L N E

GAA GAC CTT CAG AAG GTT GCT GAG TAC AAG ACT GGG CCT GTG CTG

E D L Q K V A E Y K T G P V L

GAA CAC CCA GAT TGT TTC CCA TGT CAG GAC TGC AGC AGC AAA GCC

E H P D C F P C Q D C S S K A

AAT TTA TCA GGA GGT GTT TGG AAA GAT AAC ATC AAC ATG GCT CTA

N L S G G V W K D N I N M A L

GTT GTC GAC ACC TAC TAT GAT GAT CAA CTC ATT AGC TGT GGC AGC

V V D T Y Y D D Q L I S C G S

GTC AAC AGA GGG ACC TGC CAG CGA CAT GTC TTT CCC CAC AAT CAT

V N R G T C Q R H V F P H N H

ACT GCT GAC ATA CAG TCG GAG GTT CAC TGC ATA TTC TCC CCA CAG

T A D I Q S E V H C I F S P Q

ATA GAA GAG CCC AGC CAG TGT CCT GAC TGT GTG GTG AGC GCC CTG

I E E P S Q C P D C V V S A L

GGA GCC AAA GTC CTT TCA TCT GTA AAG GAC CGG TTC ATC AAC TTC

G A K V L S S V K D R F I N F

TTT GTA GGC AAT ACC ATA AAT TCT TCT TAT TTC CCA GAT CAT CCA

F V G N T I N S S Y F P D H P

TTG CAT TCG ATA TCA GTG AGA AGG CTA AAG GAA ACG AAA GAT GGT

L H S I S V R R L K E T K D G

TTT ATG TTT TTG ACG GAC CAG TCC TAC ATT GAT GTT TTA CCT GAG

F M F L T D Q S Y I D V L P E

TTC AGA GAT TCT TAC CCC ATT AAG TAT GTC CAT GCC TTT GAA AGC

F R D S Y P I K Y V H A F E S

AAC AAT TTT ATT TAC TTC TTG ACG GTC CAA AGG GAA ACT CTA GAT

N N F I Y F L T V Q R E T L D

GCT CAG ACT TTT CAC ACA AGA ATA ATC AGG TTC TGT TCC ATA AAC

A Q T F H T R I I R F C S I N

TCT GGA TTG CAT TCC TAC ATG GAA ATG CCT CTG GAG TGT ATT CTC

S G L H S Y M E M P L E C I L

ACA GAA AAG AGA AAA AAG AGA TCC ACA AAG AAG GAA GTG TTT AAT

T E K R K K R S T K K E V F N

ATA CTT CAG GCT GCG TAT GTC AGC AAG CCT GGG GCC CAG CTT GCT

I L Q A A Y V S K P G A Q L A

AGA CAA ATA GGA GCC AGC CTG AAT GAT GAC ATT CTT TTC GGG GTG

R Q I G A S L N D D I L F G V

TTC GCA CAA AGC AAG CCA GAT TCT GCC GAA CCA ATG GAT CGA TCT

F A Q S K P D S A E P M D R S

GCC ATG TGT GCA TTC CCT ATC AAA TAT GTC AAC GAC TTC TTC AAC

A M C A F P I K Y V N D F F N

AAG ATC GTC AAC AAA AAC AAT GTG AGA TGT CTC CAG CAT TTT TAC

K I V N K N N V R C L Q H F Y

GGA CCC AAT CAT GAG CAC TGC TTT AAT AGG ACA CTT CTG AGA AAT

G P N H E H C F N R T L L R N

TCA TCA GGC TGT GAA GCG CGC CGT GAT GAA TAT CGA ACA GAG TTT

S S G C E A R R D E Y R T E F

ACC ACA GCT TTG CAG CGC GTT GAC TTA TTC ATG GGT CAA TTC AGC

T T A L Q R V D L F M G Q F S

GAA GTC CTC TTA ACA TCT ATA TCC ACC TTC ATT AAA GGA GAC CTC

E V L L T S I S T F I K G D L

ACC ATA GCT AAT CTT GGG ACA TCA GAG GGT CGC TTC ATG CAG GTT

T I A N L G T S E G R F M Q V

GTG GTT TCT CGA TCA GGA CCA TCA ACC CCT CAT GTG AAT TTT CTC

V V S R S G P S T P H V N F L

CTG GAC TCC CAT CCA GTG TCT CCA GAA GTG ATT GTG GAG CAT ACA

L D S H P V S P E V I V E H T

TTA AAC CAA AAT GGC TAC ACA CTG GTT ATC ACT GGG AAG AAG ATC

L N Q N G Y T L V I T G K K I

GCG AAG ATC CCA TTG AAT GGC TTG GGT TGC AGA CAT TTC CAG TCC

A K I P L N G L G C R H F Q S

TGC AGT CAA TGC CTC TCT GCC CCA CCC TTT GTT CAG TGT GGC TGG

C S Q C L S A P P F V Q C G W

TGC CAC GAC AAA TGT GTG CGA TCG GAG GAA TGC CTG AGC GGG ACA

C H D K C V R S E E C L S G T

TGG ACT CAA CAG ATC TGT CTG CCT GCA ATC TAC AAG GTT TTC CCA

W T Q Q I C L P A I Y K V F P

AAT AGT GCA CCC CTT GAA GGA GGG ACA AGG CTG ACC ATA TGT GGC

N S A P L E G G T R L T I C G

TGG GAC TTT GGA TTT CGG AGG AAT AAT AAA TTT GAT TTA AAG AAA

W D F G F R R N N K F D L K K

ACT AGA GTT CTC CTT GGA AAT GAG AGC TGC ACC TTG ACT TTA AGT

T R V L L G N E S C T L T L S

GAG AGC ACG ATG AAT ACA TTG AAA TGC ACA GTT GGT CCT GCC ATG

E S T M N T L K C T V G P A M

AAT AAG CAT TTC AAT ATG TCC ATA ATT ATT TCA AAT GGC CAC GGG

N K H F N M S I I I S N G H G

ACA ACA CAG TAC AGT ACA TTC TCC TAT GTG CTC CCC TCC AGG ATC

T T Q Y S T F S Y V L P S R I

CTG TAA

L *

>EU826572,020F11,splice variant of MET,MET-719

ATG AAG GCC CCC GCT GTG CTT GCA CCT GGC ATC CTC GTG CTC CTG

M K A P A V L A P G I L V L L

TTT ACC CTG GTG CAG AGG AGC AAT GGG GAG TGT AAA GAG GCA CTA

F T L V Q R S N G E C K E A L

GCA AAG TCC GAG ATG AAT GTG AAT ATG AAG TAT CAG CTT CCC AAC

A K S E M N V N M K Y Q L P N

TTC ACC GCG GAA ACA CCC ATC CAG AAT GTC ATT CTA CAT GAG CAT

F T A E T P I Q N V I L H E H

CAC ATT TTC CTT GGT GCC ACT AAC TAC ATT TAT GTT TTA AAT GAG

H I F L G A T N Y I Y V L N E

GAA GAC CTT CAG AAG GTT GCT GAG TAC AAG ACT GGG CCT GTG CTG

E D L Q K V A E Y K T G P V L

GAA CAC CCA GAT TGT TTC CCA TGT CAG GAC TGC AGC AGC AAA GCC

E H P D C F P C Q D C S S K A

AAT TTA TCA GGA GGT GTT TGG AAA GAT AAC ATC AAC ATG GCT CTA

N L S G G V W K D N I N M A L

GTT GTC GAC ACC TAC TAT GAT GAT CAA CTC ATT AGC TGT GGC AGC

V V D T Y Y D D Q L I S C G S

GTC AAC AGA GGG ACC TGC CAG CGA CAT GTC TTT CCC CAC AAT CAT

V N R G T C Q R H V F P H N H

CCT GCT GAC ATA CAG TCG GAG GTT CAC TGC ATA TTC TCC CCA CAG

P A D I Q S E V H C I F S P Q

ATA GAA GAG CCC AGC CAG TGT CCT GAC TGT GTG GTG AGC GCC CTG

I E E P S Q C P D C V V S A L

GGA GCC AAA GTC CTT TCA TCT GTA AAG GAC CGG TTC ATC AAC TTC

G A K V L S S V K D R F I N F

TTT GTA GGC AAT ACC ATA AAT TCT TCT TAT TTC CCA GAT CAT CCA

F V G N T I N S S Y F P D H P

TTG CAT TCG ATA TCA GTG AGA AGG CTA AAG GAA ACG AAA GAT GGT

L H S I S V R R L K E T K D G

TTT ATG TTT TTG ACG GAC CAG TCC TAC ATT GAT GTT TTA CCT GAG

F M F L T D Q S Y I D V L P E

TTC AGA GAT TCT TAC CCC ATT AAG TAT GTC CAT GCC TTT GAA AGC

F R D S Y P I K Y V H A F E S

AAC AAT TTT ATT TAC TTC TTG ACG GTC CAA AGG GAA ACT CTA GAT

N N F I Y F L T V Q R E T L D

GCT CAG ACT TTT CAC ACA AGA ATA ATC AGG TTC TGT TCC ATA AAC

A Q T F H T R I I R F C S I N

TCT GGA TTG CAT TCC TAC ATG GAA ATG CCT CTG GAG TGT ATT CTC

S G L H S Y M E M P L E C I L

ACA GAA AAG AGA AAA AAG AGA TCC ACA AAG AAG GAA GTG TTT AAT

T E K R K K R S T K K E V F N

ATA CTT CAG GCT GCG TAT GTT AGC AAG CCT GGG GCC CAG CTT GCT

I L Q A A Y V S K P G A Q L A

AGA CAA ATA GGA GCC AGC CTG AAT GAT GAC ATT CTT TTC GGG GTG

R Q I G A S L N D D I L F G V

TTC GCA CAA AGC AAG CCA GAT TCT GCC GAA CCA ATG GAT CGA TCT

F A Q S K P D S A E P M D R S

GCC ATG TGT GCA TTC CCT ATC AAA TAT GTC AAC GAC TTC TTC AAC

A M C A F P I K Y V N D F F N

AAG ATC GTC AAC AAA AAC AAT GTG AGA TGT CTC CAG CAT TTT TAC

K I V N K N N V R C L Q H F Y

GGA CCC AAT CAT GAG CAC TGC TTT AAT AGG ACA CTT CTG AGA AAT

G P N H E H C F N R T L L R N

TCA TCA GGC TGT GAA GCG CGC CGT GAT GAA TAT CGA ACA GAG TTT

S S G C E A R R D E Y R T E F

ACC ACA GCT TTG CAG CGC GTT GAC TTA TTC ATG GGT CAA TTC AGC

T T A L Q R V D L F M G Q F S

GAA GTC CTC TTA ACA TCT ATA TCC ACC TTC ATT AAA GGA GAC CTC

E V L L T S I S T F I K G D L

ACC ATA GCT AAT CTT GGG ACA TCA GAG GGT CGC TTC ATG CAG GTT

T I A N L G T S E G R F M Q V

GTG GTT TCT CGA TCA GGA CCA TCA ACC CCT CAT GTG AAT TTT CTC

V V S R S G P S T P H V N F L

CTG GAC TCC CAT CCA GTG TCT CCA GAA GTG ATT GTG GAG CAT ACA

L D S H P V S P E V I V E H T

TTA AAC CAA AAT GGC CAC ACA CTG GTT ATC ACT GGG AAG AAG ATC

L N Q N G H T L V I T G K K I

ACG AAG ATC CCA TTG AAT GGC TTG GGC TGC AGA CAT TTC CAG TCC

T K I P L N G L G C R H F Q S

TGC AGT CAA TGC CTC TCT GCC CCA CCC TTT GTT CAG TGT GGC TGG

C S Q C L S A P P F V Q C G W

TGC CAC GAC AAA TGT GTG CGA TCG GAG GAA TGC CTG AGC GGG ACA

C H D K C V R S E E C L S G T

TGG ACT CAA CAG ATC TGT CTG CCT GCA ATC TAC AAG GTT TTC CCA

W T Q Q I C L P A I Y K V F P

AAT AGT GCA CCC CTT GAA GGA GGG ACA AGG CTG ACC ATA TGT GGC

N S A P L E G G T R L T I C G

TGG GAC TTT GGA TTT CGG AGG AAT AAT AAA TTT GAT TTA AAG AAA

W D F G F R R N N K F D L K K

ACT AGA GTT CTC CTT GGA AAT GAG AGC TGC ACC TTG ACT TTA AGT

T R V L L G N E S C T L T L S

GAG AGC ACG ATG AAT ACA TTG AAA TGC ACA GTT GGT CCT GCC ATG

E S T M N T L K C T V G P A M

AAT AAG CAT TTC AAT ATG TCC ATA ATT ATT TCA AAT GGC CAC GGG

N K H F N M S I I I S N G H G

ACA ACA CAA TAC AGT ACA TTC TCC TAT GTG GAT CCT GTA ATA ACA

T T Q Y S T F S Y V D P V I T

AGT ATT TCG CCG AAA TAC GGT CCT ATG GCT GGT GGC ACT TTA CTT

S I S P K Y G P M A G G T L L

ACT TTA ACT GGA AAT TAC CTA AAC AGT GGG AAT TCT AGA CAC ATT

T L T G N Y L N S G N S R H I

TCA ATT GGT GGA AAA ACA TGT ACT TTA AAA AGG TGT TGT AAA TTT

S I G G K T C T L K R C C K F

ATT TTT TGT TGC ATC TGT CAA TTT GAA TTA ATA TCT GTA CCT TAA

I F C C I C Q F E L I S V P *

>EU826573,020F12,splice variant of MET,MET-697

ATG AAG GCC CCC GCT GTG CTT GCA CCT GGC ATC CTC GTG CTC CTG

M K A P A V L A P G I L V L L

TTT ACC TTG GTG CAG AGG AGC AAT GGG GAG TGT AAA GAG GCA CTA

F T L V Q R S N G E C K E A L

GCA AAG TCC GAG ATG AAT GCG AAT ATG AAG TAT CAG CTT CCC AAC

A K S E M N A N M K Y Q L P N

TTC ACC GCG GAA ACA CCC ATC CAG AAT GTC ATT CTA CAT GAG CAT

F T A E T P I Q N V I L H E H

CAC ATT TTC CTT GGT GCC ACT AAC TAC ATT TAT GTT TTA AAT GAG

H I F L G A T N Y I Y V L N E

GAA GAC CTT CAG AAG GTT GCT GAG TAC AAG ACT GGG CCT GTG CTG

E D L Q K V A E Y K T G P V L

GAA CAC CCA GAT TGT TTC CCA TGT CAG GAC TGC AGC AGC AAA GCC

E H P D C F P C Q D C S S K A

AAT TTA TCA GGA GGT GTT TGG AAA GAT AAC ATC AAC ATG GCT CTA

N L S G G V W K D N I N M A L

GTT GTC GAC ACC TAC TAT GAT GAT CAA CTC ATT AGC TGT GGC AGC

V V D T Y Y D D Q L I S C G S

GTC AAC AGA GGG ACC TGC CAG CGA CAT GTC TTT CCC CAC AAT CAT

V N R G T C Q R H V F P H N H

ACT GCT GAC ATA CAG TCG GAG GTT CAC TGC ATA TTC TCC CCA CAG

T A D I Q S E V H C I F S P Q

ATA GAA GAG CCC AGC CAG TGT CCT GAC TGT GTG GTG AGC GCC CTG

I E E P S Q C P D C V V S A L

GGA GCC AAA GTC CTT TCA TCT GTA AAG GAC CGG TTC ATC AAC TTC

G A K V L S S V K D R F I N F

TTT GTA GGC AAT ACC ATA AAT TCT TCT TAT TTC CCA GAT CAT CCA

F V G N T I N S S Y F P D H P

TTG CAT TCG ATA TCA GTG AGG AGG CTA AAG GAA ACG AAA GAT GGT

L H S I S V R R L K E T K D G

TTT ATG TTT TTG ACG GAC CAG TCC TAC ATT GAT GTT TTA CCT GAG

F M F L T D Q S Y I D V L P E

TTC AGA GAT TCT TAC CCC ATT AAG TAT GTC CAT GCC TTT GAA AGC

F R D S Y P I K Y V H A F E S

AAC AAT TTT ATT TAC TTC TTG ACG GTC CAA AGG GAA ACT CTA GAT

N N F I Y F L T V Q R E T L D

GCT CAG ACT TTT CAC ACA AGA ATA ATC AGG TTC TGT TCC ATA AAC

A Q T F H T R I I R F C S I N

TCT GGA TTG CAT TCC TAC ATG GAA ATG CCT CTG GAG TGT ATT CTC

S G L H S Y M E M P L E C I L

ACA GAA AAG AGA AAA AAG AGA TCC ACA AAG AAG GAA GTG TTT AAT

T E K R K K R S T K K E V F N

ATA CTT CAG GCT GCG TAT GTC AGC AAG CCT GGG GCC CAG CTT GCT

I L Q A A Y V S K P G A Q L A

AGA CAA ATA GGA GCC AGC CTG AAT GAT GAC ATT CTT TTC GGG GTG

R Q I G A S L N D D I L F G V

TTC GCA CAA AGC AAG CCA GAT TCT GCC GAA CCA ATG GAT CGA TCT

F A Q S K P D S A E P M D R S

GCC ATG TGT GCA TTC CCT ATC AAA TAT GTC AAC GAC TTC TTC AAC

A M C A F P I K Y V N D F F N

AAG ATC GTC AAC AAA AAC AAT GTG AGA TGT CTC CAG CAT TTT TAC

K I V N K N N V R C L Q H F Y

GGA CCC AAT CAT GAG CAC TGC TTT AAT AGG ACA CTT CTG AGA AAT

G P N H E H C F N R T L L R N

TCA TCA GGC TGT GAA GCG CGC CGT GAT GAA TAT CGA ACA GAG TTT

S S G C E A R R D E Y R T E F

ACC ACA GCT TTG CAG CGC GTT GAC TTA TTC ATG GGT CAA TTC AGC

T T A L Q R V D L F M G Q F S

GAA GTC CTC TTA ACA TCT ATA TCC ACC TTC ATT AAA GGA GAC CTC

E V L L T S I S T F I K G D L

ACC ATA GCT AAT CTT GGG ACA TCA GAG GGT CGC TTC ATG CAG GTT

T I A N L G T S E G R F M Q V

GTG GTT TCT CGA TCA GGA CCA TCA ACC CCT CAT GTG AAT TTT CTC

V V S R S G P S T P H V N F L

CTG GAC TCC CAT CCA GTG TCT CCA GAA GTG ATT GTG GAG CAT ACA

L D S H P V S P E V I V E H T

TTA AAC CAA AAT GGC TAC ACA CTG GTT ATC ACT GGG AAG AAG ATC

L N Q N G Y T L V I T G K K I

ACG AAG ACC CCA TTG AAT GGC TTG GGC TGC AGA CAT TTC CAG TCC

T K T P L N G L G C R H F Q S

TGC AGT CAA TGC CTC TCT GCC CCA CCC TTT GTT CAG TGT GGC TGG

C S Q C L S A P P F V Q C G W

TGC CAC GAC AAA TGT GTG CGA TCG GAG GAA TGC CTG AGC GGG ACA

C H D K C V R S E E C L S G T

TGG ACT CAA CAG ATC TGT CTG CCT GCA ATC TAC AAG GTT TTC CCA

W T Q Q I C L P A I Y K V F P

AAT AGT GCA CCC CTT GAA GGA GGG ACA AGG CTG ACC ATA TGT GGC

N S A P L E G G T R L T I C G

TGG GAC TTT GGA TTT CGG AGG AAT AAT AAA TTT GAT TTA AAG AAA

W D F G F R R N N K F D L K K

ACT AGA GTT CTC CTT GGA AAT GAG AGC TGC ACC TTG ACT TTA AGT

T R V L L G N E S C T L T L S

GAG AGC ACG ATG AAT ACA TTG AAA TGC ACA GTT GGT CCT GCC ATG

E S T M N T L K C T V G P A M

AAT AAG CAT TTC AAT ATG TCC ATA ATT ATT TCA AAT GGC CAC GGG

N K H F N M S I I I S N G H G

ACA ACA CAG TAC AGT ACA TTC TCC TAT GTG TTA CTT TGT TTT GTT

T T Q Y S T F S Y V L L C F V

TTT ATC TCC CCT CCA GGA TCC TGT AAT AAC AAG TAT TTC GCC GAA

F I S P P G S C N N K Y F A E

ATA CGG TCC TAT GGC TGG TGG CAC TTT ACT TAC TTT AAC TGG AAA

I R S Y G W W H F T Y F N W K

TTA CCT AAA CAG TGG GAA TTC TAG

L P K Q W E F *

>EU826574,020G03,splice variant of MET,MET-691

ATG AAG GCC CCC GCT GTG CTT GCA CCT GGC ATC CTC GTG CTC CTG

M K A P A V L A P G I L V L L

TTT ACC TTG GTG CAG AGG AGC AAT GGG GAG TGT AAA GAG GCA CTA

F T L V Q R S N G E C K E A L

GCA AAG TCC GAG ATG AAT GTG AAT ATG AAG TAT CAG CTT CCC AAC

A K S E M N V N M K Y Q L P N

TTC ACC GCG GAA ACA CCC ATC CAG AAT GTC ATT CTA CAT GAG CAT

F T A E T P I Q N V I L H E H

CAC ATT TTC CTT GGT GCC ACT AAC TAC ATT TAT GTT TTA AAT GAG

H I F L G A T N Y I Y V L N E

GAA GAC CTT CAG AAG GTT GCT GAG TAC AAG ACT GGG CCT GTG CTG

E D L Q K V A E Y K T G P V L

GAA CAC CCA GAT TGT TTC CCA TGT CAG GAC TGC AGC AGC AAA GCC

E H P D C F P C Q D C S S K A

AAT TTA TCA GGA GGT GTT TGG AAA GAT AAC ATC AAC ATG GCT CTA

N L S G G V W K D N I N M A L

GTT GTC GAC ACC TAC TAT GAT GAT CAA CTC ATT AGC TGT GGC AGC

V V D T Y Y D D Q L I S C G S

GTC AAC AGA GGG ACC TGC CAG CGA CAT GTC TTT CCC CAC AAT CAT

V N R G T C Q R H V F P H N H

ACT GCT GAC ATA CAG TCG GAG GTT CAC TGC ATA TTC TCC CCA CAG

T A D I Q S E V H C I F S P Q

ATA GAA GAG CCC AGC CAG TGT CCT GAC TGT GTG GTG AGC GCC CTG

I E E P S Q C P D C V V S A L

GGA GCC AAA GTC CTT TCA TCT GTA AAG GAC CGG TTC ATC AAC TTC

G A K V L S S V K D R F I N F

TTT GTA GGC AAT ACC ATA AAT TCT TCT TAT TTC CCA GAT CAT CCA

F V G N T I N S S Y F P D H P

TTG CAT TCG ATA TCA GTG AGA AGG CTA AAG GAA ACG AAA GAT GGT

L H S I S V R R L K E T K D G

TTT ATG TTT TTG ACG GAC CAG TCC TAC ATT GAT GTT TTA CCT GAG

F M F L T D Q S Y I D V L P E

TTC AGA GAT TCT TAC CCC ATT AAG TAT GTC CAT GCC TTT GAA AGC

F R D S Y P I K Y V H A F E S

AAC AAT TTT ATT TAC TTC TTG ACG GTC CAA AGG GAA ACT CTA GAT

N N F I Y F L T V Q R E T L D

GCT CAG ACT TTT CAC ACA AGA ATA ATC AGG TTC TGT TCC ATA AAC

A Q T F H T R I I R F C S I N

TCT GGA TTG CAT TCC TAC ATG GAA ATG CCT CTG GAG TGT ATT CTC

S G L H S Y M E M P L E C I L

ACA GAA AAG AGA AAA AAG AGA TCC ACA AAG AAG GAA GTG TTT AAT

T E K R K K R S T K K E V F N

ATA CTT CAG GCT GCG TAT GTC AGC AAG CCT GGG GCC CAG CTT GCT

I L Q A A Y V S K P G A Q L A

AGA CAA ATA GGA GCC AGC CTG AAT GAT GAC ATT CTT TTC GGG GTG

R Q I G A S L N D D I L F G V

TTC GCA CAA AGC AAG CCA GAT TCT GCC GAA CCA ATG GAT CGA TCT

F A Q S K P D S A E P M D R S

GCC ATG TGT GCA TTC CCT ATC AAA TAT GTC AAC GAC TTC TTC AAC

A M C A F P I K Y V N D F F N

AAG ATC GTC AAC AAA AAC AAT GTG AGA TGT CTC CAG CAT TTT TAC

K I V N K N N V R C L Q H F Y

GGA CCC AAT CAT GAG CAC TGC TTT AAT AGG ACA CTT CTG AGA AAT

G P N H E H C F N R T L L R N

TCA TCA GGC TGT GAA GCG CGC CGT GAT GAA TAT CGA ACA GAG TTT

S S G C E A R R D E Y R T E F

ACC ACA GCT TTG CAG CGC GTT GAC TTA TTC ATG GGT CAA TTC AGC

T T A L Q R V D L F M G Q F S

GAA GTC CTC TTA ACA TCT ATA TCC ACC TTC ATT AAA GGA GAC CTC

E V L L T S I S T F I K G D L

ACC ATA GCT AAT CTT GGG ACA TCA GAG GGT CGC TTC ATG CAG GTT

T I A N L G T S E G R F M Q V

GTG GTT TCT CGA TCA GGA CCA TCA ACC CCT CAT GTG AAT TTT CTC

V V S R S G P S T P H V N F L

CTG GAC TCC CAT CCA GTG TCT CCA GAA GTG ATT GTG GAG CAT ACA

L D S H P V S P E V I V E H T

TTA AAC CAA AAT GGC TAC ACA CTG GTT ATC ACT GGG AAG AAG TGT

L N Q N G Y T L V I T G K K C

GGC TGG CGC CAC GAC AAA TGT GTG CGA TCG GAG GAA TGC CTG AGC

G W R H D K C V R S E E C L S

GGG ACA TGG ACT CAA CAG ATC TGT CTG CCT GCA ATC TAC AAG GTT

G T W T Q Q I C L P A I Y K V

TTC CCA AAT AGT GCA CCC CTT GAA GGA GGG ACA AGG CTG ACC ATA

F P N S A P L E G G T R L T I

TGT GGC TGG GAC TTT GGA TTT CGG AGG AAT AAT AAA TTT GAT TTA

C G W D F G F R R N N K F D L

AAG AAA ACT AGA GTT CTC CTT GGA AAT GAG AGC TGC ACC TTG ACT

K K T R V L L G N E S C T L T

TTA AGT GAG AGC ACG ATG AAT ACA TTG AAA TGC ACA GTT GGT CCT

L S E S T M N T L K C T V G P

GCC ATG AAT AAG CAT TTC AAT ATG TCC ATA ATT ATT TCA AAT GGC

A M N K H F N M S I I I S N G

CAC GGG ACA ACA CAA TAC AGT ACA TTC TCC TAT GTG GAT CCT GTA

H G T T Q Y S T F S Y V D P V

ATA ACA AGT ATT TCG CCG AAA TAC GGT CCT ATG GCT GGT GGC ACT

I T S I S P K Y G P M A G G T

TTA CTT ACT TTA ACT GGA AAT TAC CTA AAC AGT GGG AAT TCT AGA

L L T L T G N Y L N S G N S R

CAC ATT TCA ATT GGT GGA AAA ACA TGT ACT CTA AAA AGG TGT GGT

H I S I G G K T C T L K R C G

AAA TTT ATT TTT TGT TGC ATC TGT CAA TTT GAA TTA ATA TCT GTA

K F I F C C I C Q F E L I S V

CCT TAA

P *

>EU826575,020F08,splice variant of MET,MET-664

ATG AAG GCC CCC GCT GTG CTT GCA CCT GGC ATC CTC GTG CTC CTG

M K A P A V L A P G I L V L L

TTT ACC TTG GTG CAG AGG AGC AAT GGG GAG TGT AAA GAG GCA CTA

F T L V Q R S N G E C K E A L

GCA AAG TCC GAG ATG AAT GTG AAT ATG AAG TAT CAG CTT CCC AAC

A K S E M N V N M K Y Q L P N

TTC ACC GCG GAA ACA CCC ATC CAG AAT GTC ATT CTA CAT GAG CAT

F T A E T P I Q N V I L H E H

CAC ATT TTC CTT GGT GCC ACT AAC TAC ATT TAT GTT TTA AAT GAG

H I F L G A T N Y I Y V L N E

GAA GAC CTT CAG AAG GTT GCT GAG TAC AAG ACT GGG CCT GTG CTG

E D L Q K V A E Y K T G P V L

GAA CAC CCA GAT TGT TTC CCA TGT CAG GAC TGC AGC AGC AAA GCC

E H P D C F P C Q D C S S K A

AAT TTA TCA GGA GGT GTT TGG AAA GAT AAC ATC AAC ATG GCT CTA

N L S G G V W K D N I N M A L

GTT GTC GAC ACC TAC TAT GAT GAT CAA CTC ATT AGC TGT GGC AGC

V V D T Y Y D D Q L I S C G S

GTC AAC AGA GGG ACC TGC CAG CGA CAT GTC TTT CCC CAC AAT CAT

V N R G T C Q R H V F P H N H

ACT GCT GAC ATA CAG TCG GAG GTT CAC TGC ATA TTC TCC CCA CAG

T A D I Q S E V H C I F S P Q

ATA GAA GAG CCC AGC CAG TGT CCT GAC TGT GTG GTG AGC GCC CTG

I E E P S Q C P D C V V S A L

GGA GCC AAA GTC CTT TCA TCT GTA AAG GAC CGG TTC ATC AAC TTC

G A K V L S S V K D R F I N F

TTT GTA GGC AAT ACC ATA AAT TCT TCT TAT TTC CCA GAT CAT CCA

F V G N T I N S S Y F P D H P

TTG CAT TCG ATA TCA GTG AGA AGG CTA AAG GAA ACG AAA GAT GGT

L H S I S V R R L K E T K D G

TTT ATG TTT TTG ACG GAC CAG TCC TAC ATT GAT GTT TTA CCT GAG

F M F L T D Q S Y I D V L P E

TTC AGA GAT TCT TAC CCC ATT AAG TAT GTC CAT GCC TTT GAA AGC

F R D S Y P I K Y V H A F E S

AAC AAT TTT ATT TAC TTC TTG ACG GTC CAA AGG GAA ACT CTA GAT

N N F I Y F L T V Q R E T L D

GCT CAG ACT TTT CAC ACA AGA ATA ATC AGG TTC TGT TCC ATA AAC

A Q T F H T R I I R F C S I N

TCT GGA TTG CAT TCC TAC ATG GAA ATG CCT CTG GAG TGT ATT CTC

S G L H S Y M E M P L E C I L

ACA GAA AAG AGA AAA AAG AGA TCC ACA AAG AAG GAA GTG TTT AAT

T E K R K K R S T K K E V F N

ATA CTT CAG GCT GCG TAT GTC AGC AAG CCT GGG GCC CAG CTT GCT

I L Q A A Y V S K P G A Q L A

AGA CAA ATA GGA GCC AGC CTG AAT GAT GAC ATT CTT TTC GGG GTG

R Q I G A S L N D D I L F G V

TTC GCA CAA AGC AAG CCA GAT TCT GCC GAA CCA ATG GAT CGA TCT

F A Q S K P D S A E P M D R S

GCC ATG TGT GCA TTC CCT ATC AAA TAT GTC AAC GAC TTC TTC AAC

A M C A F P I K Y V N D F F N

AAG ATC GTC AAC AAA AAC AAT GTG AGA TGT CTC CAG CAT TTT TAC

K I V N K N N V R C L Q H F Y

GGA CCC AAT CAT GAG CAC TGC TTT AAT AGG ACA CTT CTG AGA AAT

G P N H E H C F N R T L L R N

TCA TCA GGC TGT GAA GCG CGC CGT GAT GAA TAT CGA ACA GAG TTT

S S G C E A R R D E Y R T E F

ACC ACA GCT TTG CAG CGC GTT GAC TTA TTC ATG GGT CAA TTC AGC

T T A L Q R V D L F M G Q F S

GAA GTC CTC TTA ACA TCT ATA TCC ACC TTC ATT AAA GGA GAC CTC

E V L L T S I S T F I K G D L

ACC ATA GCT AAT CTT GGG ACA TCA GAG GGT CGC TTC ATG CAG GTT

T I A N L G T S E G R F M Q V

GTG GTT TCT CGA TCA GGA CCA TCA ACC CCT CAT GTG AAT TTT CTC

V V S R S G P S T P H V N F L

ATG GAC TCC CAT CCA GTG TCT CCA GAA GTG ATT GTG GAG CAT ACA

M D S H P V S P E V I V E H T

TTA AAC CAA AAT GGC TAC ACA CTG GTT ATC ACT GGG AAG AAG ATC

L N Q N G Y T L V I T G K K I

ACG AAG ATC CCA TTG AAT GGC TTG GGC TGC AGA CAT TTC CAG TCC

T K I P L N G L G C R H F Q S

TGC AGT CAA TGC CTC TCT GCC CCA CCC TTT GTT CAG TGT GGC TGG

C S Q C L S A P P F V Q C G W

TGC CAC GAC AAA TGT GTG CGA TCG GAG GAA TGC CTG AGC GGG ACA

C H D K C V R S E E C L S G T

TGG ACT CAA CAG ATC TGT CTG CCT GCA ATC TAC AAG GTT TTC CCA

W T Q Q I C L P A I Y K V F P

AAT AGT GCA CCC CTT GAA GGA GGG ACA AGG CTG ACC ATA TGT GGC

N S A P L E G G T R L T I C G

TGG GAC TTT GGA TTT CGG AGG AAT AAT AAA TTT GAT TTA AAG AAA

W D F G F R R N N K F D L K K

ACT AGA GTT CTC CTT GGA AAT GAG AGC TGC ACC TTG ACT TTA AGT

T R V L L G N E S C T L T L S

GAG AGC ACG ATG AAT ACA TTG AAA TGC ACA GTT GGT CCT GCC ATG

E S T M N T L K C T V G P A M

AAT AAG CAT TTC AAT ATG TCC ATA ATT ATT TCA AAT GGC CAC GGG

N K H F N M S I I I S N G H G

ACA ACA CAA TAC AGT ACA TTC TCC TAT GTG GTA AGG AAG ATT CTA

T T Q Y S T F S Y V V R K I L

TCC TAT CAT GTT tga

S Y H V *

>EU826576,020E11,splice variant of MET,MET-621

ATG AAG GCC CCC GCT GTG CTT GCA CCT GGC ATC CTC GTG CTC CTG

M K A P A V L A P G I L V L L

TTT ACC TTG GTG CAG AGG AGC AAT GGG GAG TGT AAA GAG GCA CTA

F T L V Q R S N G E C K E A L

GCA AAG TCC GAG ATG AAT GTG AAT ATG AAG TAT CAG CTT CCC AAC

A K S E M N V N M K Y Q L P N

TTC ACC GCG GAA ACA CCC ATC CAG AAT GTC ATT CTA CAT GAG CAT

F T A E T P I Q N V I L H E H

CAC ATT TTC CTT GGT GCC ACT AAC TAC ATT TAT GTT TTA AAT GAG

H I F L G A T N Y I Y V L N E

GAA GAC CTT CAG AAG GTT GCT GAG TAC AAG ACT GGG CCT GTG CTG

E D L Q K V A E Y K T G P V L

GAA CAC CCA GAT TGT TTC CCA TGT CAG GAC TGC AGC AGC AAA GCC

E H P D C F P C Q D C S S K A

AAT TTA TCA GGA GGT GTT TGG AAA GAT AAC ATC AAC ATG GCT CTA

N L S G G V W K D N I N M A L

GTT GTC GAC ACC TAC TAT GAT GAT CAA CTC ATT AGC TGT GGC AGC

V V D T Y Y D D Q L I S C G S

GTC AAC AGA GGG ACC TGC CAG CGA CAT GTC TTT CCC CAC AAT CAT

V N R G T C Q R H V F P H N H

ACT GCT GAC ATA CAG TCG GAG GCT CAC TGC ATA TTC TCC CCA CAG

T A D I Q S E A H C I F S P Q

ATA GAA GAG CCC AGT CAG TGT CCT GAC TGT GTG GTG AGC GCC CTG

I E E P S Q C P D C V V S A L

GGA GCC AAA GTC CTT TCA TCT GTA AAG GAC CGG TTC ATC AAC TTC

G A K V L S S V K D R F I N F

TTT GTA GGC AAT ACC ATA AAT TCT TCT TAT TTC CCA GAT CAT CCA

F V G N T I N S S Y F P D H P

TTG CAT TCG ATA TCA GTG AGA AGG CTA AAG GAA ACG AAA GAT GGT

L H S I S V R R L K E T K D G

TTT ATG TTT TTG ACG GAC CAG TCC TAC ATT GAT GTT TTA CCT GAG

F M F L T D Q S Y I D V L P E

TTC AGA GAT TCT TAC CCC ATT AAG TAT GTC CAT GCC TTT GAA AGC

F R D S Y P I K Y V H A F E S

AAC AAT TTT ATT TAC TTC TTG ACG GTC CAA AGG GAA ACT CTA GAT

N N F I Y F L T V Q R E T L D

GCT CAG ACT TTT CAC ACA AGA ATA ATC AGG TTC TGT TCC ATA AAC

A Q T F H T R I I R F C S I N

TCT GGA TTG CAT TCC TAC ATG GAA ATG CCT CTG GAG TGT ATT CTC

S G L H S Y M E M P L E C I L

ACA GAA AAG AGA AAA AAG AGA TCC ACA AAG AAG GAA GTG TTT AAT

T E K R K K R S T K K E V F N

ATA CTT CAG GCT GCG TAT GTC AGC AAG CCT GGG GCC CAG CTT GCT

I L Q A A Y V S K P G A Q L A

AGA CAA ATA GGA GCC AGC CTG AAT GAT GAC ATT CTT TTC GGG GTG

R Q I G A S L N D D I L F G V

TTC GCA CAA AGC AAG CCA GAT TCT GCC GAA CCA ATG GAT CGA TCT

F A Q S K P D S A E P M D R S

GCC ATG TGT GCA TTC CCT ATC AAA TAT GTC AAC GAC TTC TTC AAC

A M C A F P I K Y V N D F F N

AAG ATC GTC AAC AAA AAC AAT GTG AGA TGT CTC CAG CAT TTT TAC

K I V N K N N V R C L Q H F Y

GGA CCC AAT CAT GAG CAC TGC TTT AAT AGG ACA CTT CTG AGA AAT

G P N H E H C F N R T L L R N

TCA TCA GGC TGT GAA GCG CGC CGT GAT GAA TAT CGA ACA GAG TTT

S S G C E A R R D E Y R T E F

ACC ACA GCT TTG CAG CGC GTT GAC TTA TTC ATG GGT CAA TTC AGC

T T A L Q R V D L F M G Q F S

GAA GTC CTC TTA ACA TCT ATA TCC ACC TTC ATT AAA GGA GAC CTC

E V L L T S I S T F I K G D L

ACC ATA GCT AAT CTT GGG ACA TCA GAG GGT CGC TTC ATG CAG GTT

T I A N L G T S E G R F M Q V

GTG GTT TCT CGA TCA GGA CCA TCA ACC CCT CAT GTG AAT TTT CTC

V V S R S G P S T P H V N F L

CTG GAC TCC CAT CCA GTG TCT CCA GAA GTG ATT GTG GAG CAT ACA

L D S H P V S P E V I V E H T

TTA AAC CAA AAT GGC TAC ACA CTG GTT ATC ACT GGG AAG AAG ATC

L N Q N G Y T L V I T G K K I

ACG AAG ATC CCA TTG AAT GGC TTG GGC TGC AGA CAT TTC CAG TCC

T K I P L N G L G C R H F Q S

TGC AGT CAA TGC CTC TCT GCC CCA CCC TTT GTT CAG TGT GGC TGG

C S Q C L S A P P F V Q C G W

TGC CAC GAC AAA TGT GTG CGA TCG GAG GAA TGC CTG AGC GGG ACA

C H D K C V R S E E C L S G T

TGG ACT CAA CAG ATC TGT CTG CCT GCA ATC TAC AAG GTT TTC CCA

W T Q Q I C L P A I Y K V F P

AAT AGT GCA CCC CTT GAA GGA GGG ACA AGG CTG ACC ATA TGT GGC

N S A P L E G G T R L T I C G

TGG GAC TTT GGA TTT CGG AGG AAT AAT AAA TTT GAT TTA AAG AAA

W D F G F R R N N K F D L K K

ACT AGA GTT CTC CTT GGA AAT GAG AGC TGC ACC TTG ACT TTA AGT

T R V L L G N E S C T L T L S

GAG AGC ACG ATG AAT ACG TAA

E S T M N T *

>EU826577,020D07,splice variant of MET,MET-596

ATG AAG GCC CCC GCT GTG CTT GCA CCT GGC ATC CTC GTG CTC CTG

M K A P A V L A P G I L V L L

TTT ACC TTG GTG CAG AGG AGC AAT GGG GAG TGT AAA GAG GCA CTA

F T L V Q R S N G E C K E A L

GCA AAG TCC GAG ATG AAT GTG AAT ATG AAG TAT CAG CTT CCC AAC

A K S E M N V N M K Y Q L P N

TTC ACC GCG GAA ACA CCC ATC CAG AAT GTC ATT CTA CAT GAG CAT

F T A E T P I Q N V I L H E H

CAC ATT TTC CTT GGT GCC ACT AAC TAC ATT TAT GTT TTA AAT GAG

H I F L G A T N Y I Y V L N E

GAA GAC CTT CAG AAG GTT GCT GAG TAC AAG ACT GGG CCT GTG CTG

E D L Q K V A E Y K T G P V L

GAA CAC CCA GAT TGT TTC CCA TGT CAG GAC TGC AGC AGC AAA GCC

E H P D C F P C Q D C S S K A

AAT TTA TCA GGA GGT GTT TGG AAA GAT AAC ATC AAC ATG GCT CTA

N L S G G V W K D N I N M A L

GTT GTC GAC ACC TAC TAT GAT GAT CAA CTC ATT AGC TGT GGC AGC

V V D T Y Y D D Q L I S C G S

GTC AAC AGA GGG ACC TGC CAG CGA CAT GTC TTT CCC CAC AAT CAT

V N R G T C Q R H V F P H N H

ACT GCT GAC ATA CAG TCG GAG GTT CAC TGC ATA TTC TCC CCA CAG

T A D I Q S E V H C I F S P Q

ATA GAA GAG CCC AGC CAG TGT CCT GAC TGT GTG GTG AGC GCC CTG

I E E P S Q C P D C V V S A L

GGA GCC AAA GTC CTT TCA TCT GTA AAG GAC CGG TTC ATC AAC TTC

G A K V L S S V K D R F I N F

TTT GTA GGC AAT ACC ATA AAT TCT TCT TAT TTC CCA GAT CAT CCA

F V G N T I N S S Y F P D H P

TTG CAT TCG ATA TCA GTG AGA AGG CTA AAG GAA ACG AAA GAT GGT

L H S I S V R R L K E T K D G

TTT ATG TTT TTG ACG GAC CAG TCC TAC ATT GAT GTT TTA CCT GAG

F M F L T D Q S Y I D V L P E

TTC AGA GAT TCT TAC CCC ATT AAG TAT GTC CAT GCC TTT GAA AGC

F R D S Y P I K Y V H A F E S

AAC AAT TTT ATT TAC TTC TTG ACG GTC CAA AGG GAA ACT CTA GAT

N N F I Y F L T V Q R E T L D

GCT CAG ACT TTT CAC ACA AGA ATA ATC AGG TTC TGT TCC ATA AAC

A Q T F H T R I I R F C S I N

TCT GGA TTG CAT TCC TAC ATG GAA ATG CCT CTG GAG TGT ATT CTC

S G L H S Y M E M P L E C I L

ACA GAA AAG AGA AAA AAG AGA TCC ACA AAG AAG GAA GTG TTT AAT

T E K R K K R S T K K E V F N

ATA CTT CAG GCT GCG TAT GTC AGC AAG CCT GGG GCC CAG CTT GCT

I L Q A A Y V S K P G A Q L A

AGA CAA ATA GGA GCC AGC CCG AAT GAT GAC ATT CTT TTC GGG GTG

R Q I G A S P N D D I L F G V

TTC GCA CAA AGC AAG CCA GAT TCT GCC GAA CCA ATG GAT CGA TCT

F A Q S K P D S A E P M D R S

GCC ATG TGT GCA TTC CCT ATC AAA TAT GTC AAC GAC TTC TTC AAC

A M C A F P I K Y V N D F F N

AAG ATC GTC AAC AAA AAC AAT GTG AGA TGT CTC CAG CAT TTT TAC

K I V N K N N V R C L Q H F Y

GGA CCC AAT CAT GAG CAC TGC TTT AAT AGG ACA CTT CTG AGA AAT

G P N H E H C F N R T L L R N

TCA TCA GGC TGT GAA GCG CGC CGT GAT GAA TAT CGA ACA GAG TTT

S S G C E A R R D E Y R T E F

ACC ACA GCT TTG CAG CGC GTT GAC TTA TTC ATG GGT CAA TTC AGC

T T A L Q R V D L F M G Q F S

GAA GTC CTC TTA ACA TCT ATA TCC ACC TTC ATT AAA GGA GAC CTC

E V L L T S I S T F I K G D L

ACC ATA GCT AAT CTT GGG ACA TCA GAG GGT CGC TTC ATG CAG GTT

T I A N L G T S E G R F M Q V

GTG GTT TCT CGA TCA GGA CCA TCA ACC CCT CAT GTG AAT TTT CTC

V V S R S G P S T P H V N F L

CTG GAC TCC CAT CCA GTG TCT CCA GAA GTG ATT GTG GAG CAT ACA

L D S H P V S P E V I V E H T

TTA AAC CAA AAT GGC TAC ACA CTG GTT ATC ACT GGG AAG AAG ATC

L N Q N G Y T L V I T G K K I

ACG AAG ATC CCA TTG AAT GGC TTG GGC TGC AGA CAT TTC CAG TCC

T K I P L N G L G C R H F Q S

TGC AGT CAA TGC CTC TCT GCC CCA CCC TTT GTT CAG TGT GGC TGG

C S Q C L S A P P F V Q C G W

TGC CAC GAC AAA TGT GTG CGA TCG GAG GAA TGC CTG AGC GGG ACA

C H D K C V R S E E C L S G T

TGG ACT CAA CAG ATC TGT CTG CCT GCA ATC TAC AAG GTA GGA ATC

W T Q Q I C L P A I Y K V G I

TCT AAC AGC TGG CAT ACA TGT TTT TGT TTG GTG TTT TTT TTT TTT

S N S W H T C F C L V F F F F

TTT TGG TTT GGT TTG GTT TGT TTT TTG TTT TTT TAG

F W F G L V C F L F F *

>EU826578,020D11,splice variant of MET,MET-408

ATG AAG GCC CCC GCT GTG CTT GCA CCT GGC ATC CTC GTG CTC CTG

M K A P A V L A P G I L V L L

TTT ACC TTG GTG CAG AGG AGC AAT GGG GAG TGT AAA GAG GCA CTA

F T L V Q R S N G E C K E A L

GCA AAG TCC GAG ATG AAT GTG AAT ATG AAG TAT CGG CTT CCC AAC

A K S E M N V N M K Y R L P N

TTC ACC GCG GAA ACA CCC ATC CAG AAT GTC ATT CTA CAT GAG CAT

F T A E T P I Q N V I L H E H

CAC ATT TTC CTT GGT GCC ACT AAC TAC ATT TAT GTT TTA AAT GAG

H I F L G A T N Y I Y V L N E

GAA GAC CTT CAG AAG GTT GCT GAG TAC AAG ACT GGG CCT GTG CTG

E D L Q K V A E Y K T G P V L

GAA CAC CCA GAT TGT TTC CCA TGT CAG GAC TGC AGC AGC AAA GCC

E H P D C F P C Q D C S S K A

AAT TTA TCA GGA GGT GTT TGG AGA GAT AAC ATC AAC ATG GCT CTA

N L S G G V W R D N I N M A L

GTT GTC GAC ACC TAC TAT GAT GAT CAA CTC ATT AGC TGT GGC AGC

V V D T Y Y D D Q L I S C G S

GTC AAC AGA GGG ACC TGC CAG CGA CAT GCC TTT CCC CAC AAT CAT

V N R G T C Q R H A F P H N H

ACT GCT GAC ATA CAG TCG GAG GTT CAC TGC ATA TTC TCC CCA CAG

T A D I Q S E V H C I F S P Q

ATA GAA GAT CCC AGC CAG TGT CCT GAC TGT GTG GTG AGC GCC CTG

I E D P S Q C P D C V V S A L

GGA GCC AAA GTC CTT TCA TCT GTA AAG GAC CGG TTC ATC AAC TTC

G A K V L S S V K D R F I N F

TTT GTA GGC AAT ACC ATA AAT TCT TCT TAT TTC CCA GAT CAT CCA

F V G N T I N S S Y F P D H P

TTG CAT TCG ATA TCA TTG AGA AGG CTA AAG GAA ACG AAA GAT GGT

L H S I S L R R L K E T K D G

TTT ATG TTT TTG ACG GAC CAG TCC TAC ATT GAT GTT TTA CCT GAG

F M F L T D Q S Y I D V L P E

TTC AGA GAT TCT TAC CCC ATT AAG TAT GTC CAT GCC TTT GAA AGC

F R D S Y P I K Y V H A F E S

AAC AAT TTT ATT TAC TTC TTG ACG GTC CAA AGG GAA ACT CTA GAT

N N F I Y F L T V Q R E T L D

GCT CAG ACT TTT CAC GCA AGA ATA ATC AGG TTC TGT TCC ATA AAC

A Q T F H A R I I R F C S I N

TCT GGA TTG CAT TCC TAC ATG GAA ATG CCT CTG GAG TGT ATT CTC

S G L H S Y M E M P L E C I L

ACA GAG AAG AGA AAA AAG AGA TCC ACA AAG AAG GAA GTG TTT AAT

T E K R K K R S T K K E V F N

ATA CTT CAG GCT GCG TAT GTC AGC AAG CCT GGG GCC CAG CTT GCT

I L Q A A Y V S K P G A Q L A

AGA CAA ATA GGA GCC AGC CTG AAT GAT GAC ATT CTT TTC GGG GTG

R Q I G A S L N D D I L F G V

TTC GCA CAA AGC AAG CCA GAT TCT GCC GAA CCA ATG GAT CGA TCT

F A Q S K P D S A E P M D R S

GCC ATG TGT GCA TTC CCT ATC AAA TAT GTC AAC GAC TTC TTC AAC

A M C A F P I K Y V N D F F N

AAG ATC GTC AAC AAA AAC AAT GTG AGA TGT CTC CAG CAT TTT TAC

K I V N K N N V R C L Q H F Y

GGA CCC AAT CAT GAG CAC TGC TTT AAT AGG GCA GAA AAT GTG CTA

G P N H E H C F N R A E N V L

GAT TGG AGG TGA

D W R *

>EU826579,020D04,splice variant of MET,MET-518

ATG AAG GCC CCC GCT GTG CTT GCA CCT GGC ATC CTC GTG CTC CTG

M K A P A V L A P G I L V L L

TTT ACC TTG GTG CAG AGG AGC AAT GGG GAG TGT AAA GAG GCA CTA

F T L V Q R S N G E C K E A L

GCA AAG TCC GAG ATG AAT GTG AAT ATG AAG TAT CAG CTT CCC AAC

A K S E M N V N M K Y Q L P N

TTC ACC GCG GAA ACA CCC ATC CAG AAT GTC ATT CTA CAT GAG CAT

F T A E T P I Q N V I L H E H

CAC ATT TTC CTT GGT GCC ACT AAC TAC ATT TAT GTT TTA AAT GAG

H I F L G A T N Y I Y V L N E

GAA GAC CTT CAG AAG GTT GCT GAG TAC AAG ACT GGG CCT GTG CTG

E D L Q K V A E Y K T G P V L

GAA CAC CCA GAT TGT TTC CCA TGT CAG GAC TGC AGC AGC AAA GCC

E H P D C F P C Q D C S S K A

AAT TTA TCA GGA GGT GTT TGG AAA GAT AAC ATT AAC ATG GCT CTA

N L S G G V W K D N I N M A L

GTT GTC GAC ACC TAC TAT GAT GAT CAA CTC ATT AGC TGT GGC AGC

V V D T Y Y D D Q L I S C G S

GTC AAC AGA GGG ACC TGC CAG CGA CAT GTC TTT CCC CAC AAT CAT

V N R G T C Q R H V F P H N H

ACT GCT GAC ATA CAG TCG GAG GTT CAC TGC ATA TTC TCC CCA CAG

T A D I Q S E V H C I F S P Q

ATA GAA GAG CCC AGC CAG TGT CCT GAC TGT GTG GTG AGC GCC CTG

I E E P S Q C P D C V V S A L

GGA GCC AAA GTC CTT TCA TCT GTA AAG GAC CGG TTC ATC AAC TTC

G A K V L S S V K D R F I N F

TTT GTA GGC AAT ACC ATA AAT TCT TCT TAT TTC CCA GAT CAT CCA

F V G N T I N S S Y F P D H P

TTG CAT TCG ATA TCA GTG AGA AGG CTA AAG GAA ACG AAA GAT GGT

L H S I S V R R L K E T K D G

TTT ATG TTT TTG ACG GAC CAG TCC TAC ATT GAT GTT TTA CCT GAG

F M F L T D Q S Y I D V L P E

TTC AGA GAT TCT TAC CCC ATT AAG TAT GTC CAT GCC TTT GAA AGC

F R D S Y P I K Y V H A F E S

AAC AAT TTT ATT TAC TTC TTG ACG GTC CAA AGG GAA ACT CTA GAT

N N F I Y F L T V Q R E T L D

GCT CAG ACT TTT CAC ACA AGA ATA ATC AGG TTC TGT TCC ATA AAC

A Q T F H T R I I R F C S I N

TCT GGA TTG CAT TCC TAC ATG GAA ATG CCT CTG GAG TGT ATT CTC

S G L H S Y M E M P L E C I L

ACA GAA AAG AGA AAA AAG AGA TCC ACA AAG AAG GAA GTG TTT AAT

T E K R K K R S T K K E V F N

ATA CTT CAG GCT GCG TAT GTC AGC AAG CCT GGG GCC CAG CTT GCT

I L Q A A Y V S K P G A Q L A

AGA CAA ATA GGA GCC AGC CTG AAT GAT GTC ATT CTT TTC GGG GTG

R Q I G A S L N D V I L F G V

TTC GCA CAA AGC AAG CCA GAT TCT GCC GAA CCA ATG GAT CGA TCT

F A Q S K P D S A E P M D R S

GCC ATG TGT GCA TTC CCT ATC AAA TAT GTC AAC GAC TTC TTC AAC

A M C A F P I K Y V N D F F N

AAG ATC GTC AAC AAA AAC AAT GTG AGA TGT CTC CAG CAT TTT TAC

K I V N K N N V R C L Q H F Y

GGA CCC AAT CAT GAG CAC TGC TTT AAT AGG ACA CTT CTG AGA AAT

G P N H E H C F N R T L L R N

TCA TCA GGC TGT GAA GCG CGC CGT GAT GAA TAT CGA ACA GAG TTT

S S G C E A R R D E Y R T E F

ACC ACA GCT TTG CAG CGC GTT GAC TTA TTC ATG GGT CAA TTC AGC

T T A L Q R V D L F M G Q F S

GAA GTC CTC TTA ACA TCT ATA TCC ACC TTC ATT AAA GGA GAC CTC

E V L L T S I S T F I K G D L

ACC ATA GCT AAT CTT GGG ACA TCA GAG GGT CGC TTC ATG CAG GTT

T I A N L G T S E G R F M Q V

GTG GTT TCT CGA TCA GGA CCA TCA ACC CCT CAT GTG AAT TTT CTC

V V S R S G P S T P H V N F L

CTG GAC TCC CAT CCA GTG TCT CCA GAA GTG ATT GTG GAG CAT ACA

L D S H P V S P E V I V E H T

TTA AAC CAA AAT GAC TAC ACA CTG GTT ATC ACT GGG AAG GAG GTA

L N Q N D Y T L V I T G K E V

AGC TGT TCC CAC AGG GAA TTT CCA tga

S C S H R E F P *

>EU826580,020C12,splice variant of MET,MET-468

ATG AAG GCC CCC GCT GTG CTT GCA CCT GGC ATC CTC GTG CTC CTG

M K A P A V L A P G I L V L L

TTT ACC TTG GTG CAG AGG AGC AAT GGG GAG TGT AAA GAG GCA CTA

F T L V Q R S N G E C K E A L

GCA AAG TCC GAG ATG AAT GTG AAT ATG AAG TAT CAG CTT CCC AAC

A K S E M N V N M K Y Q L P N

TTC ACC GCG GAA ACA CCC ATC CAG AAT GTC ATT CTA CAT GAG CAT

F T A E T P I Q N V I L H E H

CAC ATT TTC CTT GGT GCC ACT AAC TAC ATT TAT GTT TTA AAT GAG

H I F L G A T N Y I Y V L N E

GAA GAC CTT CAG AAG GTT GCT GAG TAC AAG ACT GGG CCT GTG CTG

E D L Q K V A E Y K T G P V L

GAA CAC CCA GAT TGT TTC CCA TGT CAG GAC TGC AGC AGC AAA GCC

E H P D C F P C Q D C S S K A

AAT TTA TCA GGA GGT GTT TGG AAA AAT AAC ATC AAC ATG GCT CTA

N L S G G V W K N N I N M A L

GTT GTC GAC ACC TAC TAT GAT GAT CAA CTC ATT AGC TGT GGC AGC

V V D T Y Y D D Q L I S C G S

GTC AAC AGA GGG ACC TGC CAG CGA CAT GTC TTT CCC CAC AAT CAT

V N R G T C Q R H V F P H N H

ACT GCT GAC ATA CAG TCG GAG GTT CAC TGC ATA TTC TCC CCA CAG

T A D I Q S E V H C I F S P Q

ATA GAA GAG CCC AGC CAG TGT CCT GAC TGT GTG GTG AGC GCC CTG

I E E P S Q C P D C V V S A L

GGA GCC AAA GTC CTT TCA TCT GTA AAG GAC CGG TTC ATC AAC TTC

G A K V L S S V K D R F I N F

TTT GTA GGC AAT ACC ATA AAT TCT TCT TAT TTC CCA GAT CAT CCA

F V G N T I N S S Y F P D H P

TTG CAT TCG ATA TCA GTG AGA AGG CTA AAG GAA ACG AAA GAT GGT

L H S I S V R R L K E T K D G

TTT ATG TTT TTG ACG GAC CAG TCC TAC ATT GAT GTT TTA CCT GAG

F M F L T D Q S Y I D V L P E

TTC AGA GAT TCT TAC CCC ATT AAG TAT GTC CAT GCC TTT GAA AGC

F R D S Y P I K Y V H A F E S

AAC AAT TTT ATT TAC TTC TTG ACG GTC CAA AGG GAA ACT CTA GAT

N N F I Y F L T V Q R E T L D

GCT CAG ACT TTT CAC ACA AGA ATA ATC AGG TTC TGT TCC ATA AAC

A Q T F H T R I I R F C S I N

TCT GGA TTG CAT TCC TAC ATG GAA ATG CCT CTG GAG TGT ATT CTC

S G L H S Y M E M P L E C I L

ACA GAA AAG AGA AAA AAG AGA TCC ACA AAG AAG GAA GTG TTT AAT

T E K R K K R S T K K E V F N

ATA CTT CAG GCT GCG TAT GTC AGC AAG CCT GGG GCC CAG CTT GCT

I L Q A A Y V S K P G A Q L A

AGA CAA ATA GGA GCC AGC CTG AAT GAT GAC ATT CTT TTC GGG GTG

R Q I G A S L N D D I L F G V

TTC GCA CAA AGC AAG CCA GAT TCT GCC GAA CCA ATG GAT CGA TCT

F A Q S K P D S A E P M D R S

GCC ATG TGT GCA TTC CCT ATC AAA TAT GTC AAC GAC TTC TTC AAC

A M C A F P I K Y V N D F F N

AAG ATC GTC AAC AAA AAC AAT GTG AGA TGT CTC CAG CAT TTT TAC

K I V N K N N V R C L Q H F Y

GGA CCC AAT CAT GAG CAC TGC TTT AAT AGG ACA CTT CTG AGA AAT

G P N H E H C F N R T L L R N

TCA TCA GGC TGT GAA GCG CGC CGT GAT GAA TAT CGA ACA GAG TTT

S S G C E A R R D E Y R T E F

ACC ACA GCT TTG CAG CGC GTT GAC TTA TTC ATG GGT CAA TTC AGC

T T A L Q R V D L F M G Q F S

GAA GTC CTC TTA ACA TCT ATA TCC ACC TTC ATT AAA GGA GAC CTC

E V L L T S I S T F I K G D L

ACC ATA GCT AAT CTT GGG ACA TCA GAG GGT CGC TTC ATG CAG GTA

T I A N L G T S E G R F M Q V

AGT GCT TTC TGA

S A F *

>EU826581,020C10,splice variant of MET,MET-413

ATG AAG GCC CCC GCT GTG CTT GCA CCT GGC ATC CTC GTG CTC CTG

M K A P A V L A P G I L V L L

TTT ACC TTG GTG CAG AGG AGC AAT GGG GAG TGT AAA GAG GCA CTA

F T L V Q R S N G E C K E A L

GCA AAG TCC GAG ATG AAT GTG AAT ATG AAG TAT CAG CTT CCC AAC

A K S E M N V N M K Y Q L P N

TTC ACC GCG GAA ACA CCC ATC CAG AAT GTC ATT CTA CAT GAG CAT

F T A E T P I Q N V I L H E H

CAC ATT TTC CTT GGT GCC ACT AAC TAC ATT TAT GTT TTA AAT GAG

H I F L G A T N Y I Y V L N E

GAA GAC CTT CAG AAG GTT GCT GAG TAC AAG ACT GGG CCT GTG CTG

E D L Q K V A E Y K T G P V L

GAA CAC CCA GAT TGT TTC CCA TGT CAG GAC TGC AGC AGC AAA GCC

E H P D C F P C Q D C S S K A

AAT TTA TCA GGA GGT GTT TGG AAA GAT AAC ATC AAC ATG GCT CTA

N L S G G V W K D N I N M A L

GTT GTC GAC ACC TAC TAT GAT GAT CAA CTC ATT AGC TGT GGC AGC

V V D T Y Y D D Q L I S C G S

GTC AAC AGA GGG ACC TGC CAG CGA CAT GTC TTT CCC CAC AAT CAT

V N R G T C Q R H V F P H N H

ACT GCT GAT ATA CAG TCG GAG GTT CAC TGC ATA TTC TCC CCA CAG

T A D I Q S E V H C I F S P Q

ATA GAA GAG CCC AGC CAG TGT CCT GAC TGT GTG GTG AGC GCC CTG

I E E P S Q C P D C V V S A L

GGA GCC AAA GTC CTT TCA TCT GTA AAG GAC CGG TTC ATC AAC TTC

G A K V L S S V K D R F I N F

TTT GTA GGC AAT ACC ATA AAT TCT TCT TAT TTC CCA GAT CAT CCA

F V G N T I N S S Y F P D H P

TTG CAT TCG ATA TCA GTG AGA AGG CTA AAG GAA ACG AAA GAT GGT

L H S I S V R R L K E T K D G

TTT ATG TTT TTG ACG GAC CAG TCC TAC ATT GAT GTT TTA CCT GAG

F M F L T D Q S Y I D V L P E

TTC AGA GAT TCT TAC CCC ATT AAG TAT GTC CAT GCC TTT GAA AGC

F R D S Y P I K Y V H A F E S

AAC AAT TTT ATT TAC TTC TTG ACG GTC CAA AGG GAA ACT CTA GAT

N N F I Y F L T V Q R E T L D

GCT CAG ACT TTT CAC ACA AGA ATA ATC AGG TTC TGT TCC ATA AAC

A Q T F H T R I I R F C S I N

TCT GGA TTG CAT TCC TAC ATG GAA ATG CCT CTG GAG TGT ATT CTC

S G L H S Y M E M P L E C I L

ACA GAA AAG AGA AAA AAG AGA TCC ACA AAG AAG GAA GTG TTA AAT

T E K R K K R S T K K E V L N

ATA CTT CAG GCT GCG TAT GTC AGC AAG CCT GGG GCC CAG CTT GCT

I L Q A A Y V S K P G A Q L A

AGG CAA ATA GGA GCC AGC CTG AAT GAT GAC ATT CTT TTC GGG GTG

R Q I G A S L N D D I L F G V

TTC GCA CAA AGC AAG CCA GAT TCT GCC GAA CCA ATG GAT CGA TCT

F A Q S K P D S A E P M D R S

GCC ATG TGT GCA TTC CCT ATC AAA TAT GTC AAC GAC TTC TTC AAC

A M C A F P I K Y V N D F F N

AAG ATC GTC AAC AAA AAC AAT GTG AGA TGT CTC CAG CAT TTT TAC

K I V N K N N V R C L Q H F Y

GGA CCC AAT CAT GAG CAC TGC TTT AAT AGG GTA AGT CAC ATC AGT

G P N H E H C F N R V S H I S

TCC CCA CTT ATA AAC TGT GAG GTA TAA

S P L I N C E V *

>EU826582,004C11,splice variant of RON,RON-495

ATG GAG CTC CTC CCG CCG CTG CCT CAG TCC TTC CTG TTG CTG CTG

M E L L P P L P Q S F L L L L

CTG TTG CCT GCC AAG CCC GCG GCG GGC GAG GAC TGG CAG TGC CCG

L L P A K P A A G E D W Q C P

CGC ACC CCC TAC GCG GCC TCT CGC GAC TTT GAC GTG AAG TAC GTG

R T P Y A A S R D F D V K Y V

GTG CCC AGC TTC TCC GCC GGA GGC CTG GTA CAG GCC ATG GTG ACC

V P S F S A G G L V Q A M V T

TAC GAG GGC GAC AGA AAT GAG AGT GCT GTG TTT GTA GCC ATA CGC

Y E G D R N E S A V F V A I R

AAT CGC CTG CAT GTG CTT GGG CCT GAC CTG AAG TCT GTC CAG AGC

N R L H V L G P D L K S V Q S

CTG GCC ACG GGC CCT GCT GGA GAC CCT GGC TGC CAG ACG TGT GCA

L A T G P A G D P G C Q T C A

GCC TGT GGC CCA GGA CCC CAC GGC CCT CCC GGT GAC ACA GAC ACA

A C G P G P H G P P G D T D T

AAG GTG CTG GTG CTG GAT CCC GCG CTG CCT GCG CTG GTC AGT TGT

K V L V L D P A L P A L V S C

GGC TCC AGC CTG CAG GGC CGC TGC TTC CTG CAT GAC CTA GAG CCC

G S S L Q G R C F L H D L E P

CAA GGG ACA GCC GTG CAT CTG GCA GCG CCA GCC TGC CTC TTC TCA

Q G T A V H L A A P A C L F S

GCC CAC CAT AAC CGG CCC GAT GAC TGC CCC GAC TGT GTG GCC AGC

A H H N R P D D C P D C V A S

CCA TTG GGC ACC CGT GTA ACT GTG GTT GAG CAA GGC CAG GCC TCC

P L G T R V T V V E Q G Q A S

TAT TTC TAC GTG GCA TCC TCA CTG GAC GCA GCC GTG GCT GCC AGC

Y F Y V A S S L D A A V A A S

TTC AGC CCA CGC TCA GTG TCT ATC AGG CGT CTC AAG GCT GAC GCC

F S P R S V S I R R L K A D A

TCG GGA TTC GCA CCG GGC TTT GTG GCG TTG TCA GTG CTG CCC AAG

S G F A P G F V A L S V L P K

CAT CTT GTC TCC TAC AGT ATT GAA TAC GTG CAC AGC TTC CAC ACG

H L V S Y S I E Y V H S F H T

GGA GCC TTC GTA TAC TTC CTG ACT GTA CAG CCG GCC AGC GTG ACA

G A F V Y F L T V Q P A S V T

GAT GAT CCT AGT GCC CTG CAC ACA CGC CTG GCA CGG CTT AGC GCC

D D P S A L H T R L A R L S A

ACT GAG CCA GAG TTG GGT GAC TAT CGG GAG CTG GTC CTC GAC TGC

T E P E L G D Y R E L V L D C

AGA TTT GCT CCA AAA CGC AGG CGC CGG GGG GCC CCA GAA GGC GGA

R F A P K R R R R G A P E G G

CAG CCC TAC CCT GTG CTG CGG GTG GCC CAC TCC GCT CCA GTG GGT

Q P Y P V L R V A H S A P V G

GCC CAA CTT GCC ACT GAG CTG AGC ATC GCC GAG GGC CAG GAA GTA

A Q L A T E L S I A E G Q E V

CTA TTT GGG GTC TTT GTG ACT GGC AAG GAT GGT GGT CCT GGC GTG

L F G V F V T G K D G G P G V

GGC CCC AAC TCT GTC GTC TGT GCC TTC CCC ATT GAC CTG CTG GAC

G P N S V V C A F P I D L L D

ACA CTA ATT GAT GAG GGT GTG GAG CGC TGT TGT GAA TCC CCA GTC

T L I D E G V E R C C E S P V

CAT CCA GGC CTC CGG CGA GGC CTC GAC TTC TTC CAG TCG CCC AGT

H P G L R R G L D F F Q S P S

TTT TGC CCC AAC CCG CCT GGC CTG GAA GCC CTC AGC CCC AAC ACC

F C P N P P G L E A L S P N T

AGC TGC CGC CAC TTC CCT CTG CTG GTC AGT AGC AGC TTC TCA CGT

S C R H F P L L V S S S F S R

GTG GAC CTA TTC AAT GGG CTG TTG GGA CCA GTA CAG GTC ACT GCA

V D L F N G L L G P V Q V T A

TTG TAT GTG ACA CGC CTT GAC AAC GTC ACA GTG GCA CAC ATG GGC

L Y V T R L D N V T V A H M G

ACA ATG GAT GGG CGT ATC CTG CAG GTG GGT CCT CAT CCC CAC AGT

T M D G R I L Q V G P H P H S

CCC CTA GCC CTG GGT CCT TGT CTC CAT CCC CAT TTT GCT CAC ATC

P L A L G P C L H P H F A H I

TGA

*

>EU826583,014C01,splice variant of RON,RON-541

ATG GAG CTC CTC CCG CCG CTG CCT CAG TCC TTC CTG TTG CTG CTG

M E L L P P L P Q S F L L L L

CTG TTG CCT GCC AAG CCC GCG GCG GGC GAG GAC TGG CAG TGC CCG

L L P A K P A A G E D W Q C P

CGC ACC CCC TAC GCG GCC TCT CGC GAC TTT GAC GTG AAG TAC GTG

R T P Y A A S R D F D V K Y V

GTG CCC AGC TTC TCC GCC GGA GGC CTG GTA CAG GCC ATG GTG ACC

V P S F S A G G L V Q A M V T

TAC GAG GGC GAC AGA AAT GAG AGT GCT GTG TTT GTA GCC ATA CGC

Y E G D R N E S A V F V A I R

AAT CGC CTG CAT GTG CTT GGG CCT GAC CTG AAG TCT GTC CAG AGC

N R L H V L G P D L K S V Q S

CTG GCC ACG GGC CCT GCT GGA GAC CCT GGC TGC CAG ACG TGT GCA

L A T G P A G D P G C Q T C A

GCC TGT GGC CCA GGA CCC CAC GGC CCT CCC GGT GAC ACA GAC ACA

A C G P G P H G P P G D T D T

AAG GTG CTG GTG CTG GAT CCC GCG CTG CCT GCG CTG GTC AGT TGT

K V L V L D P A L P A L V S C

GGC TCC AGC CTG CAG GGC CGC TGC TTC CTG CAT GAC CTA GAG CCC

G S S L Q G R C F L H D L E P

CAA GGG ACA GCC GTG CAT CTG GCA GCG CCA GCC TGC CTC TTC TCA

Q G T A V H L A A P A C L F S

GCC CAC CAT AAC CGG CCC GAT GAC TGC CCC GAC TGT GTG GCC AGC

A H H N R P D D C P D C V A S

CCA TTG GGC ACC CGT GTA ACT GTG GTT GAG CAA GGC CAG GCC TCC

P L G T R V T V V E Q G Q A S

TAT TTC TAC GTG GCA TCC TCA CTG GAC GCA GCC GTG GCT GCC AGC

Y F Y V A S S L D A A V A A S

TTC AGC CCA CGC TCA GTG TCT ATC AGG CGT CTC AAG GCT GAC GCC

F S P R S V S I R R L K A D A

TCG GGA TTC GCA CCG GGC TTT GTG GCG TTG TCA GTG CTG CCC AAG

S G F A P G F V A L S V L P K

CAT CTT GTC TCC TAC AGT ATT GAA TAC GTG CAC AGC TTC CAC ACG

H L V S Y S I E Y V H S F H T

GGA GCC TTC GTA TAC TTC CTG ACT GTA CAG CCG GCC AGC GTG ACA

G A F V Y F L T V Q P A S V T

GAT GAT CCT AGT GCC CTG CAC ACA CGC CTG GCA CGG CTT AGC GCC

D D P S A L H T R L A R L S A

ACT GAG CCA GAG TTG GGT GAC TAT CGG GAG CTG GTC CTC GAC TGC

T E P E L G D Y R E L V L D C

AGA TTT GCT CCA AAA CGC AGG CGC CGG GGG GCC CCA GAA GGC GGA

R F A P K R R R R G A P E G G

CAG CCC TAC CCT GTG CTG CGG GTG GCC CAC TCC GCT CCA GTG GGT

Q P Y P V L R V A H S A P V G

GCC CAA CTT GCC ACT GAG CTG AGC ATC GCC GAG GGC CAG GAA GTA

A Q L A T E L S I A E G Q E V

CTA TTT GGG GTC TTT GTG ACT GGC AAG GAT GGT GGT CCT GGC GTG

L F G V F V T G K D G G P G V

GGC CCC AAC TCT GTC GTC TGT GCC TTC CCC ATT GAC CTG CTG GAC

G P N S V V C A F P I D L L D

ACA CTA ATT GAT GAG GGT GTG GAG CGC TGT TGT GAA TCC CCA GTC

T L I D E G V E R C C E S P V

CAT CCA GGC CTC CGG CGA GGC CTC GAC TTC TTC CAG TCG CCC AGT

H P G L R R G L D F F Q S P S

TTT TGC CCC AAC CCG GTT TTC CAG GTA CCT ATC CAA GGC CCT GGC

F C P N P V F Q V P I Q G P G

TGC CGC CAC TTC CTG ACC TGT GGG CGT TGC CTA AGG GCA TGG CAT

C R H F L T C G R C L R A W H

TTC ATG GGC TGT GGC TGG TGT GGG AAC ATG TGC GGC CAG CAG AAG

F M G C G W C G N M C G Q Q K

GAG TGT CCT GGC TCC TGG CAA CAG GAC CAC TGC CCA CCT AAG CTT

E C P G S W Q Q D H C P P K L

ACT GAG TTC CAC CCC CAC AGT GGA CCT CTA AGG GGC AGT ACA AGG

T E F H P H S G P L R G S T R

CTG ACC CTG TGT GGC TCC AAC TTC TAC CTT CAC CCT TCT GGT CTG

L T L C G S N F Y L H P S G L

GTG CCT GAG GGA ACC CAT CAG GTC ACT GTG GGC CAA AGT CCC TGC

V P E G T H Q V T V G Q S P C

CGG CCA CTG CCC AAG GAC AGC TCA AAA CTC AGg tAC AAT CTG GTC

R P L P K D S S K L R Y N L V

CCT CCC CTC CCT TTC CCT GAA GGG GGA AAC CAA GCA GCC CCT TCC

P P L P F P E G G N Q A A P S

CCA TGA

P *

>EU826584,014C09,splice variant of RON,RON-908

ATG GAG CTC CTC CCG CCG CTG CCT CAG TCC TTC CTG TTG CTG CTG

M E L L P P L P Q S F L L L L

CTG TTG CCT GCC AAG CCC GCG GCG GGC GAG GAC TGG CAG TGC CCG

L L P A K P A A G E D W Q C P

CGC ACC CCC TAC GCG GCC TCT CGC GAC TTT GAC GTG AAG TAC GTG

R T P Y A A S R D F D V K Y V

GTG CCC AGC TTC TCC GCC GGA GGC CTG GTA CAG GCC ATG GTG ACC

V P S F S A G G L V Q A M V T

TAC GAG GGC GAC AGA AAT GAG AGT GCT GTG TTT GTA GCC ATA CGC

Y E G D R N E S A V F V A I R

AAT CGC CTG CAT GTG CTT GGG CCT GAC CTG AAG TCT GTC CAG AGC

N R L H V L G P D L K S V Q S

CTG GCC ACG GGC CCT GCT GGA GAC CCT GGC TGC CAG ACG TGT GCA

L A T G P A G D P G C Q T C A

GCC TGT GGC CCA GGA CCC CAC GGC CCT CCC GGT GAC ACA GAC ACA

A C G P G P H G P P G D T D T

AAG GTG CTG GTG CTG GAT CCC GCG CTG CCT GCG CTG GTC AGT TGT

K V L V L D P A L P A L V S C

GGC TCC AGC CTG CAG GGC CGC TGC TTC CTG CAT GAC CTA GAG CCC

G S S L Q G R C F L H D L E P

CAA GGG ACA GCC GTG CAT CTG GCA GCG CCA GCC TGC CTC TTC TCA

Q G T A V H L A A P A C L F S

GCC CAC CAT AAC CGG CCC GAT GAC TGC CCC GAC TGT GTG GCC AGC

A H H N R P D D C P D C V A S

CCA TTG GGC ACC CGT GTA ACT GTG GTT GAG CAA GGC CAG GCC TCC

P L G T R V T V V E Q G Q A S

TAT TTC TAC GTG GCA TCC TCA CTG GAC GCA GCC GTG GCT GCC AGC

Y F Y V A S S L D A A V A A S

TTC AGC CCA CGC TCA GTG TCT ATC AGG CGT CTC AAG GCT GAC GCC

F S P R S V S I R R L K A D A

TCG GGA TTC GCA CCG GGC TTT GTG GCG TTG TCA GTG CTG CCC AAG

S G F A P G F V A L S V L P K

CAT CTT GTC TCC TAC AGT ATT GAA TAC GTG CAC AGC TTC CAC ACG

H L V S Y S I E Y V H S F H T

GGA GCC TTC GTA TAC TTC CTG ACT GTA CAG CCG GCC AGC GTG ACA

G A F V Y F L T V Q P A S V T

GAT GAT CCT AGT GCC CTG CAC ACA CGC CTG GCA CGG CTT AGC GCC

D D P S A L H T R L A R L S A

ACT GAG CCA GAG TTG GGT GAC TAT CGG GAG CTG GTC CTC GAC TGC

T E P E L G D Y R E L V L D C

AGA TTT GCT CCA AAA CGC AGG CGC CGG GGG GCC CCA GAA GGC GGA

R F A P K R R R R G A P E G G

CAG CCC TAC CCT GTG CTG CGG GTG GCC CAC TCC GCT CCA GTG GGT

Q P Y P V L R V A H S A P V G

GCC CAA CTT GCC ACT GAG CTG AGC ATC GCC GAG GGC CAG GAA GTA

A Q L A T E L S I A E G Q E V

CTA TTT GGG GTC TTT GTG ACT GGC AAG GAT GGT GGT CCT GGC GTG

L F G V F V T G K D G G P G V

GGC CCC AAC TCT GTC GTC TGT GCC TTC CCC ATT GAC CTG CTG GAC

G P N S V V C A F P I D L L D

ACA CTA ATT GAT GAG GGT GTG GAG CGC TGT TGT GAA TCC CCA GTC

T L I D E G V E R C C E S P V

CAT CCA GGC CTC CGG CGA GGC CTC GAC TTC TTC CAG TCG CCC AGT

H P G L R R G L D F F Q S P S

TTT TGC CCC AAC CCG CCT GGC CTG GAA GCC CTC AGC CCC AAC ACC

F C P N P P G L E A L S P N T

AGC TGC CGC CAC TTC CCT CTG CTG GTC AGT AGC AGC TTC TCA CGT

S C R H F P L L V S S S F S R

GTG GAC CTA TTC AAT GGG CTG TTG GGA CCA GTA CAG GTC ACT GCA

V D L F N G L L G P V Q V T A

TTG TAT GTG ACA CGC CTT GAC AAC GTC ACA GTG GCA CAC ATG GGC

L Y V T R L D N V T V A H M G

ACA ATG GAT GGG CGT ATC CTG CAG GTG GAG CTG GTC AGG TCA CTA

T M D G R I L Q V E L V R S L

AAC TAC TTG CTG TAT GTG TCC AAC TTC TCA CTG GGT GAC AGT GGG

N Y L L Y V S N F S L G D S G

CAG CCC GTG CAG CGG GAT GTC AGT CGT CTT GGG GAC CAC CTA CTC

Q P V Q R D V S R L G D H L L

TTT GCC TCT GGG GAC CAG GTT TTC CAG GTA CCT ATC CAA GGC CCT

F A S G D Q V F Q V P I Q G P

GGC TGC CGC CAC TTC CTG ACC TGT GGG CGT TGC CTA AGG GCA TGG

G C R H F L T C G R C L R A W

CAT TTC ATG GGC TGT GGC TGG TGT GGG AAC ATG TGC GGC CAG CAG

H F M G C G W C G N M C G Q Q

AAG GAG TGT CCT GGC TCC TGG CAA CAG GAC CAC TGC CCA CCT AAG

K E C P G S W Q Q D H C P P K

CTT ACT GAG TTC CAC CCC CAC AGT GGA CCT CTA AGG GGC AGT ACA

L T E F H P H S G P L R G S T

AGG CTG ACC CTG TGT GGC TCC AAC TTC TAC CTT CAC CCT TCT GGT

R L T L C G S N F Y L H P S G

CTG GTG CCT GAG GGA ACC CAT CAG GTC ACT GTG GGC CAA AGT CCC

L V P E G T H Q V T V G Q S P

TGC CGG CCA CTG CCC AAG GAC AGC TCA AAA CTC AGA CCA GTG CCC

C R P L P K D S S K L R P V P

CGG AAA GAC TTT GTA GAG GAG TTT GAG TGT GAA CTG GAG CCC TTG

R K D F V E E F E C E L E P L

GGC ACC CAG GCA GTG GGG CCT ACC AAC GTC AGC CTC ACC GTG ACT

G T Q A V G P T N V S L T V T

AAC ATG CCA CCG GGC AAG CAC TTC CGG GTA GAC GGC ACC TCC GTG

N M P P G K H F R V D G T S V

CTG AGA GGC TTC TCT TTC ATG GAG CCA GTG CTG ATA GCA GTG CAA

L R G F S F M E P V L I A V Q

CCC CTC TTT GGC CCA CGG GCA GGA GGC ACC TGT CTC ACT CTT GAA

P L F G P R A G G T C L T L E

GGC CAG AGT CTG TCT GTA GGC ACC AGC CGG GCT GTG CTG GTC AAT

G Q S L S V G T S R A V L V N

GGG ACT GAG TGT CTG CTA GCA CGG GTC AGT GAG GGG CAG CTT TTA

G T E C L L A R V S E G Q L L

TGT GCC ACA CCC CCT GGG GCC ACG GTG GCC AGT GTC CCC CTT AGC

C A T P P G A T V A S V P L S

CTG CAG GTG GGG GGT GCC CAG GTA CCT GGT TCC TGG ACC TTC CAG

L Q V G G A Q V P G S W T F Q

TAC AGA GAA GAC CCT GTC GTG CTA AGC ATC AGC CCC AAC TGT GGC

Y R E D P V V L S I S P N C G

TAC ATC AAC TCC CAC ATC ACC ATC TGT GGC CAG CAT CTA ACT TCA

Y I N S H I T I C G Q H L T S

GCA TGG CAC TTA GTG CTG TCA TTC CAT GAC GGG CTT AGG GCA GTG

A W H L V L S F H D G L R A V

GAA AGC AGG CAG TGT GAG AGG CAG CTT CCA GAG CAG CAG CTG TGC

E S R Q C E R Q L P E Q Q L C

CGC CTT CCT GAA TAT GTG GTC CGA GAC CCC CAG GGA TGG GTG GCA

R L P E Y V V R D P Q G W V A

GGG AAT CTG AGT GCC CGA GGG GAT GGA GCT GCT GGC TTT ACA CTG

G N L S A R G D G A A G F T L

CCT GGC TTT CGC TTC CTA CCC CCA CCC CAT CCA CCC AGT GCC AAC

P G F R F L P P P H P P S A N

CTA GTT CCA CTG AAG CCT GAG GAG CAT GCC ATT AAG TTT GAG gtA

L V P L K P E E H A I K F E V

AGT GTA AGG GAT AGG GGC AGG GAC AGT TGG GGA TCT GAA AGT AGG

S V R D R G R D S W G S E S R

GGC CAG CCT ACT GGC TGG TCC TCA tga

G Q P T G W S S *

>EU826585,014E12,splice variant of RON,RON-647

ATG GAG CTC CTC CCG CCG CTG CCT CAG TCC TTC CTG TTG CTG CTG

M E L L P P L P Q S F L L L L

CTG TTG CCT GCC AAG CCC GCG GCG GGC GAG GAC TGG CAG TGC CCG

L L P A K P A A G E D W Q C P

CGC ACC CCC TAC GCG GCC TCT CGC GAC TTT GAC GTG AAG TAC GTG

R T P Y A A S R D F D V K Y V

GTG CCC AGC TTC TCC GCC GGA GGC CTG GTA CAG GCC ATG GTG ACC

V P S F S A G G L V Q A M V T

TAC GAG GGC GAC AGA AAT GAG AGT GCT GTG TTT GTA GCC ATA CGC

Y E G D R N E S A V F V A I R

AAT CGC CTG CAT GTG CTT GGG CCT GAC CTG AAG TCT GTC CAG AGC

N R L H V L G P D L K S V Q S

CTG GCC ACG GGC CCT GCT GGA GAC CCT GGC TGC CAG ACG TGT GCA

L A T G P A G D P G C Q T C A

GCC TGT GGC CCA GGA CCC CAC GGC CCT CCC GGT GAC ACA GAC ACA

A C G P G P H G P P G D T D T

AAG GTG CTG GTG CTG GAT CCC GCG CTG CCT GCG CTG GTC AGT TGT

K V L V L D P A L P A L V S C

GGC TCC AGC CTG CAG GGC CGC TGC TTC CTG CAT GAC CTA GAG CCC

G S S L Q G R C F L H D L E P

CAA GGG ACA GCC GTG CAT CTG GCA GCG CCA GCC TGC CTC TTC TCA

Q G T A V H L A A P A C L F S

GCC CAC CAT AAC CGG CCC GAT GAC TGC CCC GAC TGT GTG GCC AGC

A H H N R P D D C P D C V A S

CCA TTG GGC ACC CGT GTA ACT GTG GTT GAG CAA GGC CAG GCC TCC

P L G T R V T V V E Q G Q A S

TAT TTC TAC GTG GCA TCC TCA CTG GAC GCA GCC GTG GCT GCC AGC

Y F Y V A S S L D A A V A A S

TTC AGC CCA CGC TCA GTG TCT ATC AGG CGT CTC AAG GCT GAC GCC

F S P R S V S I R R L K A D A

TCG GGA TTC GCA CCG GGC TTT GTG GCG TTG TCA GTG CTG CCC AAG

S G F A P G F V A L S V L P K

CAT CTT GTC TCC TAC AGT ATT GAA TAC GTG CAC AGC TTC CAC ACG

H L V S Y S I E Y V H S F H T

GGA GCC TTC GTA TAC TTC CTG ACT GTA CAG CCG GCC AGC GTG ACA

G A F V Y F L T V Q P A S V T

GAT GAT CCT AGT GCC CTG CAC ACA CGC CTG GCA CGG CTT AGC GCC

D D P S A L H T R L A R L S A

ACT GAG CCA GAG TTG GGT GAC TAT CGG GAG CTG GTC CTC GAC TGC

T E P E L G D Y R E L V L D C

AGA TTT GCT CCA AAA CGC AGG CGC CGG GGG GCC CCA GAA GGC GGA

R F A P K R R R R G A P E G G

CAG CCC TAC CCT GTG CTG CGG GTG GCC CAC TCC GCT CCA GTG GGT

Q P Y P V L R V A H S A P V G

GCC CAA CTT GCC ACT GAG CTG AGC ATC GCC GAG GGC CAG GAA GTA

A Q L A T E L S I A E G Q E V

CTA TTT GGG GTC TTT GTG ACT GGC AAG GAT GGT GGT CCT GGC GTG

L F G V F V T G K D G G P G V

GGC CCC AAC TCT GTC GTC TGT GCC TTC CCC ATT GAC CTG CTG GAC

G P N S V V C A F P I D L L D

ACA CTA ATT GAT GAG GGT GTG GAG CGC TGT TGT GAA TCC CCA GTC

T L I D E G V E R C C E S P V

CAT CCA GGC CTC CGG CGA GGC CTC GAC TTC TTC CAG TCG CCC AGT

H P G L R R G L D F F Q S P S

TTT TGC CCC AAC CCG CCT GGC CTG GAA GCC CTC AGC CCC AAC ACC

F C P N P P G L E A L S P N T

AGC TGC CGC CAC TTC CCT CTG CTG GTC AGT AGC AGC TTC TCA CGT

S C R H F P L L V S S S F S R

GTG GAC CTA TTC AAT GGG CTG TTG GGA CCA GTA CAG GTC ACT GCA

V D L F N G L L G P V Q V T A

TTG TAT GTG ACA CGC CTT GAC AAC GTC ACA GTG GCA CAC ATG GGC

L Y V T R L D N V T V A H M G

ACA ATG GAT GGG CGT ATC CTG CAG GTG GAG CTG GTC AGG TCA CTA

T M D G R I L Q V E L V R S L

AAC TAC TTG CTG TAT GTG TCC AAC TTC TCA CTG GGT GAC AGT GGG

N Y L L Y V S N F S L G D S G

CAG CCC GTG CAG CGG GAT GTC AGT CGT CTT GGG GAC CAC CTA CTC

Q P V Q R D V S R L G D H L L

TTT GCC TCT GGG GAC CAG GTT TTC CAG GTA CCT ATC CAA GGC CCT

F A S G D Q V F Q V P I Q G P

GGC TGC CGC CAC TTC CTG ACC TGT GGG CGT TGC CTA AGG GCA TGG

G C R H F L T C G R C L R A W

CAT TTC ATG GGC TGT GGC TGG TGT GGG AAC ATG TGC GGC CAG CAG

H F M G C G W C G N M C G Q Q

AAG GAG TGT CCT GGC TCC TGG CAA CAG GAC CAC TGC CCA CCT AAG

K E C P G S W Q Q D H C P P K

CTT ACT GAG TTC CAC CCC CAC AGT GGA CCT CTA AGG GGC AGT ACA

L T E F H P H S G P L R G S T

AGG CTG ACC CTG TGT GGC TCC AAC TTC TAC CTT CAC CCT TCT GGT

R L T L C G S N F Y L H P S G

CTG GTG CCT GAG GGA ACC CAT CAG GTC ACT GTG GGC CAA AGT CCC

L V P E G T H Q V T V G Q S P

TGC CGG CCA CTG CCC AAG GAC AGC TCA AAA CTC AGg tAC AAT CTG

C R P L P K D S S K L R Y N L

GTC CCT CCC CTC CCT TTC CCT GAA GGG GGA AAC CAA GCA GCC CCT

V P P L P F P E G G N Q A A P

TCC CCA TGA

S P *

>EU826586,006A04,splice variant of TIE1,TIE1-251

ATG GTC TGG CGG GTG CCC CCT TTC TTG CTC CCC ATC CTC TTC TTG

M V W R V P P F L L P I L F L

GCT TCT CAT GTG GGC GCG GCG GTG GAC CTG ACG CTG CTG GCC AAC

A S H V G A A V D L T L L A N

CTG CGG CTC ACG GAC CCC CAG CGC TTC TTC CTG ACT TGC GTG TCT

L R L T D P Q R F F L T C V S

GGG GAG GCC GGG GCG GGG AGG GGC TCG GAC GCC TGG GGC CCG CCC

G E A G A G R G S D A W G P P

CTG CTG CTG GAG AAG GAC GAC CGT ATC GTG CGC ACC CCG CCC GGG

L L L E K D D R I V R T P P G

CCA CCC CTG CGC CTG GCG CGC AAC GGT TCG CAC CAG GTC ACG CTT

P P L R L A R N G S H Q V T L

CGC GGC TTC TCC AAG CCC TCG GAC CTC GTG GGC GTC TTC TCC TGC

R G F S K P S D L V G V F S C

GTG GGC GGT GCT GGG GCG CGG CGC ACG CGC GTC ATC TAC GTG CAC

V G G A G A R R T R V I Y V H

AAC AGC CCT GGA GCC CAC CTG CTT CCA GAC AAG GTC ACA CAC ACT

N S P G A H L L P D K V T H T

GTG AAC AAA GGT GAC ACC GCT GTA CTT TCT GCA CGT GTG CAC AAG

V N K G D T A V L S A R V H K

GAG AAG CAG ACA GAC GTG ATC TGG AAG AGC AAC GGA TCC TAC TTC

E K Q T D V I W K S N G S Y F

TAC ACC CTG GAC TGG CAT GAA GCC CAG GAT GGG CGG TTC CTG CTG

Y T L D W H E A Q D G R F L L

CAG CTC CCA AAT GTG CAG CCA CCA TCG AGC GGC ATC TAC AGT GCC

Q L P N V Q P P S S G I Y S A

ACT TAC CTG GAA GCC AGC CCC CTG GGC AGC GCC TTC TTT CGG CTC

T Y L E A S P L G S A F F R L

ATC GTG CGG GGT CAG AGG CAG AGG GCA GAG GTT GTG GGT AGG GTG

I V R G Q R Q R A E V V G R V

GGA GGC TGG GAG CCC TAT GGG TAC TTC CTG TGG GTC CCC TGG AGC

G G W E P Y G Y F L W V P W S

CCC TGG ATC TCC AGT CCT CAG TGG TCA GGT GGG TGA

P W I S S P Q W S G G *

>EU826587,006B07,splice variant of TIE1,TIE1-379

ATG GTC TGG CGG GTG CCC CCT TTC TTG CTC CCC ATC CTC TTC TTG

M V W R V P P F L L P I L F L

GCT TCT CAT GTG GGC GCG GCG GTG GAC CTG ACG CTG CTG GCC AAC

A S H V G A A V D L T L L A N

CTG CGG CTC ACG GAC CCC CAG CGC TTC TTC CTG ACT TGC GTG TCT

L R L T D P Q R F F L T C V S

GGG GAG GCC GGG GCG GGG AGG GGC TCG GAC GCC TGG GGC CCG CCC

G E A G A G R G S D A W G P P

CTG CTG CTG GAG AAG GAC GAC CGT ATC GTG CGC ACC CCG CCC GGG

L L L E K D D R I V R T P P G

CCA CCC CTG CGC CTG GCG CGC AAC GGT TCG CAC CAG GTC ACG CTT

P P L R L A R N G S H Q V T L

CGC GGC TTC TCC AAG CCC TCG GAC CTC GTG GGC GTC TTC TCC TGC

R G F S K P S D L V G V F S C

GTG GGC GGT GCT GGG GCG CGG CGC ACG CGC GTC ATC TAC GTG CAC

V G G A G A R R T R V I Y V H

AAC AGC CCT GGA GCC CAC CTG CTT CCA GAC AAG GTC ACA CAC ACT

N S P G A H L L P D K V T H T

GTG AAC AAA GGT GAC ACC GCT GTA CTT TCT GCA CGT GTG CAC AAG

V N K G D T A V L S A R V H K

GAG AAG CAG ACA GAC GTG ATC TGG AAG AGC AAC GGA TCC TAC TTC

E K Q T D V I W K S N G S Y F

TAC ACC CTG GAC TGG CAT GAA GCC CAG GAT GGG CGG TTC CTG CTG

Y T L D W H E A Q D G R F L L

CAG CTC CCA AAT GTG CAG CCA CCA TCG AGC GGC ATC TAC AGT GCC

Q L P N V Q P P S S G I Y S A

ACT TAC CTG GAA GCC AGC CCC CTG GGC AGC GCC TTC TTT CGG CTC

T Y L E A S P L G S A F F R L

ATC GTG CGG GGT TGT GGG GCT GGG CGC TGG GGG CCA GGC TGT ACC

I V R G C G A G R W G P G C T

AAG GAG TGC CCA GGT TGC CTA CAT GGA GGT GTC TGC CAC GAC CAT

K E C P G C L H G G V C H D H

GAC GGC GAA TGT GTA TGC CCC CCT GGC TTC ACT GGC ACC CGC TGT

D G E C V C P P G F T G T R C

GAA CAG GCC TGC AGA GAG GGC CGT TTT GGG CAG AGC TGC CAG GAG

E Q A C R E G R F G Q S C Q E

CAG TGC CCA GGC ATA TCA GGC TGC CGG GGC CTC ACC TTC TGC CTC

Q C P G I S G C R G L T F C L

CCA GAC CCC TAT GGC TGC TCT TGT GGA TCT GGC TGG AGA GGA AGC

P D P Y G C S C G S G W R G S

CAG TGC CAA GAA GCT TGT GCC CCT GGT CAT TTT GGG GCT GAT TGC

Q C Q E A C A P G H F G A D C

CGA CTC CAG TGC CAG TGT CAG AAT GGT GGC ACT TGT GAC CGG TTC

R L Q C Q C Q N G G T C D R F

AGT GGT TGT GTC TGC CCC TCT GGG TGG CAT GGA GTG CAC TGT GAG

S G C V C P S G W H G V H C E

AAG TCA GGC TGG AGG GAC TGG GTA GAT ACC TCC ACT GAG AAA CAG

K S G W R D W V D T S T E K Q

AAC ACG GAT GAG GGG CGC TTT GGT GGT CAC GTG TCT GCC CCT GTG

N T D E G R F G G H V S A P V

GGA GCT CCA GGA TGA

G A P G *

>EU826588,006B10,splice variant of TIE1,TIE1-317

ATG GTC TGG CGG GTG CCC CCT TTC TTG CTC CCC ATC CTC TTC TTG

M V W R V P P F L L P I L F L

GCT TCT CAT GTG GGC GCG GCG GTG GAC CTG ACG CTG CTG GCC AAC

A S H V G A A V D L T L L A N

CTG CGG CTC ACG GAC CCC CAG CGC TTC TTC CTG ACT TGC GTG TCT

L R L T D P Q R F F L T C V S

GGG GAG GCC GGG GCG GGG AGG GGC TCG GAC GCC TGG GGC CCG CCC

G E A G A G R G S D A W G P P

CTG CTG CTG GAG AAG GAC GAC CGT ATC GTG CGC ACC CCG CCC GGG

L L L E K D D R I V R T P P G

CCA CCC CTG CGC CTG GCG CGC AAC GGT TCG CAC CAG GTC ACG CTT

P P L R L A R N G S H Q V T L

CGC GGC TTC TCC AAG CCC TCG GAC CTC GTG GGC GTC TTC TCC TGC

R G F S K P S D L V G V F S C

GTG GGC GGT GCT GGG GCG CGG CGC ACG CGC GTC ATC TAC GTG CAC

V G G A G A R R T R V I Y V H

AAC AGC CCT GGA GCC CAC CTG CTT CCA GAC AAG GTC ACA CAC ACT

N S P G A H L L P D K V T H T

GTG AAC AAA GGT GAC ACC GCT GTA CTT TCT GCA CGT GTG CAC AAG

V N K G D T A V L S A R V H K

GAG AAG CAG ACA GAC GTG ATC TGG AAG AGC AAC GGA TCC TAC TTC

E K Q T D V I W K S N G S Y F

TAC ACC CTG GAC TGG CAT GAA GCC CAG GAT GGG CGG TTC CTG CTG

Y T L D W H E A Q D G R F L L

CAG CTC CCA AAT GTG CAG CCA CCA TCG AGC GGC ATC TAC AGT GCC

Q L P N V Q P P S S G I Y S A

ACT TAC CTG GAA GCC AGC CCC CTG GGC AGC GCC TTC TTT CGG CTC

T Y L E A S P L G S A F F R L

ATC GTG CGG GGT TGT GGG GCT GGG CGC TGG GGG CCA GGC TGT ACC

I V R G C G A G R W G P G C T

AAG GAG TGC CCA GGT TGC CTA CAT GGA GGT GTC TGC CAC GAC CAT

K E C P G C L H G G V C H D H

GAC GGC GAA TGT GTA TGC CCC CCT GGC TTC ACT GGC ACC CGC TGT

D G E C V C P P G F T G T R C

GAA CAG GCC TGC AGA GAG GGC CGT TTT GGG CAG AGC TGC CAG GAG

E Q A C R E G R F G Q S C Q E

CAG TGC CCA GGC ATA TCA GGC TGC CGG GGC CTC ACC TTC TGC CTC

Q C P G I S G C R G L T F C L

CCA GAC CCC TAT GGC TGC TCT TGT GGA TCT GGC TGG AGA GGA AGC

P D P Y G C S C G S G W R G S

CAG TGC CAA GAA GTC CAC CAA GGC CAT TGT GGA GCC AGA GAA GAC

Q C Q E V H Q G H C G A R E D

CAC AGC TGA

H S *

>EU826589,006B06,splice variant of TIE1,TIE1-161

ATG GTC TGG CGG GTG CCC CCT TTC TTG CTC CCC ATC CTC TTC TTG

M V W R V P P F L L P I L F L

GCT TCT CAT GTG GGC GCG GCG GTG GAC CTG ACG CTG CTG GCC AAC

A S H V G A A V D L T L L A N

CTG CGG CTC ACG GAC CCC CAG CGC TTC TTC CTG ACT TGC GTG TCT

L R L T D P Q R F F L T C V S

GGG GAG GCC GGG GCG GGG AGG GGC TCG GAC GCC TGG GGC CCG CCC

G E A G A G R G S D A W G P P

CTG CTG CTG GAG AAG GAC GAC CGT ATC GTG CGC ACC CCG CCC GGG

L L L E K D D R I V R T P P G

CCA CCC CTG CGC CTG GCG CGC AAC GGT TCG CAC CAG GTC ACG CTT

P P L R L A R N G S H Q V T L

CGC GGC TTC TCC AAG CCC TCG GAC CTC GTG GGC GTC TTC TCC TGC

R G F S K P S D L V G V F S C

GTG GGC GGT GCT GGG GCG CGG CGC ACG CGC GTC ATC TAC GTG CAC

V G G A G A R R T R V I Y V H

AAC AGC CCT GGA GGT GAG TTA GGC AGG CGG GGG GAT GGC GCG GGG

N S P G G E L G R R G D G A G

AAA ACC AGG CCG CTG ACC CAC CTT CCA CCC CGC AGC CCA CCT GCT

K T R P L T H L P P R S P P A

TCC AGA CAA GGT CAC ACA CAC TGT GAg CAA AGG TGA

S R Q G H T H C E Q R *

>EU826590,016G03,splice variant of TIE1,TIE1-751

ATG GTC TGG CGG GTG CCC CCT TTC TTG CTC CCC ATC CTC TTC TTG

M V W R V P P F L L P I L F L

GCT TCT CAT GTG GGC GCG GCG GTG GAC CTG ACG CTG CTG GCC AAC

A S H V G A A V D L T L L A N

CTG CGG CTC ACG GAC CCC CAG CGC TTC TTC CTG ACT TGC GTG TCT

L R L T D P Q R F F L T C V S

GGG GAG GCC GGG GCG GGG AGG GGC TCG GAC GCC TGG GGC CCG CCC

G E A G A G R G S D A W G P P

CTG CTG CTG GAG AAG GAC GAC CGT ATC GTG CGC ACC CCG CCC GGG

L L L E K D D R I V R T P P G

CCA CCC CTG CGC CTG GCG CGC AAC GGT TCG CAC CAG GTC ACG CTT

P P L R L A R N G S H Q V T L

CGC GGC TTC TCC AAG CCC TCG GAC CTC GTG GGC GTC TTC TCC TGC

R G F S K P S D L V G V F S C

GTG GGC GGT GCT GGG GCG CGG CGC ACG CGC GTC ATC TAC GTG CAC

V G G A G A R R T R V I Y V H

AAC AGC CCT GGA GCC CAC CTG CTT CCA GAC AAG GTC ACA CAC ACT

N S P G A H L L P D K V T H T

GTG AAC AAA GGT GAC ACC GCT GTA CTT TCT GCA CGT GTG CAC AAG

V N K G D T A V L S A R V H K

GAG AAG CAG ACA GAC GTG ATC TGG AAG AGC AAC GGA TCC TAC TTC

E K Q T D V I W K S N G S Y F

TAC ACC CTG GAC TGG CAT GAA GCC CAG GAT GGG CGG TTC CTG CTG

Y T L D W H E A Q D G R F L L

CAG CTC CCA AAT GTG CAG CCA CCA TCG AGC GGC ATC TAC AGT GCC

Q L P N V Q P P S S G I Y S A

ACT TAC CTG GAA GCC AGC CCC CTG GGC AGC GCC TTC TTT CGG CTC

T Y L E A S P L G S A F F R L

ATC GTG CGG GGT TGT GGG GCT GGG CGC TGG GGG CCA GGC TGT ACC

I V R G C G A G R W G P G C T

AAG GAG TGC CCA GGT TGC CTA CAT GGA GGT GTC TGC CAC GAC CAT

K E C P G C L H G G V C H D H

GAC GGC GAA TGT GTA TGC CCC CCT GGC TTC ACT GGC ACC CGC TGT

D G E C V C P P G F T G T R C

GAA CAG GCC TGC AGA GAG GGC CGT TTT GGG CAG AGC TGC CAG GAG

E Q A C R E G R F G Q S C Q E

CAG TGC CCA GGC ATA TCA GGC TGC CGG GGC CTC ACC TTC TGC CTC

Q C P G I S G C R G L T F C L

CCA GAC CCC TAT GGC TGC TCT TGT GGA TCT GGC TGG AGA GGA AGC

P D P Y G C S C G S G W R G S

CAG TGC CAA GAA GCT TGT GCC CCT GGT CAT TTT GGG GCT GAT TGC

Q C Q E A C A P G H F G A D C

CGA CTC CAG TGC CAG TGT CAG AAT GGT GGC ACT TGT GAC CGG TTC

R L Q C Q C Q N G G T C D R F

AGT GGT TGT GTC TGC CCC TCT GGG TGG CAT GGA GTG CAC TGT GAG

S G C V C P S G W H G V H C E

AAG TCA GAC CGG ATC CCC CAG ATC CTC AAC ATG GCC TCA GAA CTG

K S D R I P Q I L N M A S E L

GAG TTC AAC TTA GAG ACG ATG CCC CGG ATC AAC TGT GCA GCT GCA

E F N L E T M P R I N C A A A

GGG AAC CCC TTC CCC GTG CGG GGC AGC ATA GAG CTA CGC AAG CCA

G N P F P V R G S I E L R K P

GAC GGC ACT GTG CTC CTG TCC ACC AAG GCC ATT GTG GAG CCA GAG

D G T V L L S T K A I V E P E

AAG ACC ACA GCT GAG TTC GAG GTG CCC CGC TTG GTT CTT GCG GAC

K T T A E F E V P R L V L A D

AGT GGG TTC TGG GAG TGC CGT GTG TCC ACA TCT GGC GGC CAA GAC

S G F W E C R V S T S G G Q D

AGC CGG CGC TTC AAG GTC AAT GTG AAA GTG CCC CCC GTG CCC CTG

S R R F K V N V K V P P V P L

GCT GCA CCT CGG CTC CTG ACC AAG CAG AGC CGC CAG CTT GTG GTC

A A P R L L T K Q S R Q L V V

TCC CCG CTG GTC TCG TTC TCT GGG GAT GGA CCC ATC TCC ACT GTC

S P L V S F S G D G P I S T V

CGC CTG CAC TAC CGG CCC CAG GAC AGT ACC ATG GAC TGG TCG ACC

R L H Y R P Q D S T M D W S T

ATT GTG GTG GAC CCC AGT GAG AAC GTG ACG TTA ATG AAC CTG AGG

I V V D P S E N V T L M N L R

CCA AAG ACA GGA TAC AGT GTT CGT GTG CAG CTG AGC CGG CCA GGG

P K T G Y S V R V Q L S R P G

GAA GGA GGA GAG GGG GCC TGG GGG CCT CCC ACC CTC ATG ACC ACA

E G G E G A W G P P T L M T T

GAC TGT CCT GAG CCT TTG TTG CAG CCG TGG TTG GAG GGC TGG CAT

D C P E P L L Q P W L E G W H

GTG GAA GGC ACT GAC CGG CTG CGA GTG AGC TGG TCC TTG CCC TTG

V E G T D R L R V S W S L P L

GTG CCC GGG CCA CTG GTG GGC GAC GGT TTC CTG CTG CGC CTG TGG

V P G P L V G D G F L L R L W

GAC GGG ACA CGG GGG CAG GAG CGG CGG GAG AAC GTC TCA TCC CCC

D G T R G Q E R R E N V S S P

CAG GCC CGC ACT GCC CTC CTG ACG GGA CTC ACG CCT GGC ACC CAC

Q A R T A L L T G L T P G T H

TAC CAG CTG GAT GTG CAG CTC TAC CAC TGC ACC CTC CTG GGC CCG

Y Q L D V Q L Y H C T L L G P

GCC TCG CCC CCT GCA CAC GTG CTT CTG CCC CCC AGT GGG CCT CCA

A S P P A H V L L P P S G P P

GCC CCC CGA CAC CTC CAC GCC CAG GCC CTC TCA GAC TCC GAG ATC

A P R H L H A Q A L S D S E I

CAG CTG ACA TGG AAG CAC CCG GAG GCT CTG CCT GGG CCA ATA TCC

Q L T W K H P E A L P G P I S

AAG TAC GTT GTG GAG GTG CAG GTG GCT GGG GGT GCA GGA GAC CCA

K Y V V E V Q V A G G A G D P

CTG TGG ATA GAC GTG GAC AGG CCT GAG GAG ACA AGC ACC ATC ATC

L W I D V D R P E E T S T I I

CGT GGC CTC AAC GCC AGC ACG CGC TAC CTC TTC CGC ATG CGG GCC

R G L N A S T R Y L F R M R A

AGC ATT CAG GGG CTC GGG GAC TGG AGC AAC ACA GTA GAA GAG TCC

S I Q G L G D W S N T V E E S

ACC CTG GGC AAC GGT GAG AGG GCA GGG CCC ACA GGA CCC CCC GGG

T L G N G E R A G P T G P P G

CTC TGA

L *

>EU826591,007G02,splice variant of TIE2,TIE2-367

ATG GAC TCT TTA GCC AGC TTA GTT CTC TGT GGA GTC AGC TTG CTC

M D S L A S L V L C G V S L L

CTT TCT GGA ACT GTG GAA GGT GCC ATG GAC TTG ATC TTG ATC AAT

L S G T V E G A M D L I L I N

TCC CTA CCT CTT GTA TCT GAT GCT GAA ACA TCT CTC ACC TGC ATT

S L P L V S D A E T S L T C I

GCC TCT GGG TGG CGC CCC CAT GAG CCC ATC ACC ATA GGA AGG GAC

A S G W R P H E P I T I G R D

TTT GAA GCC TTA ATG AAC CAG CAC CAG GAT CCG CTG GAA GTT ACT

F E A L M N Q H Q D P L E V T

CAA GAT GTG ACC AGA GAA TGG GCT AAA AAA GTT GTT TGG AAG AGA

Q D V T R E W A K K V V W K R

GAA AAG GCT AGT AAG ATC AAT GGT GCT TAT TTC TGT GAA GGG CGA

E K A S K I N G A Y F C E G R

GTT CGA GGA GAG GCA ATC AGG ATA CGA ACC ATG AAG ATG CGT CAA

V R G E A I R I R T M K M R Q

CAA GCT TCC TTC CTA CCA GCT ACT TTA ACT ATG ACT GTG GAC AAG

Q A S F L P A T L T M T V D K

GGA GAT AAC GTG AAC ATA TCT TTC AAA AAG GTA TTG ATT AAA GAA

G D N V N I S F K K V L I K E

GAA GAT GCA GTG ATT TAC AAA AAT GGT TCC TTC ATC CAT TCA GTG

E D A V I Y K N G S F I H S V

CCC CGG CAT GAA GTA CCT GAT ATT CTA GAA GTA CAC CTG CCT CAT

P R H E V P D I L E V H L P H

GCT CAG CCC CAG GAT GCT GGA GTG TAC TCG GCC AGG TAT ATA GGA

A Q P Q D A G V Y S A R Y I G

GGA AAC CTC TTC ACC TCG GCC TTC ACC AGG CTG ATA GTC CGG AGA

G N L F T S A F T R L I V R R

TGT GAA GCC CAG AAG TGG GGA CCT GAA TGC AAC CAT CTC TGT ACT

C E A Q K W G P E C N H L C T

GCT TGT ATG AAC AAT GGT GTC TGC CAT GAA GAT ACT GGA GAA TGC

A C M N N G V C H E D T G E C

ATT TGC CCT CCT GGG TTT ATG GGA AGG ACG TGT GAG AAG GCT TGT

I C P P G F M G R T C E K A C

GAA CTG CAC ACG TTT GGC AGA ACT TGT AAA GAA AGG TGC AGT GGA

E L H T F G R T C K E R C S G

CAA GAG GGA TGC AAG TCT TAT GTG TTC TGT CTC CCT GAC CCC TAT

Q E G C K S Y V F C L P D P Y

GGG TGT TCC TGT GCC ACA GGC TGG AAG GGT CTG CAG TGC AAT GAA

G C S C A T G W K G L Q C N E

GCA TGC CAC CCT GGT TTT TAC GGG CCA GAT TGT AAG CTT AGG TGC

A C H P G F Y G P D C K L R C

AGC TGC AAC AAT GGG GAG ATG TGT GAT CGC TTC CAA GGA TGT CTC

S C N N G E M C D R F Q G C L

TGC TCT CCA GGA TGG CAG GGG CTC CAG TGT GAG AGA GAA GGT AAA

C S P G W Q G L Q C E R E G K

GCA AGG CAT ACA GAG GAT GAC CCC AAA GAT AGT GGA TTT GCC AGA

A R H T E D D P K D S G F A R

TCA TAT AGA AGT AAA CAG TGG TAA

S Y R S K Q W *

>EU826592,007H03,splice variant of TIE2,TIE2-468

ATG GAC TCT TTA GCC AGC TTA GTT CTC TGT GGA GTC AGC TTG CTC

M D S L A S L V L C G V S L L

CTT TCT GGA ACT GTG GAA GGT GCC ATG GAC TTG ATC TTG ATC AAT

L S G T V E G A M D L I L I N

TCC CTA CCT CTT GTA TCT GAT GCT GAA ACA TCT CTC ACC TGC ATT

S L P L V S D A E T S L T C I

GCC TCT GGG TGG CGC CCC CAT GAG CCC ATC ACC ATA GGA AGG GAC

A S G W R P H E P I T I G R D

TTT GAA GCC TTA ATG AAC CAG CAC CAG GAT CCG CTG GAA GTT ACT

F E A L M N Q H Q D P L E V T

CAA GAT GTG ACC AGA GAA TGG GCT AAA AAA GTT GTT TGG AAG AGA

Q D V T R E W A K K V V W K R

GAA AAG GCT AGT AAG ATC AAT GGT GCT TAT TTC TGT GAA GGG CGA

E K A S K I N G A Y F C E G R

GTT CGA GGA GAG GCA ATC AGG ATA CGA ACC ATG AAG ATG CGT CAA

V R G E A I R I R T M K M R Q

CAA GCT TCC TTC CTA CCA GCT ACT TTA ACT ATG ACT GTG GAC AAG

Q A S F L P A T L T M T V D K

GGA GAT AAC GTG AAC ATA TCT TTC AAA AAG GTA TTG ATT AAA GAA

G D N V N I S F K K V L I K E

GAA GAT GCA GTG ATT TAC AAA AAT GGT TCC TTC ATC CAT TCA GTG

E D A V I Y K N G S F I H S V

CCC CGG CAT GAA GTA CCT GAT ATT CTA GAA GTA CAC CTG CCT CAT

P R H E V P D I L E V H L P H

GCT CAG CCC CAG GAT GCT GGA GTG TAC TCG GCC AGG TAT ATA GGA

A Q P Q D A G V Y S A R Y I G

GGA AAC CTC TTC ACC TCG GCC TTC ACC AGG CTG ATA GTC CGG AGA

G N L F T S A F T R L I V R R

TGT GAA GCC CAG AAG TGG GGA CCT GAA TGC AAC CAT CTC TGT ACT

C E A Q K W G P E C N H L C T

GCT TGT ATG AAC AAT GGT GTC TGC CAT GAA GAT ACT GGA GAA TGC

A C M N N G V C H E D T G E C

ATT TGC CCT CCT GGG TTT ATG GGA AGG ACG TGT GAG AAG GCT TGT

I C P P G F M G R T C E K A C

GAA CTG CAC ACG TTT GGC AGA ACT TGT AAA GAA AGG TGC AGT GGA

E L H T F G R T C K E R C S G

CAA GAG GGA TGC AAG TCT TAT GTG TTC TGT CTC CCT GAC CCC TAT

Q E G C K S Y V F C L P D P Y

GGG TGT TCC TGT GCC ACA GGC TGG AAG GGT CTG CAG TGC AAT GAA

G C S C A T G W K G L Q C N E

GGC ATA CAG AGG ATG ACC CCA AAG ATA GTG GAT TTG CCA GAT CAT

G I Q R M T P K I V D L P D H

ATA GAA GTA AAC AGT GGT AAA TTT AAT CCC ATT TGC AAA GCT TCT

I E V N S G K F N P I C K A S

GGC TGG CCG CTA CCT ACT AAT GAA GAA ATG ACC CTG GTG AAG CCG

G W P L P T N E E M T L V K P

GAT GGG ACA GTG CTC CAT CCA AAA GAC TTT AAC CAT ACG GAT CAT

D G T V L H P K D F N H T D H

TTC TCA GTA GCC ATA TTC ACC ATC CAC CGG ATC CTC CCC CCT GAC

F S V A I F T I H R I L P P D

TCA GGA GTT TGG GTC TGC AGT GTG AAC ACA GTG GCT GGG ATG GTG

S G V W V C S V N T V A G M V

GAA AAG CCC TTC AAC ATT TCT GTT AAA GTT CTT CCA AAG CCC CTG

E K P F N I S V K V L P K P L

AAT GCC CCA AAC GTG ATT GAC ACT GGA CAT AAC TTT GCT GTC ATC

N A P N V I D T G H N F A V I

AAC ATC AGC TCT GAG CCT TAC TTT GGG GAT GGA CCA ATC AAA TCC

N I S S E P Y F G D G P I K S

AAG AAG CTT CTA TAC AAA CCC GTT AAT CAC TAT GAG GCT TGG CAA

K K L L Y K P V N H Y E A W Q

CAT ATT CAA GTA TCC ATG GAG AAA CAG AGG CTG ACT AAA GCA AAT

H I Q V S M E K Q R L T K A N

AGT GAC AAA TGA

S D K *

>EU826593,005A06,splice variant of CSF1R,CSF1R-306

ATG GGC CCA GGA GTT CTG CTG CTC CTG CTG GTG GCC ACA GCT TGG

M G P G V L L L L L V A T A W

CAT GGT CAG GGA ATC CCA GTG ATA GAG CCC AGT GTC CCT GAG CTG

H G Q G I P V I E P S V P E L

GTC GTG AAG CCA GGA GCA ACG GTG ACC TTG CGA TGT GTG GGC AAT

V V K P G A T V T L R C V G N

GGC AGC GTG GAA TGG GAT GGC CCC CCA TCA CCT CAC TGG ACC CTG

G S V E W D G P P S P H W T L

TAC TCT GAT GGC TCC AGC AGC ATC CTC AGC ACC AAC AAC GCT ACC

Y S D G S S S I L S T N N A T

TTC CAA AAC ACG GGG ACC TAT CGC TGC ACT GAG CCT GGA GAC CCC

F Q N T G T Y R C T E P G D P

CTG GGA GGC AGC GCC GCC ATC CAC CTC TAT GTC AAA GAC CCT GCC

L G G S A A I H L Y V K D P A

CGG CCC TGG AAC GTG CTA GCA CAG GAG GTG GTC GTG TTC GAG GAC

R P W N V L A Q E V V V F E D

CAG GAC GCA CTA CTG CCC TGT CTG CTC ACA GAC CCG GTG CTG GAA

Q D A L L P C L L T D P V L E

GCA GGC GTC TCG CTG GTG CGT GTG CGT GGC CGG CCC CTC ATG CGC

A G V S L V R V R G R P L M R

CAC ACC AAC TAC TCC TTC TCG CCC TGG CAT GGC TTC ACC ATC CAC

H T N Y S F S P W H G F T I H

AGG GCC AAG TTC ATT CAG AGC CAG GAC TAT CAA TGC AGT GCC CTG

R A K F I Q S Q D Y Q C S A L

ATG GGT GGC AGG AAG GTG ATG TCC ATC AGC ATC CGG CTG AAA GTG

M G G R K V M S I S I R L K V

CAG AAA GTC ATC CCA GGG CCC CCA GCC TTG ACA CTG GTG CCT GCA

Q K V I P G P P A L T L V P A

GAG CTG GTG CGG ATT CGA GGG GAG GCT GCC CAG ATC GTG TGC TCA

E L V R I R G E A A Q I V C S

GCC AGC AGC GTT GAT GTT AAC TTT GAT GTC TTC CTC CAA CAC AAC

A S S V D V N F D V F L Q H N

AAC ACC AAG CTC GCA ATC CCT CAA CAA TCT GAC TTT CAT AAT AAC

N T K L A I P Q Q S D F H N N

CGT TAC CAA AAA GTC CTG ACC CTC AAC CTC GAT CAA GTA GAT TTC

R Y Q K V L T L N L D Q V D F

CAA CAT GCC GGC AAC TAC TCC TGC GTG GCC AGC AAC GTG CAG GGC

Q H A G N Y S C V A S N V Q G

AAG CAC TCC ACC TCC ATG TTC TTC CGG GTG GTA GGC ACA CCT TCA

K H S T S M F F R V V G T P S

CCC TCT CTC TGC CCC GCC TGA

P S L C P A *

>EU826594,002H01,splice variant of KIT,KIT-413

ATG AGA GGC GCT CGC GGC GCC TGG GAT TTT CTC TGC GTT CTG CTC

M R G A R G A W D F L C V L L

CTA CTG CTT CGC GTC CAG ACA GGC TCT TCT CAA CCA TCT GTG AGT

L L L R V Q T G S S Q P S V S

CCA GGG GAA CCG TCT CCA CCA TCC ATC CAT CCA GGA AAA TCA GAC

P G E P S P P S I H P G K S D

TTA ATA GTC CGC GTG GGC GAC GAG ATT AGG CTG TTA TGC ACT GAT

L I V R V G D E I R L L C T D

CCG GGC TTT GTC AAA TGG ACT TTT GAG ATC CTG GAT GAA ACG AAT

P G F V K W T F E I L D E T N

GAG AAT AAG CAG AAT GAA TGG ATC ACG GAA AAG GCA GAA GCC ACC

E N K Q N E W I T E K A E A T

AAC ACC GGC AAA TAC ACG TGC ACC AAC AAA CAC GGC TTA AGC AAT

N T G K Y T C T N K H G L S N

TCC ATT TAT GTG TTT GTT AGA GAT CCT GCC AAG CTT TTC CTT GTT

S I Y V F V R D P A K L F L V

GAC CGC TCC TTG TAT GGG AAA GAA GAC AAC GAC ACG CTG GTC CGC

D R S L Y G K E D N D T L V R

TGT CCT CTC ACA GAC CCA GAA GTG ACC AAT TAT TCC CTC AAG GGG

C P L T D P E V T N Y S L K G

TGC CAG GGG AAG CCT CTT CCC AAG GAC TTG AGG TTT ATT CCT GAC

C Q G K P L P K D L R F I P D

CCC AAG GCG GGC ATC ATG ATC AAA AGT GTG AAA CGC GCC TAC CAT

P K A G I M I K S V K R A Y H

CGG CTC TGT CTG CAT TGT TCT GTG GAC CAG GAG GGC AAG TCA GTG

R L C L H C S V D Q E G K S V

CTG TCG GAA AAA TTC ATC CTG AAA GTG AGG CCA GCC TTC AAA GCT

L S E K F I L K V R P A F K A

GTG CCT GTT GTG TCT GTG TCC AAA GCA AGC TAT CTT CTT AGG GAA

V P V V S V S K A S Y L L R E

GGG GAA GAA TTC ACA GTG ACG TGC ACA ATA AAA GAT GTG TCT AGT

G E E F T V T C T I K D V S S

TCT GTG TAC TCA ACG TGG AAA AGA GAA AAC AGT CAG ACT AAA CTA

S V Y S T W K R E N S Q T K L

CAG GAG AAA TAT AAT AGC TGG CAT CAC GGT GAC TTC AAT TAT GAA

Q E K Y N S W H H G D F N Y E

CGT CAG GCA ACG TTG ACT ATC AGT TCA GCG AGA GTT AAT GAT TCT

R Q A T L T I S S A R V N D S

GGA GTG TTC ATG TGT TAT GCC AAT AAT ACT TTT GGA TCA GCA AAT

G V F M C Y A N N T F G S A N

GTC ACA ACA ACC TTG GAA GTA GTA GAT AAA GGA TTC ATT AAT ATC

V T T T L E V V D K G F I N I

TTC CCC ATG ATA AAC ACT ACA GTA TTT GTA AAC GAT GGA GAA AAT

F P M I N T T V F V N D G E N

GTA GAT TTG ATT GTT GAA TAT GAA GCA TTC CCC AAA CCT GAA CAC

V D L I V E Y E A F P K P E H

CAG CAG TGG ATC TAT ATG AAC AGA ACC TTC ACT GAT AAA TGG GAA

Q Q W I Y M N R T F T D K W E

GAT TAT CCC AAG TCT GAG AAT GAA AGT AAT ATC AGA TAC GTA AGT

D Y P K S E N E S N I R Y V S

GAA CTT CAT CTA ACG AGA TTA AAA GGC ACC GAA GGA GGC ACT TAC

E L H L T R L K G T E G G T Y

ACA TTC CTA GTG TCC AAT TCT GAC GTC AAT GCT GCC ATA GCA TTT

T F L V S N S D V N A A I A F

AAT GTT TAT GTG AAT ACT TCC CTG TAA

N V Y V N T S L *

>EU826595,007C09,splice variant of PDGFRB,PDGFRB-336

ATG CGG CTT CCG GGT GCG ATG CCA GCT CTG GCC CTC AAA GGC GAG

M R L P G A M P A L A L K G E

CTG CTG TTG CTG TCT CTC CTG TTA CTT CTG GAA CCA CAG ATC TCT

L L L L S L L L L L E P Q I S

CAG GGC CTG GTC GTC ACA CCC CCG GGG CCA GAG CTT GTC CTC AAT

Q G L V V T P P G P E L V L N

GTC TCC AGC ACC TTC GTT CTG ACC TGC TCG GGT TCA GCT CCG GTG

V S S T F V L T C S G S A P V

GTG TGG GAA CGG ATG TCC CAG GAG CCC CCA CAG GAA ATG GCC AAG

V W E R M S Q E P P Q E M A K

GCC CAG GAT GGC ACC TTC TCC AGC GTG CTC ACA CTG ACC AAC CTC

A Q D G T F S S V L T L T N L

ACT GGG CTA GAC ACG GGA GAA TAC TTT TGC ACC CAC AAT GAC TCC

T G L D T G E Y F C T H N D S

CGT GGA CTG GAG ACC GAT GAG CGG AAA CGG CTC TAC ATC TTT GTG

R G L E T D E R K R L Y I F V

CCA GAT CCC ACC GTG GGC TTC CTC CCT AAT GAT GCC GAG GAA CTA

P D P T V G F L P N D A E E L

TTC ATC TTT CTC ACG GAA ATA ACT GAG ATC ACC ATT CCA TGC CGA

F I F L T E I T E I T I P C R

GTA ACA GAC CCA CAG CTG GTG GTG ACA CTG CAC GAG AAG AAA GGG

V T D P Q L V V T L H E K K G

GAC GTT GCA CTG CCT GTC CCC TAT GAT CAC CAA CGT GGC TTT TCT

D V A L P V P Y D H Q R G F S

GGT ATC TTT GAG GAC AGA AGC TAC ATC TGC AAA ACC ACC ATT GGG

G I F E D R S Y I C K T T I G

GAC AGG GAG GTG GAT TCT GAT GCC TAC TAT GTC TAC AGA CTC CAG

D R E V D S D A Y Y V Y R L Q

GTG TCA TCC ATC AAC GTC TCT GTG AAC GCA GTG CAG ACT GTG GTC

V S S I N V S V N A V Q T V V

CGC CAG GGT GAG AAC ATC ACC CTC ATG TGC ATT GTG ATC GGG AAT

R Q G E N I T L M C I V I G N

GAG GTG GTC AAC TTC GAG TGG ACA TAC CCC CGC AAA GAA AGT GGG

E V V N F E W T Y P R K E S G

CGG CTG GTG GAG CCG GTG ACT GAC TTC CTC TTG GAT ATG CCT TAC

R L V E P V T D F L L D M P Y

CAC ATC CGC TCC ATC CTG CAC ATC CCC AGT GCC GAG TTA GAA GAC

H I R S I L H I P S A E L E D

TCG GGG ACC TAC ACC TGC AAT GTG ACG GAG AGT GTG AAT GAC CAT

S G T Y T C N V T E S V N D H

CAG GAT GAA AAG GCC ATC AAC ATC ACC GTG AGA GCG GCT ACG TGC

Q D E K A I N I T V R A A T C

GGC TCC TGG GAG AGG TGG GCA CAC TAC AAT TTG CTG AGC TGC ATC

G S W E R W A H Y N L L S C I

GGA GCC GGA CAC TGC AGG TAG

G A G H C R *

>EU826596,001E12,splice variant of FGFR1,FGFR1-228

ATG TGG AGC TGG AAG TGC CTC CTC TTC TGG GCT GTG CTG GTC ACA

M W S W K C L L F W A V L V T

GCC ACA CTC TGC ACC GCT AGG CCG TCC CCG ACC TTG CCT GAA CAA

A T L C T A R P S P T L P E Q

GAT GCT CTC CCC TCC TCG GAG GAT GAT GAT GAT GAT GAT GAC TCC

D A L P S S E D D D D D D D S

TCT TCA GAG GAG AAA GAA ACA GAT AAC ACC AAA CCA AAC CCC GTA

S S E E K E T D N T K P N P V

GCT CCA TAT TGG ACA TCC CCA GAA AAG ATG GAA AAG AAA TTG CAT

A P Y W T S P E K M E K K L H

GCA GTG CCG GCT GCC AAG ACA GTG AAG TTC AAA TGC CCT TCC AGT

A V P A A K T V K F K C P S S

GGG ACC CCA AAC CCC ACA CTG CGC TGG TTG AAA AAT GGC AAA GAA

G T P N P T L R W L K N G K E

TTC AAA CCT GAC CAC AGA ATT GGA GGC TAC AAG GTC CGT TAT GCC

F K P D H R I G G Y K V R Y A

ACC TGG AGC ATC ATA ATG GAC TCT GTG GTG CCC TCT GAC AAG GGC

T W S I I M D S V V P S D K G

AAC TAC ACC TGC ATT GTG GAG AAT GAG TAC GGC AGC ATC AAC CAC

N Y T C I V E N E Y G S I N H

ACA TAC CAG CTG GAT GTC GTG GAG CGG TCC CCT CAC CGG CCC ATC

T Y Q L D V V E R S P H R P I

CTG CAA GCA GGG TTG CCC GCC AAC AAA ACA GTG GCC CTG GGT AGC

L Q A G L P A N K T V A L G S

AAC GTG GAG TTC ATG TGT AAG GTG TAC AGT GAC CCG CAG CCG CAC

N V E F M C K V Y S D P Q P H

ATC CAG TGG CTA AAG CAC ATC GAG GTG AAT GGG AGC AAG ATT GGC

I Q W L K H I E V N G S K I G

CCA GAC AAC CTG CCT TAT GTC CAG ATC TTG AAG CCC TGG AAG AGA

P D N L P Y V Q I L K P W K R

GGC CGG CAG TGA

G R Q *

>EU826597,022C02,splice variant of FGFR1,FGFR1-320

ATG TGG AGC TGG AAG TGC CTC CTC TTC TGG GCT GTG CTG GTC ACA

M W S W K C L L F W A V L V T

GCC ACA CTC TGC ACC GCT AGG CCG TCC CCG ACC TTG CCT GAA CAA

A T L C T A R P S P T L P E Q

GAT GCT CTC CCC TCC TCG GAG GAT GAT GAT GAT GAT GAT GAC TCC

D A L P S S E D D D D D D D S

TCT TCA GAG GAG AAA GAA ACA GAT AAC ACC AAA CCA AAC CGT ATG

S S E E K E T D N T K P N R M

CCC GTA GCT CCA TAT TGG ACA TCC CCA GAA AAG ATG GAA AAG AAA

P V A P Y W T S P E K M E K K

TTG CAT GCA GTG CCG GCT GCC AAG ACA GTG AAG TTC AAA TGC CCT

L H A V P A A K T V K F K C P

TCC AGT GGG ACC CCA AAC CCC ACA CTG CGC TGG TTG GAA AAT GGC

S S G T P N P T L R W L E N G

AAA GAA TTC AAA CCT GGC CAC AGA ATT GGA GGC TAC AAG GTC CGT

K E F K P G H R I G G Y K V R

TAT GCC ACC TGG AGC ATC ATA ATG GAC TCT GTG GTG CCC TCT GAC

Y A T W S I I M D S V V P S D

AAG GGC AAC TAC ACC TGC ATT GTG GAG AAT GAG TAC GGC AGC ATC

K G N Y T C I V E N E Y G S I

AAC CAC ACA TAC CAG CTG GAT GTC GTG GAG CGG TCC CCT CAC CGG

N H T Y Q L D V V E R S P H R

CCC ATC CTG CAA GCA GGG TTG CCC GCC AAC AAA ACA GTG GCC CTG

P I L Q A G L P A N K T V A L

GGT AGC AAC GTG GAG TTC ATG TGT AAG GTG TAC AGT GAC CCG CAG

G S N V E F M C K V Y S D P Q

CCG CAC ATC CAG TGG CTA AAG CAC ATC GAG GTG AAT GGG AGC AAG

P H I Q W L K H I E V N G S K

ATT GGC CCG GAC AAC CTG CCT TAT GTC CAG ATC TTG AAG ACT GCT

I G P D N L P Y V Q I L K T A

GGA GTT AAT ACC ACC GAC AAA GAG ATG GAG GTG CTT CAC TTA AGA

G V N T T D K E M E V L H L R

AAT GTC TCC TTT GAG GAC GCA GGG GAG TAT ACG TGC TTG GCG GGT

N V S F E D A G E Y T C L A G

AAC TCT ATC GGA CTC TCC CAT CAC TCT GCA TGG TTG ACC GTT CTG

N S I G L S H H S A W L T V L

GAA GGT ACA CAC TGT AAC TTC TCC TCT CGA TGT CCT GCC CTC GCC

E G T H C N F S S R C P A L A

ACG GGC ACG GGG GGA GCA TGC ATT TCC AGG CTT GGG GAG ACA CAG

T G T G G A C I S R L G E T Q

AGG CAG GAG AGC TGG AAG AAT GGG CTC CTG CCT GCC TGG TGC CAC

R Q E S W K N G L L P A W C H

ATC CTG CCC CAG CTT TGA

I L P Q L *

>EU826598,022D06,splice variant of FGFR2,FGFR2-396

ATG GTC AGC TGG GGT CGT TTC ATC TGC CTG GTC GTG GTC ACC ATG

M V S W G R F I C L V V V T M

GCA ACC TTG TCC CTG GCC CGG CCC TCC TTC AGT TTA GTT GAG GAT

A T L S L A R P S F S L V E D

ACC ACA TTA GAG CCA GAA GAG CCA CCA ACC AAA TAC CAA ATC TCT

T T L E P E E P P T K Y Q I S

CAA CCA GAA GTG TAC GTG GCT GCG CCA GGG GAG TCG CTA GAG GTG

Q P E V Y V A A P G E S L E V

CGC TGC CTG TTG AAA GAT GCC GCC GTG ATC AGT TGG ACT AAG GAT

R C L L K D A A V I S W T K D

GGG GTG CAC TTG GGG CCC AAC AAT AGG ACA GTG CTT ATT GGG GAG

G V H L G P N N R T V L I G E

TAC TTG CAG ATA AAG GGC GCC ACG CCT AGA GAC TCC GGC CTC TAT

Y L Q I K G A T P R D S G L Y

GCT TGT ACT GCC AGT AGG ACT GTA GAC AGT GAA ACT TGG TAC TTC

A C T A S R T V D S E T W Y F

ATG GTG AAT GTC ACA GAT GCC ATC TCA TCC GGA GAT GAT GAG GAT

M V N V T D A I S S G D D E D

GAC ACC GAT GGT GCG GAA GAT TTT GTC AGT GAG AAC AGT AAC AAC

D T D G A E D F V S E N S N N

AAG AGA GCA CCA TAC TGG ACC AAC ACA GAA AAG ACG GAA AAG CGG

K R A P Y W T N T E K T E K R

CTC CAT GCT GTG CCT GCG GCC AAC ACT GTC AAG TTT CGC TGC CCA

L H A V P A A N T V K F R C P

GCC GGG GGG AAC CCA ATG CCA ACC ATG CGG TGG CTG AAA AAC GGG

A G G N P M P T M R W L K N G

AAG GAG TTT AAG CAG GAG CAT CGC ATT GGA GGC TAC AAG GTA CGA

K E F K Q E H R I G G Y K V R

AAC CAG CAC TGG AGC CTC ATT ATG GAA AGT GTG GTC CCA TCT GAC

N Q H W S L I M E S V V P S D

AAG GGA AAT TAT ACC TGT GTG GTG GAG AAT GAA TAC GGG TCC ATC

K G N Y T C V V E N E Y G S I

AAT CAC ACG TAC CAC CTG GAT GTT GTG GAG CGA TCG CCT CAC CGG

N H T Y H L D V V E R S P H R

CCC ATC CTC CAA GCC GGA CTG CCG GCA AAT GCC TCC ACA GTG GTC

P I L Q A G L P A N A S T V V

GGA GGA GAC GTA GAG TTT GTC TGC AAG GTT TAC AGT GAT GCC CAG

G G D V E F V C K V Y S D A Q

CCC CAC ATC CAG TGG ATC AAG CAC GTG GAA AAG AAC GGC AGT AAA

P H I Q W I K H V E K N G S K

TAC GGG CCC GAC GGG CTG CCC TAC CTC AAG GTT CTC AAG GCC GCC

Y G P D G L P Y L K V L K A A

GGT GTT AAC ACC ACG GAC AAA GAG ATT GAG GTT CTC TAT ATT CGG

G V N T T D K E I E V L Y I R

AAT GTA ACT TTT GAG GAC GCT GGG GAA TAT ACG TGC TTG GCG GGT

N V T F E D A G E Y T C L A G

AAT TCT ATT GGG ATA TCC TTT CAC TCT GCA TGG TTG ACA GTT CTG

N S I G I S F H S A W L T V L

CCA GGT ATA TAC TGT TCT TTC TCT CTG GGT TTT TTT CCC TTT TCT

P G I Y C S F S L G F F P F S

TGG TTG ACT GCT ATA AAA TTA ACA CAG CTT CTG TTA TCA GAA ATG

W L T A I K L T Q L L L S E M

GCC CCT TTT ATC CTT GCA TAA

A P F I L A *

>EU826599,022C11,splice variant of FGFR2,FGFR2-317

ATG GTC AGC TGG GGT CGT TTC ATC TGC CTG GTC GTG GTC ACC ATG

M V S W G R F I C L V V V T M

GCA ACC TTG TCC CTG GCC CGG CCC TCC TTC AGT TTA GTT GAG GAT

A T L S L A R P S F S L V E D

ACC ACA TTA GAG CCA GAA GAG CCA CCA ACC AAA TAC CAA ATC TCT

T T L E P E E P P T K Y Q I S

CAA CCA GAA GTG TAC GTG GCT GCG CCA GGG GAG TCG CTA GAG GTG

Q P E V Y V A A P G E S L E V

CGC TGC CTG TTG AAA GAT GCC GCC GTG ATC AGT TGG ACT AAG GAT

R C L L K D A A V I S W T K D

GGG GTG CAC TTG GGG CCC AAC AAT AGG ACA GTG CTT ATT GGG GAG

G V H L G P N N R T V L I G E

TAC TTG CAG ATA AAG GGC GCC ACG CCT AGA GAC TCC GGC CTC TAT

Y L Q I K G A T P R D S G L Y

GCT TGT ACT GCC AGT AGG ACT GTA GAC AGT GAA ACT TGG TAC TTC

A C T A S R T V D S E T W Y F

ATG GTG AAT GTC ACA GAT GCC ATC TCA TCC GGA GAT GAT GAG GAT

M V N V T D A I S S G D D E D

GAC ACC GAT GGT GCG GAA GAT TTT GTC AGT GAG AAC AGT AAC AAC

D T D G A E D F V S E N S N N

AAG AGA GCA CCA TAC TGG ACC AAC ACA GAA AAG ATG GAA AAG CGG

K R A P Y W T N T E K M E K R

CTC CAT GCT GTG CCT GCG GCC AAC ACT GTC AAG TTT CGC TGC CCA

L H A V P A A N T V K F R C P

GCC GGG GGG AAC CCA ATG CCA ACC ATG CGG TGG CTG AAA AAC GGG

A G G N P M P T M R W L K N G

AAG GAG TTT AAG CAG GAG CAT CGC ATT GGA GGC TAC AAG GTA CGA

K E F K Q E H R I G G Y K V R

AAC CAG CAC TGG AGC CTC ATT ATG GAA AGT GTG GTC CCA TCT GAC

N Q H W S L I M E S V V P S D

AAG GGA AAT TAT ACC TGT GTG GTG GAG AAT GAA TAC GGG TCC ATC

K G N Y T C V V E N E Y G S I

AAT CAC ACG TAC CAC CTG GAT GTT GTG GAG CGA TCG CCT CAC CGG

N H T Y H L D V V E R S P H R

CCC ATC CTC CAA GCC GGA CTG CCG GCA AAT GCC TCC ACA GTG GTC

P I L Q A G L P A N A S T V V

GGA GGA GAC GTA GAG TTT GTC TGC AAG GTT TAC AGT GAT GCC CAG

G G D V E F V C K V Y S D A Q

CCC CAC ATC CAG TGG ATC AAG CAC GTG GAA AAG AAC GGC AGT AAA

P H I Q W I K H V E K N G S K

TAC GGG CCC GAC GGG CTG CCC TAC CTC AAG GTT CTC AAG GTG AGG

Y G P D G L P Y L K V L K V R

ACT TTC TGA

T F *

>EU826600,022C10,splice variant of FGFR2,FGFR2-266

ATG GTC AGC TGG GGT CGT TTC ATC TGC CTG GTC GTG GTC ACC ATG

M V S W G R F I C L V V V T M

GCA ACC TTG TCC CTG GCC CGG CCC TCC TTC AGT TTA GTT GAG GAT

A T L S L A R P S F S L V E D

ACC ACA TTA GAG CCA GAA GAG CCA CCA ACC AAA TAC CAA ATC TCT

T T L E P E E P P T K Y Q I S

CAA CCA GAA GTG TAC GTG GCT GCG CCA GGG GAG TCG CTA GAG GTG

Q P E V Y V A A P G E S L E V

CGC TGC CTG TTG AAA GAT GCC GCC GTG ATC AGT TGG ACT AAG GAT

R C L L K D A A V I S W T K D

GGG GTG CAC TTG GGG CCC AAC AAT AGG ACA GTG CTT ATT GGG GAG

G V H L G P N N R T V L I G E

TAC TTG CAG ATA AAG GGC GCC ACG CCT AGA GAC TCC GGC CTC TAT

Y L Q I K G A T P R D S G L Y

GCT TGT ACT GCC AGT AGG ACT GTA GAC AGT GAA ACT TGG TAC TTC

A C T A S R T V D S E T W Y F

ATG GTG AAT GTC ACA GAT GCC ATC TCA TCC GGA GAT GAT GAG GAT

M V N V T D A I S S G D D E D

GAC ACC GAT GGT GCG GAA GAT TTT GTC AGT GAG AAC AGT AAC AAC

D T D G A E D F V S E N S N N

AAG AGA GCA CCA TAC TGG ACC AAC ACA GAA AAG ATG GAA AAG CGG

K R A P Y W T N T E K M E K R

CTC CAT GCT GTG CCT GCG GCC AAC ACT GTC AAG TTT CGC TGC CCA

L H A V P A A N T V K F R C P

GCC GGG GGG AAC CCA ATG CCA ACC ATG CGG TGG CTG AAA AAC GGG

A G G N P M P T M R W L K N G

AAG GAG TTT AAG CAG GAG CAT CGC ATT GGA GGC TAC AAG GTA CGA

K E F K Q E H R I G G Y K V R

AAC CAG CAC TGG AGC CTC ATT ATG GAA AGT GTG GTC CCA TCT GAC

N Q H W S L I M E S V V P S D

AAG GGA AAT TAT ACC TGT GTG GTG GAG AAT GAA TAC GGG TCC ATC

K G N Y T C V V E N E Y G S I

AAT CAC ACG TAC CAC CTG GAT GTT GTG GGT GAG TCT GCC TCT CCT

N H T Y H L D V V G E S A S P

CGT GTG GCG GCT GCA TAC CAG CCC ATT CTT GCT TGA

R V A A A Y Q P I L A *

>EU826601,022D04,splice variant of FGFR2,FGFR2-281

ATG GTC AGC TGG GGT CGT TTC ATC TGC CTG GTC GTG GTC ACC ATG

M V S W G R F I C L V V V T M

GCA ACC TTG TCC CTG GCC CGG CCC TCC TTC AGT TTA GTT GAG GAT

A T L S L A R P S F S L V E D

ATC ACA TTA GAG CCA GAA GGA GCA CCA TAC TGG ACC AAC ACA GAA

I T L E P E G A P Y W T N T E

AAG ATG GAA AAG CGG CTC CAT GCT GTG CCT GCG GCC AAC ACT GTC

K M E K R L H A V P A A N T V

AAG TTT CGC TGC CCA GCC GGG GGG AAC CCA ATG CCA ACC ATG CGG

K F R C P A G G N P M P T M R

TGG CTG AAA AAC GGG AAG GAG TTT AAG CAG GAG CAT CGC ATT GGA

W L K N G K E F K Q E H R I G

GGC TAC AAG GTA CGA AAC CAG CAC TGG AGC CTC ATT ATG GAA AGT

G Y K V R N Q H W S L I M E S

GTG GTC CCA TCT GAC AAG GGA AAT TAT ACC TGT GTG GTG GAG AAT

V V P S D K G N Y T C V V E N

GAA TAC GGG TCC ATC AAT CAC ACG TAC CAC CTG GAT GTT GTG GAG

E Y G S I N H T Y H L D V V E

CGA TCG CCT CAC CGG CCC ATC CTC CAA GCC GGA CTG CCG GCA AAT

R S P H R P I L Q A G L P A N

GCC TCC ACA GTG GTC GGA GGA GAC GTA GAG TTT GTC TGC AAG GTT

A S T V V G G D V E F V C K V

TAC AGT GAT GCC CAG CCC CAC ATC CAG TGG ATC AAG CAC GTG GAA

Y S D A Q P H I Q W I K H V E

AAG AAC GGC AGT AAA TAC GGG CCC GAC GGG CTG CCC TAC CTC AAG

K N G S K Y G P D G L P Y L K

GTT CTC AAG GCC GCC GGT GTT AAC ACC ACG GAC AAA GAG ATT GAG

V L K A A G V N T T D K E I E

GTT CTC TAT ATT CGG AAT GTA ACT TTT GAG GAC GCT GGG GAA TAT

V L Y I R N V T F E D A G E Y

ACG TGC TTG GCG GGT AAT TCT ATT GGG ATA TCC TTT CAC TCC GCA

T C L A G N S I G I S F H S A

TGG TTG ACA GTT CTG CCA GGT ATA TAC TGT TCT TTC TCT CTG GGT

W L T V L P G I Y C S F S L G

TTT TTT CCT TTT TCT TGG TTG ACT GCT ATA AAA TTA ACA CAG CTT

F F P F S W L T A I K L T Q L

CTG TTA TCA GAA ATG GCC CCT TTT ATC CTT GCA TAA

L L S E M A P F I L A *

>EU826602,002A11,splice variant of FGFR4,FGFR4-72

ATG CGG CTG CTG CTG GCC CTG TTG GGG GTC CTG CTG AGT GTG CCT

M R L L L A L L G V L L S V P

GGG CCT CCA GTC TTG TCC CTG GAG GCC TCT GAG GAA GTG GAG CTT

G P P V L S L E A S E E V E L

GCA AGC ATT CAT CTA TCA CTG TGT CTG CGA GAG AGG ACT GGC CTT

A S I H L S L C L R E R T G L

GCA GGG CGC AGG GCC CTA AGC TGG GCT GCA GAG CTG GTG AGC CCT

A G R R A L S W A A E L V S P

GCC TGG CTC CCA GCC TGG AGC AGC AAG AGC AGG AGC TGA

A W L P A W S S K S R S *

>EU826603,002A10,splice variant of FGFR4,FGFR4-446

ATG CGG CTG CTG CTG GCC CTG TTG GGG GTC CTG CTG AGT GTG CCT

M R L L L A L L G V L L S V P

GGG CCT CCA GTC TTG TCC CTG GAG GCC TCT GAG GAA GTG GAG CTT

G P P V L S L E A S E E V E L

GAG CCC TGC CTG GCT CCC AGC CTG GAG CAG CAA GAG CAG GAG CTG

E P C L A P S L E Q Q E Q E L

ACA GTA GCC CTT GGG CAG CCT GTG CGT CTG TGC TGT GGG CGG GCT

T V A L G Q P V R L C C G R A

GAG CGT GGT GGC CAC TGG TAC AAG GAG GGC AGT CGC CTG GCA CCT

E R G G H W Y K E G S R L A P

GCT GGC CGT GTA CGG GGC TGG AGG GGC CGC CTA GAG ATT GCC AGC

A G R V R G W R G R L E I A S

TTC CTA CCT GAG GAT GCT GGC CGC TAC CTC TGC CTG GCA CGA GGC

F L P E D A G R Y L C L A R G

TCC ATG ATC GTC CTG CAG AAT CTC ACC TTG ATT ACA GGT GAC TCC

S M I V L Q N L T L I T G D S

TTG ACC TCC AGC AAC GAT GAT GAG GAC CCC AAG TCC CAT AGG GAC

L T S S N D D E D P K S H R D

CCC TCG AAT AGG CAC AGT TAC CCC CAG CAA GCA CCC TAC TGG ACA

P S N R H S Y P Q Q A P Y W T

CAC CCC CAG CGC ATG GAG AAG AAA CTG CAT GCA GTA CCT GCG GGG

H P Q R M E K K L H A V P A G

AAC ACC GTC AAG TTC CGC TGT CCA GCT GCA GGC AAC CCC ACG CCC

N T V K F R C P A A G N P T P

ACC ATC CGC TGG CTT AAG GAT GGA CAG GCC TTT CAT GGG GAG AAC

T I R W L K D G Q A F H G E N

CGC ATT GGA GGC ATT CGG CTG CGC CAT CAG CAC TGG AGT CTC GTG

R I G G I R L R H Q H W S L V

ATG GAG AGC GTG GTG CCC TCG GAC CGC GGC ACA TAC ACC TGC CTG

M E S V V P S D R G T Y T C L

GTA GAG AAC GCT GTG GGC AGC ATC CGC TAT AAC TAC CTG CTA GAT

V E N A V G S I R Y N Y L L D

GTG CTG GAG CGG TCC CCG CAC CGG CCC ATC CTG CAG GCC GGG CTC

V L E R S P H R P I L Q A G L

CCG GCC AAC ACC ACA GCC GTG GTG GGC AGC GAC GTG GAG CTG CTG

P A N T T A V V G S D V E L L

TGC AAG GTG TAC AGC GAT GCC CAG CCC CAC ATC CAG TGG CTG AAG

C K V Y S D A Q P H I Q W L K

CAC ATC GTC ATC AAC GGC AGC AGC TTC GGA GCC GAC GGT TTC CCC

H I V I N G S S F G A D G F P

TAT GTG CAA GTC CTA AAG ACT GCA GAC ATC AAT AGC TCA GAG GTG

Y V Q V L K T A D I N S S E V

GAG GTC CTG TAC CTG CGG AAC GTG TCA GCC GAG GAC GCA GGC GAG

E V L Y L R N V S A E D A G E

TAC ACC TGC CTC GCA GGC AAT TCC ATC GGC CTC TCC TAC CAG TCT

Y T C L A G N S I G L S Y Q S

GCC TGG CTC ACG GTG CTG CCA GGT GAG CAC CTG AAG GGC CAG GAG

A W L T V L P G E H L K G Q E

ATG CTG CGA GAT GCC CCT CTG GGC CAG CAG TGG GGG CTG TGG CCT

M L R D A P L G Q Q W G L W P

GTT GGG TGG TCA GTC TCT GTT GGC CTG TGG GGT CTG GCC TGG GGG

V G W S V S V G L W G L A W G

GCA GTG TGT GGA TTT GTG GGT TTG AGC TGT ATG ACA GCC CCT CTG

A V C G F V G L S C M T A P L

TGC CTC TCC ACA CGT GGC CGT CCA TGT GAC CGT CTG CTG AGG TGT

C L S T R G R P C D R L L R C

GGG TGC CTG GGA CTG GGC ATA ACT ACA GCT TCC TCC GTG TGT GTC

G C L G L G I T T A S S V C V

CCC ACA TAT GTT GGG AGC TGG GAG GGA CTG AGT TAG

P T Y V G S W E G L S *

>EU826604,004G03,splice variant of EPHA1,EPHA1-474

ATG GAG CGG CGC TGG CCC CTG GGG CTA GGG CTG GTG CTG CTG CTC

M E R R W P L G L G L V L L L

TGC GCC CCG CTG CCC CCG GGG GCG CGC GCC AAG GAA GTT ACT CTG

C A P L P P G A R A K E V T L

ATG GAC ACA AGC AAG GCA CAG GGA GAG CTG GGC TGG CTG CTG GAT

M D T S K A Q G E L G W L L D

CCC CCA AAA GAT GGG TGG AGT GAA CAG CAA CAG ATA CTG AAT GGG

P P K D G W S E Q Q Q I L N G

ACA CCC CTG TAC ATG TAC CAG GAC TGC CCA ATG CAA GGA CGC AGA

T P L Y M Y Q D C P M Q G R R

GAC ACT GAC CAC TGG CTT CGC TCC AAT TGG ATC TAC CGC GGG GAG

D T D H W L R S N W I Y R G E

GAG GCT TCC CGC GTC CAC GTG GAG CTG CAG TTC ACC GTG CGG GAC

E A S R V H V E L Q F T V R D

TGC AAG AGT TTC CCT GGG GGA GCC GGG CCT CTG GGC TGC AAG GAG

C K S F P G G A G P L G C K E

ACC TTC AAC CTT CTG TAC ATG GAG AGT GAC CAG GAT GTG GGC ATT

T F N L L Y M E S D Q D V G I

CAG CTC CGA CGG CCC TTG TTC CAG AAG GTA ACC ACG GTG GCT GCA

Q L R R P L F Q K V T T V A A

GAC CAG AGC TTC ACC ATT CGA GAC CTT GTG TCT GGC TCC GTG AAG

D Q S F T I R D L V S G S V K

CTG AAT GTG GAG CGC TGC TCT CTG GGC CGC CTG ACC CGC CGT GGC

L N V E R C S L G R L T R R G

CTC TAC CTC GCT TTC CAC AAC CCG GGT GCC TGT GTG GCC CTG GTG

L Y L A F H N P G A C V A L V

TCT GTC CGG GTC TTC TAC CAG CGC TGT CCT GAG ACC CTG AAT GGC

S V R V F Y Q R C P E T L N G

TTG GCC CAA TTC CCA GAC ACT CTG CCT GGC CCC GCT GGG TTG GTG

L A Q F P D T L P G P A G L V

GAA GTG GCG GGG ACC TGC TTG CCC CAC GCG CGG GCC AGC CCC AGG

E V A G T C L P H A R A S P R

CCC TCA GGT GCA CCC CGC ATG CAC TGC AGC CCT GAT GGC GAG TGG

P S G A P R M H C S P D G E W

CTG GTG CCT GTA GGA CGG TGC CAC TGT GAG CCT GGC TAT GAG GAA

L V P V G R C H C E P G Y E E

GGT GGC AGT GGC GAA GCA TGT GTT GCC TGC CCT AGC GGC TCC TAC

G G S G E A C V A C P S G S Y

CGG ATG GAC ATG GAC ACA CCC CAT TGT CTC ACG TGC CCC CAG CAG

R M D M D T P H C L T C P Q Q

AGC ACT GCT GAG TCT GAG GGG GCC ACC ATC TGT ACC TGT GAG AGC

S T A E S E G A T I C T C E S

GGC CAT TAC AGA GCT CCC GGG GAG GGC CCC CAG GTG GCA TGC ACA

G H Y R A P G E G P Q V A C T

GGT CCC CCC TCG GCC CCC CGA AAC CTG AGC TTC TCT GCC TCA GGG

G P P S A P R N L S F S A S G

ACT CAG CTC TCC CTG CGT TGG GAA CCC CCA GCA GAT ACG GGG GGA

T Q L S L R W E P P A D T G G

CGC CAG GAT GTC AGA TAC AGT GTG AGG TGT TCC CAG TGT CAG GGC

R Q D V R Y S V R C S Q C Q G

ACA GCA CAG GAC GGG GGG CCC TGC CAG CCC TGT GGG GTG GGC GTG

T A Q D G G P C Q P C G V G V

CAC TTC TCG CCG GGG GCC CGG GGG CTC ACC ACA CCT GCA GTG CAT

H F S P G A R G L T T P A V H

GTC AAT GGC CTT GAA CCT TAT GCC AAC TAC ACC TTT AAT GTG GAA

V N G L E P Y A N Y T F N V E

GCC CAA AAT GGA GTG TCA GGG CTG GGC AGC TCT GGC CAT GCC AGC

A Q N G V S G L G S S G H A S

ACC TCA GTC AGC ATC AGC ATG GGG CAT GCA GGT GAG AGG CTG AGA

T S V S I S M G H A G E R L R

GGG GCT GGG ACA GGG ACC TGG TGG AGA CAG AAG GGC TTA AGA CCA

G A G T G T W W R Q K G L R P

CAG AAC AAA CTG ATG GGC AGG AAG CCA TAG

Q N K L M G R K P *

>EU826605,004H03,splice variant of EPHA1,EPHA1-490

ATG GAG CGG CGC TGG CCC CTG GGG CTA GGG CTG GTG CTG CTG CTC

M E R R W P L G L G L V L L L

TGC GCC CCG CTG CCC CCG GGG GCG CGC GCC AAG GAA GTT ACT CTG

C A P L P P G A R A K E V T L

ATG GAC ACA AGC AAG GCA CAG GGA GAG CTG GGC TGG CTG CTG GAT

M D T S K A Q G E L G W L L D

CCC CCA AAA GAT GGG TGG AGT GAA CAG CAA CAG ATA CTG AAT GGG

P P K D G W S E Q Q Q I L N G

ACA CCC CTG TAC ATG TAC CAG GAC TGC CCA ATG CAA GGA CGC AGA

T P L Y M Y Q D C P M Q G R R

GAC ACT GAC CAC TGG CTT CGC TCC AAT TGG ATC TAC CGC GGG GAG

D T D H W L R S N W I Y R G E

GAG GCT TCC CGC GTC CAC GTG GAG CTG CAG TTC ACC GTG CGG GAC

E A S R V H V E L Q F T V R D

TGC AAG AGT TTC CCT GGG GGA GCC GGG CCT CTG GGC TGC AAG GAG

C K S F P G G A G P L G C K E

ACC TTC AAC CTT CTG TAC ATG GAG AGT GAC CAG GAT GTG GGC ATT

T F N L L Y M E S D Q D V G I

CAG CTC CGA CGG CCC TTG TTC CAG AAG GTA ACC ACG GTG GCT GCA

Q L R R P L F Q K V T T V A A

GAC CAG AGC TTC ACC ATT CGA GAC CTT GTG TCT GGC TCC GTG AAG

D Q S F T I R D L V S G S V K

CTG AAT GTG GAG CGC TGC TCT CTG GGC CGC CTG ACC CGC CGT GGC

L N V E R C S L G R L T R R G

CTC TAC CTC GCT TTC CAC AAC CCG GGT GCC TGT GTG GCC CTG GTG

L Y L A F H N P G A C V A L V

TCT GTC CGG GTC TTC TAC CAG CGC TGT CCT GAG ACC CTG AAT GGC

S V R V F Y Q R C P E T L N G

TTG GCC CAA TTC CCA GAC ACT CTG CCT GGC CCC GCT GGG TTG GTG

L A Q F P D T L P G P A G L V

GAA GTG GCG GGG ACC TGC TTG CCC CAC GCG CGG GCC AGC CCC AGG

E V A G T C L P H A R A S P R

CCC TCA GGT GCA CCC CGC ATG CAC TGC AGC CCT GAT GGC GAG TGG

P S G A P R M H C S P D G E W

CTG GTG CCT GTA GGA CGG TGC CAC TGT GAG CCT GGC TAT GAG GAA

L V P V G R C H C E P G Y E E

GGT GGC AGT GGC GAA GCA TGT GTT GCC TGC CCT AGC GGC TCC TAC

G G S G E A C V A C P S G S Y

CGG ATG GAC ATG GAC ACA CCC CAT TGT CTC ACG TGC CCC CAG CAG

R M D M D T P H C L T C P Q Q

AGC ACT GCT GAG TCT GAG GGG GCC ACC ATC TGT ACC TGT GAG AGC

S T A E S E G A T I C T C E S

GGC CAT TAC AGA GCT CCC GGG GAG GGC CCC CAG GTG GCA TGC ACA

G H Y R A P G E G P Q V A C T

GGT CCC CCC TCG GCC CCC CGA AAC CTG AGC TTC TCT GCC TCA GGG

G P P S A P R N L S F S A S G

ACT CAG CTC TCC CTG CGT TGG GAA CCC CCA GCA GAT ACG GGG GGA

T Q L S L R W E P P A D T G G

CGC CAG GAT GTC AGA TAC AGT GTG AGG TGT TCC CAG TGT CAG GGC

R Q D V R Y S V R C S Q C Q G

ACA GCA CAG GAC GGG GGG CCC TGC CAG CCC TGT GGG GTG GGC GTG

T A Q D G G P C Q P C G V G V

CAC TTC TCG CCG GGG GCC CGG GGG CTC ACC ACA CCT GCA GTG CAT

H F S P G A R G L T T P A V H

GTC AAT GGC CTT GAA CCT TAT GCC AAC TAC ACC TTT AAT GTG GAA

V N G L E P Y A N Y T F N V E

GCC CAA AAT GGA GTG TCA GGG CTG GGC AGC TCT GGC CAT GCC AGC

A Q N G V S G L G S S G H A S

ACC TCA GTC AGC ATC AGC ATG GGG CAT GCA GAT CCA ACC TTA TCC

T S V S I S M G H A D P T L S

CTC TGG ACC CCC AGA GTC ACT GTC AGG CCT GTC TCT GAG ACT GGT

L W T P R V T V R P V S E T G

GAA GAA AGA ACC GAG GCA ACT AGA GCT GAC CTG GGC GGG GTC CCG

E E R T E A T R A D L G G V P

GCC CCG AAG CCC TGG GGC GAA CCT GAC CTA TGA

A P K P W G E P D L *

>EU826606,016E12,splice variant of EPHA2,EPHA2-497

ATG GAG CTC CAG GCA GCC CGC GCC TGC TTC GCC CTG CTG TGG GGC

M E L Q A A R A C F A L L W G

TGT GCG CTG GCC GCG GCC GCG GCG GCG CAG GGC AAG GAA GTG GTA

C A L A A A A A A Q G K E V V

CTG CTG GAC TTT GCT GCA GCT GGA GGG GAG CTC GGC TGG CTC ACA

L L D F A A A G G E L G W L T

CAC CCG TAT GGC AAA GGG TGG GAC CTG ATG CAG AAC ATC ATG AAT

H P Y G K G W D L M Q N I M N

GAC ATG CCG ATC TAC ATG TAC TCC GTG TGC AAC GTG ATG TCT GGC

D M P I Y M Y S V C N V M S G

GAC CAG GAC AAC TGG CTC CGC ACC AAC TGG GTG TAC CGA GGA GAG

D Q D N W L R T N W V Y R G E

GCT GAG CGT ATC TTC ATT GAG CTC AAG TTT ACT GTA CGT GAC TGC

A E R I F I E L K F T V R D C

AAC AGC TTC CCT GGT GGC GCC AGC TCC TGC AAG GAG ACT TTC AAC

N S F P G G A S S C K E T F N

CTC TAC TAT GCC GAG TCG GAC CTG GAC TAC GGC ACC AAC TTC CAG

L Y Y A E S D L D Y G T N F Q

AAG CGC CTG TTC ACC AAG ATT GAC ACC ATT GCG CCC GAT GAG ATC

K R L F T K I D T I A P D E I

ACC GTC AGC AGC GAC TTC GAG GCA CGC CAC GTG AAG CTG AAC GTG

T V S S D F E A R H V K L N V

GAG GAG CGC TCC GTG GGG CCG CTC ACC CGC AAA GGC TTC TAC CTG

E E R S V G P L T R K G F Y L

GCC TTC CAG GAT ATC GGT GCC TGT GTG GCG CTG CTC TCC GTC CGT

A F Q D I G A C V A L L S V R

GTC TAC TAC AAG AAG TGC CCC GAG CTG CTG CAG GGC CTG GCC CAC

V Y Y K K C P E L L Q G L A H

TTC CCT GAG ACC ATC GCC GGC TCT GAT GCA CCT TCC CTG GCC ACT

F P E T I A G S D A P S L A T

GTG GCC GGC ACC TGT GTG GAC CAT GCC GTG GTG CCA CCG GGG GGT

V A G T C V D H A V V P P G G

GAA GAG CCC CGT ATG CAC TGT GCA GTG GAT GGC GAG TGG CTG GTG

E E P R M H C A V D G E W L V

CCC ATT GGG CAG TGC CTG TGC CAG GCA GGC TAC GAG AAG GTG GAG

P I G Q C L C Q A G Y E K V E

GAT GCC TGC CAG GCC TGC TCG CCT GGA TTT TTT AAG TTT GAG GCA

D A C Q A C S P G F F K F E A

TCT GAG AGC CCC TGC TTG GAG TGC CCT GAG CAC ACG CTG CCA TCC

S E S P C L E C P E H T L P S

CCT GAG GGT GCC ACC TCC TGC GAG TGT GAG GAA GGC TTC TTC CGG

P E G A T S C E C E E G F F R

GCA CCT CAG GAC CCA GCG TCG ATG CCT TGC ACA CGA CCC CCC TCC

A P Q D P A S M P C T R P P S

GCC CCA CAC TAC CTC ACA GCC GTG GGC ATG GGT GCC AAG GTG GAG

A P H Y L T A V G M G A K V E

CTG CGC TGG ACG CCC CCT CAG GAC AGC GGG GGC CGC GAG GAC ATT

L R W T P P Q D S G G R E D I

GTC TAC AGC GTC ACC TGC GAA CAG TGC TGG CCC GAG TCT GGG GAA

V Y S V T C E Q C W P E S G E

TGC GGG CCG TGT GAG GCC AGT GTG CGC TAC TCG GAG CCT CCT CAC

C G P C E A S V R Y S E P P H

GGA CTG ACC CGC ACC AGT GTG ACA GTG AGC GAC CTG GAG CCC CAC

G L T R T S V T V S D L E P H

ATG AAC TAC ACC TTC ACC GTG GAG GCC CGC AAT GGC GTC TCA GGC

M N Y T F T V E A R N G V S G

CTG GTA ACC AGC CGC AGC TTC CGT ACT GCC AGT GTC AGC ATC AAC

L V T S R S F R T A S V S I N

CAG ACA GAG CCC CCC AAG GTG AGG CTG GAG GGC CGC AGC ACC ACC

Q T E P P K V R L E G R S T T

TCG CTT AGC GTC TCC TGG AGC ATC CCC CCG CCG CAG CAG AGC CGA

S L S V S W S I P P P Q Q S R

GTG TGG AAG TAC GAG GTC ACT TAC CGC AAG AAG gtA ACT CCC AGA

V W K Y E V T Y R K K V T P R

GGG GCG GGG CTG GCT CTA GCT GGG CCG ACA GCA GGG GAC AGG CTG

G A G L A L A G P T A G D R L

GTC ACA tag

V T *

>EU826607,005D06,splice variant of EPHB1,EPHB1-242

ATG GCC CTG GAT TAT CTA CTA CTG CTC CTC CTG GCA TCC GCA GTG

M A L D Y L L L L L L A S A V

GCT GCG ATG GAA GAA ACG TTA ATG GAC ACC AGA ACG GCT ACT GCA

A A M E E T L M D T R T A T A

GAG CTG GGC TGG ACG GCC AAT CCT GCG TCC GGG TGG GAA GAA GTC

E L G W T A N P A S G W E E V

AGT GGC TAC GAT GAA AAC CTG AAC ACC ATC CGC ACC TAC CAG GTG

S G Y D E N L N T I R T Y Q V

TGC AAT GTC TTC GAG CCC AAC CAG AAC AAT TGG CTG CTC ACC ACC

C N V F E P N Q N N W L L T T

TTC ATC AAC CGG CGG GGG GCC CAT CGC ATC TAC ACA GAG ATG CGC

F I N R R G A H R I Y T E M R

TTC ACT GTG AGA GAC TGC AGC AGC CTC CCT AAT GTC CCA GGA TCC

F T V R D C S S L P N V P G S

TGC AAG GAG ACC TTC AAC TTG TAT TAC TAT GAG ACT GAC TCT GTC

C K E T F N L Y Y Y E T D S V

ATT GCC ACC AAG AAG TCA GCC TTC TGG TCT GAG GCC CCC TAC CTC

I A T K K S A F W S E A P Y L

AAA GTA GAC ACC ATT GCT GCA GAT GAG AGC TTC TCC CAG GTG GAC

K V D T I A A D E S F S Q V D

TTT GGG GGA AGG CTG ATG AAG CTT GCC CTG CAG GGA CAT TCA AGG

F G G R L M K L A L Q G H S R

CCA GCC AGG AAG CTG AAG GCT GCT CCC ACT GCC CCT CCA ACA GCC

P A R K L K A A P T A P P T A

GCT CCC CTG CAG AGG CGT CTC CCA TCT GCA CCT GTC GGA CCG GTT

A P L Q R R L P S A P V G P V

ATT ACC GAG CGG ACT TTG ACC CTC CAG AAG TGG CAT GCA CTA GCG

I T E R T L T L Q K W H A L A

TCC CAT CAG GTC CCC GCA ATG TTA TCT CCA TCG TCA ATG AGA CGT

S H Q V P A M L S P S S M R R

CCA TCA TTC TGG AGT GGC ACC CTC CAA GGG AGA CAG GTG GGC GGG

P S F W S G T L Q G R Q V G G

ATG ATG TGA

M M *

>EU826608,012D11,splice variant of EPHB4,EPHB4-516

ATG GAG CTC CGG GTG CTG CTC TGC TGG GCT TCG TTG GCC GCA GCT

M E L R V L L C W A S L A A A

TTG GAA GAG ACC CTG CTG AAC ACA AAA TTG GAA ACT GCT GAT CTG

L E E T L L N T K L E T A D L

AAG TGG GTG ACA TTC CCT CAG GTG GAC GGG CAG TGG GAG GAA CTG

K W V T F P Q V D G Q W E E L

AGC GGC CTG GAT GAG GAA CAG CAC AGC GTG CGC ACC TAC GAA GTG

S G L D E E Q H S V R T Y E V

TGT GAC GTG CAG CGT GCC CCG GGC CAG GCC CAC TGG CTT CGC ACA

C D V Q R A P G Q A H W L R T

GGT TGG GTC CCA CGG CGG GGC GCC GTC CAC GTG TAC GCC ACG CTG

G W V P R R G A V H V Y A T L

CGC TTC ACC ATG CTC GAG TGC CTG TCC CTG CCT CGG GCT GGG CGC

R F T M L E C L S L P R A G R

TCC TGC AAG GAG ACC TTC ACC GTC TTC TAC TAT GAG AGC GAT GCG

S C K E T F T V F Y Y E S D A

GAC ACG GCC ACG GCC CTC ACG CCA GCC TGG ATG GAG AAC CCC TAC

D T A T A L T P A W M E N P Y

ATC AAG GTG GAC ACG GTG GCC GCG GAG CAT CTC ACC CGG AAG CGC

I K V D T V A A E H L T R K R

CCT GGG GCC GAG GCC ACC GGG AAG GTG AAT GTC AAG ACG CTG CGT

P G A E A T G K V N V K T L R

CTG GGA CCG CTC AGC AAG GCT GGC TTC TAC CTG GCC TTC CAG GAC

L G P L S K A G F Y L A F Q D

CAG GGT GCC TGC ATG GCC CTG CTA TCC CTG CAC CTC TTC TAC AAA

Q G A C M A L L S L H L F Y K

AAG TGC GCC CAG CTG ACT GTG AAC CTG ACT CGA TTC CCG GAG ACT

K C A Q L T V N L T R F P E T

GTG CCT CGG GAG CTG GTT GTG CCC GTG GCC GGT AGC TGC GTG GTG

V P R E L V V P V A G S C V V

GAT GCC GTC CCC GCC CCT GGC CCC AGC CCC AGC CTC TAC TGC CGT

D A V P A P G P S P S L Y C R

GAG GAT GGC CAG TGG GCC GAA CAG CCG GTC ACG GGC TGC AGC TGT

E D G Q W A E Q P V T G C S C

GCT CCG GGG TTC GAG GCA GCT GAG GGG AAC ACC AAG TGC CGA GCC

A P G F E A A E G N T K C R A

TGT GCC CAG GGC ACC TTC AAG CCC CTG TCA GGA GAA GGG TCC TGC

C A Q G T F K P L S G E G S C

CAG CCA TGC CCA GCC AAT AGC CAC TCT AAC ACC ATT GGA TCA GCC

Q P C P A N S H S N T I G S A

GTC TGC CAG TGC CGC GTC GGG TAC TTC CGG GCA CGC ACA GAC CCC

V C Q C R V G Y F R A R T D P

CGG GGT GCA CCC TGC ACC ACC CCT CCT TCG GCT CCG CGG AGC GTG

R G A P C T T P P S A P R S V

GTT TCC CGC CTG AAC GGC TCC TCC CTG CAC CTG GAA TGG AGT GCC

V S R L N G S S L H L E W S A

CCC CTG GAG TCT GGT GGC CGA GAG GAC CTC ACC TAC GCC CTC CGC

P L E S G G R E D L T Y A L R

TGC CGG GAG TGC CGA CCC GGA GGC TCC TGT GCG CCC TGC GGG GGA

C R E C R P G G S C A P C G G

GAC CTG ACT TTT GAC CCC GGC CCC CGG GAC CTG GTG GAG CCC TGG

D L T F D P G P R D L V E P W

GTG GTG GTT CGA GGG CTA CGT CCT GAC TTC ACC TAT ACC TTT GAG

V V V R G L R P D F T Y T F E

GTC ACT GCA TTG AAC GGG GTA TCC TCC TTA GCC ACG GGG CCC GTC

V T A L N G V S S L A T G P V

CCA TTT GAG CCT GTC AAT GTC ACC ACT GAC CGA GAG GTA CCT CCT

P F E P V N V T T D R E V P P

GCA GTG TCT GAC ATC CGG GTG ACG CGG TCC TCA CCC AGC AGC TTG

A V S D I R V T R S S P S S L

AGC CTG GCC TGG GCT GTT CCC CGG GCA CCC AGT GGG GCT GTG CTG

S L A W A V P R A P S G A V L

GAC TAC GAG GTC AAA TAC CAT GAG AAG GGC GCC GAG GGT CCC AGC

D Y E V K Y H E K G A E G P S

AGC GTG CGG TTC CTG AAG ACG TCA GAA AAC CGG GCA GAG CTG CGG

S V R F L K T S E N R A E L R

GGG CTG AAG CGG GGA GCC AGC TAC CTG GTG CAG AGA GCG AGG GCT

G L K R G A S Y L V Q R A R A

GGC GGG AGC AGC TGG CCC TGA

G G S S W P *

>EU826609,012C08,splice variant of EPHB4,EPHB4-306

ATG GAG CTC CGG GTG CTG CTC TGC TGG GCT TCG TTG GCC GCA GCT

M E L R V L L C W A S L A A A

TTG GAA GAG ACC CTG CTG AAC ACA AAA TTG GAA ACT GCT GAT CTG

L E E T L L N T K L E T A D L

AAG TGG GTG ACA TTC CCT CAG GTG GAC GGG CAG TGG GAG GAA CTG

K W V T F P Q V D G Q W E E L

AGC GGC CTG GAT GAG GAA CAG CAC AGC GTG CGC ACC TAC GAA GTG

S G L D E E Q H S V R T Y E V

TGT GAC GTG CAG CGT GCC CCG GGC CAG GCC CAC TGG CTT CGC ACA

C D V Q R A P G Q A H W L R T

GGT TGG GTC CCA CGG CGG GGC GCC GTC CAC GTG TAC GCC ACG CTG

G W V P R R G A V H V Y A T L

CGC TTC ACC ATG CTC GAG TGC CTG TCC CTG CCT CGG GCT GGG CGC

R F T M L E C L S L P R A G R

TCC TGC AAG GAG ACC TTC ACC GTC TTC TAC TAT GAG AGC GAT GCG

S C K E T F T V F Y Y E S D A

GAC ACG GCC ACG GCC CTC ACG CCA GCC TGG ATG GAG AAC CCC TAC

D T A T A L T P A W M E N P Y

ATC AAG GTG GAC ACG GTG GCC GCG GAG CAT CTC ACC CGG AAG CGC

I K V D T V A A E H L T R K R

CCT GGG GCC GAG GCC ACC GGG AAG GTG AAT GTC AAG ACG CTG CGT

P G A E A T G K V N V K T L R

CTG GGA CCG CTC AGC AAG GCT GGC TTC TAC CTG GCC TTC CAG GAC

L G P L S K A G F Y L A F Q D

CAG GGT GCC TGC ATG GCC CTG CTA TCC CTG CAC CTC TTC TAC AAA

Q G A C M A L L S L H L F Y K

AAG TGC GCC CAG CTG ACT GTG AAC CTG ACT CGA TTC CCG GAG ACT

K C A Q L T V N L T R F P E T

GTG CCT CGG GAG CTG GTT GTG CCC GTG GCC GGT AGC TGC GTG GTG

V P R E L V V P V A G S C V V

GAT GCC GTC CCC GCC CCT GGC CCC AGC CCC AGC CTC TAC TGC CGT

D A V P A P G P S P S L Y C R

GAG GAT GGC CAG TGG GCC GAA CAG CCG GTC ACG GGC TGC AGC TGT

E D G Q W A E Q P V T G C S C

GCT CCG GGG TTC GAG GCA GCT GAG GGG AAC ACC AAG TGC CGA GGG

A P G F E A A E G N T K C R G

CGC CGA GGG TCC CAG CAG CGT GCG GTT CCT GAA GAC GTC AGA AAA

R R G S Q Q R A V P E D V R K

CCG GGC AGA GCT GCG GGG GCT GAA GCG GGG AGC CAG CTA CCT GGT

P G R A A G A E A G S Q L P G

GCA GGT ACG GGC GCG CTC TGA

A G T G A L *

>EU826610,012E11,splice variant of EPHB4,EPHB4-414

ATG GAG CTC CGG GTG CTG CTC TGC TGG GCT TCG TTG GCC GCA GCT

M E L R V L L C W A S L A A A

TTG GAA GAG ACC CTG CTG AAC ACA AAA TTG GAA ACT GCT GAT CTG

L E E T L L N T K L E T A D L

AAG TGG GTG ACA TTC CCT CAG GTG GAC GGG CAG TGG GAG GAA CTG

K W V T F P Q V D G Q W E E L

AGC GGC CTG GAT GAG GAA CAG CAC AGC GTG CGC ACC TAC GAA GTG

S G L D E E Q H S V R T Y E V

TGT GAC GTG CAG CGT GCC CCG GGC CAG GCC CAC TGG CTT CGC ACA

C D V Q R A P G Q A H W L R T

GGT TGG GTC CCA CGG CGG GGC GCC GTC CAC GTG TAC GCC ACG CTG

G W V P R R G A V H V Y A T L

CGC TTC ACC ATG CTC GAG TGC CTG TCC CTG CCT CGG GCT GGG CGC

R F T M L E C L S L P R A G R

TCC TGC AAG GAG ACC TTC ACC GTC TTC TAC TAT GAG AGC GAT GCG

S C K E T F T V F Y Y E S D A

GAC ACG GCC ACG GCC CTC ACG CCA GCC TGG ATG GAG AAC CCC TAC

D T A T A L T P A W M E N P Y

ATC AAG GTG GAC ACG GTG GCC GCG GAG CAT CTC ACC CGG AAG CGC

I K V D T V A A E H L T R K R

CCT GGG GCC GAG GCC ACC GGG AAG GTG AAT GTC AAG ACG CTG CGT

P G A E A T G K V N V K T L R

CTG GGA CCG CTC AGC AAG GCT GGC TTC TAC CTG GCC TTC CAG GAC

L G P L S K A G F Y L A F Q D

CAG GGT GCC TGC ATG GCC CTG CTA TCC CTG CAC CTC TTC TAC AAA

Q G A C M A L L S L H L F Y K

AAG TGC GCC CAG CTG ACT GTG AAC CTG ACT CGA TTC CCG GAG ACT

K C A Q L T V N L T R F P E T

GTG CCT CGG GAG CTG GTT GTG CCC GTG GCC GGT AGC TGC GTG GTG

V P R E L V V P V A G S C V V

GAT GCC GTC CCC GCC CCT GGC CCC AGC CCC AGC CTC TAC TGC CGT

D A V P A P G P S P S L Y C R

GAG GAT GGC CAG TGG GCC GAA CAG CCG GTC ACG GGC TGC AGC TGT

E D G Q W A E Q P V T G C S C

GCT CCG GGG TTC GAG GCA GCT GAG GGG AAC ACC AAG TGC CGA GCC

A P G F E A A E G N T K C R A

TGT GCC CAG GGC ACC TTC AAG CCC CTG TCA GGA GAA GGG TCC TGC

C A Q G T F K P L S G E G S C

CAG CCA TGC CCA GCC AAT AGC CAC TCT AAC ACC ATT GGA TCA GCC

Q P C P A N S H S N T I G S A

GTC TGC CAG TGC CGC GTC GGG TAC TTC CGG GCA CGC ACA GAC CCC

V C Q C R V G Y F R A R T D P

CGG GGT GCA CCC TGC ACC ACC CCT CCT TCG GCT CCG CGG AGC GTG

R G A P C T T P P S A P R S V

GTT TCC CGC CTG AAC GGC TCC TCC CTG CAC CTG GAA TGG AGT GCC

V S R L N G S S L H L E W S A

CCC CTG GAG TCT GGT GGC CGA GAG GAC CTC ACC TAC GCC CTC CGC

P L E S G G R E D L T Y A L R

TGC CGG GAG TGC CGA CCC GGA GGC TCC TGT GCG CCC TGC GGG GGA

C R E C R P G G S C A P C G G

GAC CTG ACT TTT GAC CCC GGC CCC CGG GAC CTG GTG GAG CCC TGG

D L T F D P G P R D L V E P W

GTG GTG GTT CGA GGG CTA CGT CCT GAC TTC ACC TAT ACC TTT GAG

V V V R G L R P D F T Y T F E

TAC CTC CTG CAG TGT CTG ACA TCC GGG TGA

Y L L Q C L T S G *

>EU826611,024B04,splice variant of IGF1R,IGF1R-831

atg AAG TCT GGC TCC GGA GGA GGG TCC CCG ACC TCG CTG TGG GGG

M K S G S G G G S P T S L W G

CTC CTG TTT CTC TCC GCC GCG CTC TCG CTC TGG CCG ACG AGT GGA

L L F L S A A L S L W P T S G

GAA ATC TGC GGG CCA GGC ATC GAC ATC CGC AAC GAC TAT CAG CAG

E I C G P G I D I R N D Y Q Q

CTG AAG CGC CTG GAG AAC TGC ACG GTG ATC GAG GGC TAC CTC CAC

L K R L E N C T V I E G Y L H

ATC CTG CTC ATC TCC AAG GCC GAG GAC TAC CGC AGC TAC CGC TTC

I L L I S K A E D Y R S Y R F

CCC AAG CTC ACG GTC ATT ACC GAG TAC TTG CTG CTG TTC CGA GTG

P K L T V I T E Y L L L F R V

GCT GGC CTC GAG AGC CTC GGA GAC CTC TTC CCC AAC CTC ACG GTC

A G L E S L G D L F P N L T V

ATC CGC GGC TGG AAA CTC TTC TAC AAC TAC GCC CTG GTC ATC TTC

I R G W K L F Y N Y A L V I F

GAG GTG ACC AAT CTC AAG GAT ATT GGG CTT TAC AAC CTG AGG AAC

E V T N L K D I G L Y N L R N

ATT ACT CGG GGG GCC ATC AGG ATT GAG AAA AAT GCT GAC CTC TGT

I T R G A I R I E K N A D L C

TAC CTC TCC ACT GTG GAC TGG TCC CTG ATC CTG GAT GCG GTG TCC

Y L S T V D W S L I L D A V S

AAT AAC TAC ATT GTG GGG AAT AAG CCC CCA AAG GAA TGT GGG GAC

N N Y I V G N K P P K E C G D

CTG TGT CCA GGG ACC ATG GAG GAG AAG CCG ATG TGT GAG AAG ACC

L C P G T M E E K P M C E K T

ACC ATC AAC AAT GAG TAC AAC TAC CGC TGC TGG ACC ACA AAC CGC

T I N N E Y N Y R C W T T N R

TGC CAG AAA ATG TGC CCA AGC ACG TGT GGG AAG CGG GCG TGC ACC

C Q K M C P S T C G K R A C T

GAG AAC AAT GAG TGC TGC CAC CCC GAG TGC CTG GGC AGC TGC AGC

E N N E C C H P E C L G S C S

GCG CCT GAC AAC GAC ACG GCC TGT GTA GCT TGC CGC CAC TAC TAC

A P D N D T A C V A C R H Y Y

TAT GCC GGT GTC TGT GTG CCT GCC TGC CCG CCC AAC ACC TAC AGG

Y A G V C V P A C P P N T Y R

TTT GAG GGC TGG CGC TGT GTG GAC CGT GAC TTC TGC GCC AAC ATC

F E G W R C V D R D F C A N I

CTC AGC GCC GAG AGC AGC GAC TCC GAG GGG TTT GTG ATC CAC GAC

L S A E S S D S E G F V I H D

GGC GAG TGC ATG CAG GAG TGC CCC TCG GGC TTC ATC CGC AAC GGC

G E C M Q E C P S G F I R N G

AGC CAG AGC ATG TAC TGC ATC CCT TGT GAA GGT CCT TGC CCG AAG

S Q S M Y C I P C E G P C P K

GTC TGT GAG GAA GAA AAG AAA ACA AAG ACC ATT GAT TCT GTT ACT

V C E E E K K T K T I D S V T

TCT GCT CAG ATG CTC CAA GGA TGC ACC ATC TTC AAG GGC AAT TTG

S A Q M L Q G C T I F K G N L

CTC ATT AAC ATC CGA CGG GGG AAT AAC ATT GCT TCA GAG CTG GAG

L I N I R R G N N I A S E L E

AAC TTC ATG GGG CTC ATC GAG GTG GTG ACG GGC TAC GTG AAG ATC

N F M G L I E V V T G Y V K I

CGC CAT TCT CAT GCC TTG GTC TCC TTG TCC TTC CTA AAA AAC CTT

R H S H A L V S L S F L K N L

CGC CTC ATC CTA GGA GAG GAG CAG CTA GAA GGG AAT TAC TCC TTC

R L I L G E E Q L E G N Y S F

TAC GTC CTC GAC AAC CAG AAC TTG CAG CAA CTG TGG GAC TGG GAC

Y V L D N Q N L Q Q L W D W D

CAC CGC AAC CTG ACC ATC AAA GCA GGG AAA ATG TAC TTT GCT TTC

H R N L T I K A G K M Y F A F

AAT CCC AAA TTA TGT GTT TCC GAA ATT TAC CGC ATG GAG GAA GTG

N P K L C V S E I Y R M E E V

ACG GGG ACT AAA GGG CGC CAA AGC AAA GGG GAC ATA AAC ACC AGG

T G T K G R Q S K G D I N T R

AAC AAC GGG GAG AGA GCC TCC TGT GAA AGT GAC GTC CTG CAT TTC

N N G E R A S C E S D V L H F

ACC TCC ACC ACC ACG TCG AAG AAT CGC ATC ATC ATA ACC TGG CAC

T S T T T S K N R I I I T W H

CGG TAC CGG CCC CCT GAC TAC AGG GAT CTC ATC AGC TTC ACC GTT

R Y R P P D Y R D L I S F T V

TAC TAC AAG GAA GCA CCC TTT AAG AAT GTC ACA GAG TAT GAT GGG

Y Y K E A P F K N V T E Y D G

CAG GAT GCC TGC GGC TCC AAC AGC TGG AAC ATG GTG GAC GTG GAC

Q D A C G S N S W N M V D V D

CTC CCG CCC AAC AAG GAC GTG GAG CCC GGC ATC TTA CTA CAT GGG

L P P N K D V E P G I L L H G

CTG AAG CCC TGG ACT CAG TAC GCC GTT TAC GTC AAG GCT GTG ACC

L K P W T Q Y A V Y V K A V T

CTC ACC ATG GTG GGG AAC GAC CAT ATC CGT GGG GCC AAG AGT GAG

L T M V G N D H I R G A K S E

ATC TTG TAC ATT CGC ACC AAT GCT TCA GTT CCT TCC ATT CCC TTG

I L Y I R T N A S V P S I P L

GAC GTT CTT TCA GCA TCG AAC TCC TCT TCT CAG TTA ATC GTG AAG

D V L S A S N S S S Q L I V K

TGG AAC CCT CCC TCT CTG CCC AAC GGC AAC CTG AGT TAC TAC ATT

W N P P S L P N G N L S Y Y I

GTG CGC TGG CAG CGG CAG CCT CAG GAC GGC TAC CTT TAC CGG CAC

V R W Q R Q P Q D G Y L Y R H

AAT TAC TGC TCC AAA GAC AAA ATC CCC ATC AGG AAG TAT GCC GAC

N Y C S K D K I P I R K Y A D

GGC ACC ATC GAC ATT GAG GAG GTC ACA GAG AAC CCC AAG ACT GAG

G T I D I E E V T E N P K T E

GTG TGT GGT GGG GAG AAA GGG CCT TGC TGC GCC TGC CCC AAA ACT

V C G G E K G P C C A C P K T

GAA GCC GAG AAG CAG GCC GAG AAG GAG GAG GCT GAA TAC CGC AAA

E A E K Q A E K E E A E Y R K

GTC TTT GAG AAT TTC CTG CAC AAC TCC ATC TTC GTG CCC AGA CCT

V F E N F L H N S I F V P R P

GAA AGG AAG CGG AGA GAT GTC ATG CAA GTG GCC AAC ACC ACC ATG

E R K R R D V M Q V A N T T M

TCC AGC CGA AGC AGG AAC ACC ACG GCC GCA GAC ACC TAC AAC ATC

S S R S R N T T A A D T Y N I

ACC GAC CCG GAA GAG CTG GAG ACA GAG TAC CCT TTC TTT GAG AGC

T D P E E L E T E Y P F F E S

AGA GTG GAT AGC AAG GAG AGA ACT GTC ATT TCT AAC CTT CGG CCT

R V D S K E R T V I S N L R P

TTC ACA TTG TAC CGC ATC GAT ATC CAC AGC TGC AAC CAC GAG GCT

F T L Y R I D I H S C N H E A

GAG AAG CTG GGC TGC AGC GCC TCC AAC TTC GTC TTT GCA AGG ACT

E K L G C S A S N F V F A R T

GTG CCC GCA GGT ATG GTA tga

V P A G M V *

>EU826612,024A03,splice variant of IGF1R,IGF1R-759

atg AAG TCT GGC TCC GGA GGA GGG TCC CCG ACC TCG CTG TGG GGG

M K S G S G G G S P T S L W G

CTC CTG TTT CTC TCC GCC GCG CTC TCG CTC TGG CCG ACG AGT GGA

L L F L S A A L S L W P T S G

GAA ATC TGC GGG CCA GGC ATC GAC ATC CGC AAC GAC TAT CAG CAG

E I C G P G I D I R N D Y Q Q

CTG AAG CGC CTG GAG AAC TGC ACG GTG ATC GAG GGC TAC CTC CAC

L K R L E N C T V I E G Y L H

ATC CTG CTC ATC TCC AAG GCC GAG GAC TAC CGC AGC TAC CGC TTC

I L L I S K A E D Y R S Y R F

CCC AAG CTC ACG GTC ATT ACC GAG TAC TTG CTG CTG TTC CGA GTG

P K L T V I T E Y L L L F R V

GCT GGC CTC GAG AGC CTC GGA GAC CTC TTC CCC AAC CTC ACG GTC

A G L E S L G D L F P N L T V

ATC CGC GGC TGG AAA CTC TTC TAC AAC TAC GCC CTG GTC ATC TTC

I R G W K L F Y N Y A L V I F

GAG ATG ACC AAT CTC AAG GAT ATT GGG CTT TAC AAC CTG AGG AAC

E M T N L K D I G L Y N L R N

ATT ACT CGG GGG GCC ATC AGG ATT GAG AAA AAT GCT GAC CTC TGT

I T R G A I R I E K N A D L C

TAC CTC TCC ACT GTG GAC TGG TCC CTG ATC CTG GAT GCG GTG TCC

Y L S T V D W S L I L D A V S

AAT AAC TAC ATT GTG GGG AAT AAG CCC CCA AAG GAA TGT GGG GAC

N N Y I V G N K P P K E C G D

CTG TGT CCA GGG ACC ATG GAG GAG AAG CCG ATG TGT GAG AAG ACC

L C P G T M E E K P M C E K T

ACC ATC AAC AAT GAG TAC AAC TAC CGC TGC TGG ACC ACA AAC CGC

T I N N E Y N Y R C W T T N R

TGC CAG AAA ATG TGC CCA AGC ACG TGT GGG AAG CGG GCG TGC ACC

C Q K M C P S T C G K R A C T

GAG AAC AAT GAG TGC TGC CAC CCC GAG TGC CTG GGC AGC TGC AGC

E N N E C C H P E C L G S C S

GCG CCT GAC AAC GAC ACG GCC TGT GTA GCT TGC CGC CAC TAC TAC

A P D N D T A C V A C R H Y Y

TAT GCC GGT GTC TGT GTG CCT GCC TGC CCG CCT AAC ACC TAC AGG

Y A G V C V P A C P P N T Y R

TTT GAG GGC TGG CGC TGT GTG GAC CGT GAC TTC TGC GCC AAC ATC

F E G W R C V D R D F C A N I

CTC AGC GCC GAG AGC AGC GAC TCC GAG GGG TTT GTG ATC CAC GAC

L S A E S S D S E G F V I H D

GGC GAG TGC ATG CAG GAG TGC CCC TCG GGC TTC ATC CGC AAC GGC

G E C M Q E C P S G F I R N G

AGC CAG AGC ATG TAC TGC ATC CCT TGT GAA GGT CCT TGC CCG AAG

S Q S M Y C I P C E G P C P K

GTC TGT GAG GAA GAA AAG AAA ACA AAG ACC ATT GAT TCT GTT ACT

V C E E E K K T K T I D S V T

TCT GCT CAG ATG CTC CAA GGA TGC ACC ATC TTC AAG GGC AAT TTG

S A Q M L Q G C T I F K G N L

CTC ATT AAC ATC CGA CGG GGG AAT AAC ATT GCT TCA GAG CTG GAG

L I N I R R G N N I A S E L E

AAC TTC ATG GGG CTC ATC GAG GTG GTG ACG GGC TAC GTG AAG ATC

N F M G L I E V V T G Y V K I

CGC CAT TCT CAT GCC TTG GTC TCC TTG TCC TTC CTA AAA AAC CTT

R H S H A L V S L S F L K N L

CGC CTC ATC CTA GGA GAG GAG CAG CTA GAA GGG AAT TAC TCC TTC

R L I L G E E Q L E G N Y S F

TAC GTC CTC GAC AAC CAG AAC TTG CAG CAA CTG TGG GAC TGG GAC

Y V L D N Q N L Q Q L W D W D

CAC CGC AAC CTG ACC ATC AAA GCA GGG AAA ATG TAC TTT GCT TTC

H R N L T I K A G K M Y F A F

AAT CCC AAA TTA TGT GTT TCC GAA ATT TAC CGC ATG GAG GAA GTG

N P K L C V S E I Y R M E E V

ACG GGG ACT AAA GGG CGC CAA AGC AAA GGG GAC ATA AAC ACC AGG

T G T K G R Q S K G D I N T R

AAC AAC GGG GAG AGA GCC TCC TGT GAG AGT GAC GTC CTG CAT TTC

N N G E R A S C E S D V L H F

ACC TCC ACC ACC ACG TCG AAG AAT CGC ATC ATC ATA ACC TGG CAC

T S T T T S K N R I I I T W H

CGG TAC CGG CCC CCT GAC TAC AGG GAT CTC ATC AGC TTC ACC GTT

R Y R P P D Y R D L I S F T V

TAC TAC AAG GAA GCA CCC TTT AAG AAT GTC ACA GAG TAT GAT GGG

Y Y K E A P F K N V T E Y D G

CAG GAT GCC TGC GGC TCC AAC AGC TGG AAC ATG GTG GAC GTG GAC

Q D A C G S N S W N M V D V D

CTC CCG CCC AAC AAG GAC GTG GAG CCC GGC ATC TTA CTA CAT GGG

L P P N K D V E P G I L L H G

CTG AAG CCC TGG ACT CAG TAC GCC GTT TAC GTC AAG GCT GTG ACC

L K P W T Q Y A V Y V K A V T

CTC ACC ATG GTG GAG AAC GAC CAT ATC CGT GGG GCC AAG AGT GAG

L T M V E N D H I R G A K S E

ATC TTG TAC ATT CGC ACC AAT GCT TCA GTT CCT TCC ATT CCC TTG

I L Y I R T N A S V P S I P L

GAC GTT CTT TCA GCA TCG AAC TCC TCT TCT CAG TTA ATC GTG AAG

D V L S A S N S S S Q L I V K

TGG AAC CCT CCC TCT CTG CCC AAC GGC AAC CTG AGT TAC TAC ATT

W N P P S L P N G N L S Y Y I

GTG CGC TGG CAG CGG CAG CCT CAG GAC GGC TAC CTT TAC CGG CAC

V R W Q R Q P Q D G Y L Y R H

AAT TAC TGC TCC AAA GAC AAA ATC CCC ATC AGG AAG TAT GCC GAC

N Y C S K D K I P I R K Y A D

GGC ACC ATC GAC ATT GAG GAG GTC ACA GAG AAC CCC AAG ACT GAG

G T I D I E E V T E N P K T E

GTG TGT GGT GGG GAG AAA GGG CCT TGC TGC GCC TGC CCC AAA ACT

V C G G E K G P C C A C P K T

GAA GCC GAG AAG CAG GCC GAG AAG GAG GAG GCT GAA TAC CGC AAA

E A E K Q A E K E E A E Y R K

GTC TTT GAG AAT TTC CTG CAC AAC TCC ATC TTC GTG CCC AGG TAC

V F E N F L H N S I F V P R Y

CCA GCT CAT GTG AAA TTT CAG TTG GCA AAA CCC ACT GCT CAG GCC

P A H V K F Q L A K P T A Q A

GGT TCT GTT GCC TTT CTC CCC ACC AGG tag

G S V A F L P T R *

>EU826613,005A11,splice variant of DDR1,DDR1-286

ATG GGA CCA GAG GCC CTG TCA TCT TTA CTG CTG CTG CTC TTG GTG

M G P E A L S S L L L L L L V

GCA AGT GGA GAT GCT GAC ATG AAG GGA CAT TTT GAT CCT GCC AAG

A S G D A D M K G H F D P A K

TGC CGC TAT GCC CTG GGC ATG CAG GAC CGG ACC ATC CCA GAC AGT

C R Y A L G M Q D R T I P D S

GAC ATC TCT GCT TCC AGC TCC TGG TCA GAT TCC ACT GCC GCC CGC

D I S A S S S W S D S T A A R

CAC AGC AGG TTG GAG AGC AGT GAC GGG GAT GGG GCC TGG TGC CCC

H S R L E S S D G D G A W C P

GCA GGG TCG GTG TTT CCC AAG GAG GAG GAG TAC TTG CAG GTG GAT

A G S V F P K E E E Y L Q V D

CTA CAA CGA CTG CAC CTG GTG GCT CTG GTG GGC ACC CAG GGA CGG

L Q R L H L V A L V G T Q G R

CAT GCC GGG GGC CTG GGC AAG GAG TTC TCC CGG AGC TAC CGG CTG

H A G G L G K E F S R S Y R L

CGT TAC TCC CGG GAT GGT CGC CGC TGG ATG GGC TGG AAG GAC CGC

R Y S R D G R R W M G W K D R

TGG GGT CAG GAG GTG ATC TCA GGC AAT GAG GAC CCT GAG GGA GTG

W G Q E V I S G N E D P E G V

GTG CTG AAG GAC CTT GGG CCC CCC ATG GTT GCC CGA CTG GTT CGC

V L K D L G P P M V A R L V R

TTC TAC CCC CGG GCT GAC CGG GTC ATG AGC GTC TGT CTG CGG GTA

F Y P R A D R V M S V C L R V

GAG CTC TAT GGC TGC CTC TGG AGG GAT GGA CTC CTG TCT TAC ACC

E L Y G C L W R D G L L S Y T

GCC CCT GTG GGG CAG ACA ATG TAT TTA TCT GAG GCC GTG TAC CTC

A P V G Q T M Y L S E A V Y L

AAC GAC TCC ACC TAT GAC GGA CAT ACC GTG GGC GGA CTG CAG TAT

N D S T Y D G H T V G G L Q Y

GGG GGT CTG GGC CAG CTG GCA GAT GGT GTG GTG GGG CTG GAT GAC

G G L G Q L A D G V V G L D D

TTT AGG AAG AGT CAG GAG CTG CGG GTC TGG CCA GGC TAT GAC TAT

F R K S Q E L R V W P G Y D Y

GTG GGA TGG AGC AAC CAC AGC TTC TCC AGT GGC TAT GTG GAG ATG

V G W S N H S F S S G Y V E M

GAG TTT GAG TTT GAC CGG CTG AGG GCC TTC CAG GCT ATG CAG ATG

E F E F D R L R A F Q A M Q M

TGG TGA

W *

>EU826614,005A10,splice variant of DDR1,DDR1-243

ATG GGA CCA GAG GCC CTG TCA TCT TTA CTG CTG CTG CTC TTG GTG

M G P E A L S S L L L L L L V

GCA AGT GGA GAT GCT GAC ATG AAG GGA CAT TTT GAT CCT GCC AAG

A S G D A D M K G H F D P A K

TGC CGC TAT GCC CTG GGC ATG CAG GAC CGG ACC ATC CCA GAC AGT

C R Y A L G M Q D R T I P D S

GAC ATC TCT GCT TCC AGC TCC TGG TCA GAT TCC ACT GCC GCC CGC

D I S A S S S W S D S T A A R

CAC AGC AGG TTG GAG AGC AGT GAC GGG GAT GGG GCC TGG TGC CCC

H S R L E S S D G D G A W C P

GCA GGG TCG GTG TTT CCC AAG GAG GAG GAG TAC TTG CAG GTG GAT

A G S V F P K E E E Y L Q V D

CTA CAA CGA CTG CAC CTG GTG GCT CTG GTG GGC ACC CAG GGA CGG

L Q R L H L V A L V G T Q G R

CAT GCC GGG GGC CTG GGC AAG GAG TTC TCC CGG AGC TAC CGG CTG

H A G G L G K E F S R S Y R L

CGT TAC TCC CGG GAT GGT CGC CGC TGG ATG GGC TGG AAG GAC CGC

R Y S R D G R R W M G W K D R

TGG GGT CAG GAG GTG ATC TCA GGC AAT GAG GAC CCT GAG GGA GTG

W G Q E V I S G N E D P E G V

GTG CTG AAG GAC CTT GGG CCC CCC ATG GTT GCC CGA CTG GTT CGC

V L K D L G P P M V A R L V R

TTC TAC CCC CGG GCT GAC CGG GTC ATG AGC GTC TGT CTG CGG GTA

F Y P R A D R V M S V C L R V

GAG CTC TAT GGC TGC CTC TGG AGG GAC TGC AGT ATG GGG GTC TGG

E L Y G C L W R D C S M G V W

GCC AGC TGG CAG ATG GTG TGG TGG GGC TGG ATG ACT TTA GGA AGA

A S W Q M V W W G W M T L G R

GTC AGG AGC TGC GGG TCT GGC CAG GCT ATG ACT ATG TGG GAT GGA

V R S C G S G Q A M T M W D G

GCA ACC ACA GCT TCT CCA GTG GCT ATG TGG AGA TGG AGT TTG AGT

A T T A S P V A M W R W S L S

TTG ACC GGC TGA

L T G *

>EU826615,003H02,splice variant of TNFR1B,TNFR1B-155

ATG GCG CCC GTC GCC GTC TGG GCC GCG CTG GCC GTC GGA CTG GAG

M A P V A V W A A L A V G L E

CTC TGG GCT GCG GCG CAC GCC TTG CCC GCC CAG GTG GCA TTT ACA

L W A A A H A L P A Q V A F T

CCC TAC GCC CCG GAG CCC GGG AGC ACA TGC CGG CTC AGA GAA TAC

P Y A P E P G S T C R L R E Y

TAT GAC CAG ACA GCT CAG ATG TGC TGC AGC AAA TGC TCG CCG GGC

Y D Q T A Q M C C S K C S P G

CAA CAT GCA AAA GTC TTC TGT ACC AAG ACC TCG GAC ACC GTG TGT

Q H A K V F C T K T S D T V C

GAC TCC TGT GAG GAC AGC ACA TAC ACC CAG CTC TGG AAC TGG GTT

D S C E D S T Y T Q L W N W V

CCC GAG TGC TTG AGC TGT GGC TCC CGC TGT AGC TCT GAC CAG GTG

P E C L S C G S R C S S D Q V

GAA ACT CAA GCC TGC ACT CGG GAA CAG AAC CGC ATC TGC ACC TGC

E T Q A C T R E Q N R I C T C

AGG CCC GGC TGG TAC TGC GCG CTG AGC AAG CAG GAG GGG TGC CGG

R P G W Y C A L S K Q E G C R

CTG TGC GCG CCG CTG CGC AAG TGC CGC CCG GGC TTC GGC GTG GCC

L C A P L R K C R P G F G V A

AGA CCA GAC CTC TCC TAG

R P D L S *

>EU826616,021A05,splice variant of RAGE,RAGE-146

ATG GCA GCC GGA ACA GCA GTT GGA GCC TGG GTG CTG GTC CTC AGT

M A A G T A V G A W V L V L S

CTG TGG GGG GCA GTA GTA GGT GCT CAA AAC ATC ACA GCC CGG ATT

L W G A V V G A Q N I T A R I

GGC GAG CCA CTG GTG CTG AAG TGT AAG GGG GCC CCC AAG AAA CCA

G E P L V L K C K G A P K K P

CCC CAG CGG CTG GAA TGG AAA CTG AAC ACA GGC CGG ACA GAA GCT

P Q R L E W K L N T G R T E A

TGG AAG GTC CTG TCT CCC CAG GGA GGA GGC CCC TGG GAC AGT GTG

W K V L S P Q G G G P W D S V

GCT CGT GTC CTT CCC AAC GGC TCC CTC TTC CTT CCG GCT GTC GGG

A R V L P N G S L F L P A V G

ATC CAG GAT GAG GGG ATT TTC CGG TGC CAG GCA ATG AAC AGG AAT

I Q D E G I F R C Q A M N R N

GGA AAG GAG ACC AAG TCC AAC TAC CGA GTC CGT GTC TAC CGT AAG

G K E T K S N Y R V R V Y R K

AAT TCC AGG GTC TTC TCC AAG GCC TCC CTC TTA CCT AAG AAA AAG

N S R V F S K A S L L P K K K

CCT TCA ACC CCA GCC TTG GCC CAT GAG GGC CTC TGA

P S T P A L A H E G L *

>EU826617,021F06,splice variant of RAGE,RAGE-172

ATG GCA GCC GGA ACA GCA GTT GGA GCC TGG GTG CTG GTC CTC AGT

M A A G T A V G A W V L V L S

CTG TGG GGG GCA GTA GTA GGT GCT CAA AAC ATC ACA GCC CGG ATT

L W G A V V G A Q N I T A R I

GGC GAG CCA CTG GTG CTG AAG TGT AAG GGG GCC CCC AAG AAA CCA

G E P L V L K C K G A P K K P

CCC CAG CGG CTG GAA TGG AAA CTG AAC ACA GGC CGG ACA GAA GCT

P Q R L E W K L N T G R T E A

TGG AAG GTC CTG TCT CCC CAG GGA GGA GGC CCC TGG GAC AGT GTG

W K V L S P Q G G G P W D S V

GCT CGT GTC CTT CCC AAC GGC TCC CTC TTC CTT CCG GCT GTC GGG

A R V L P N G S L F L P A V G

ATC CAG GAT GAG GGG ATT TTC CGG TGC CAG GCA ATG AAC AGG AAT

I Q D E G I F R C Q A M N R N

GGA AAG GAG ACC AAG TCC AAC TAC CGA GTC CGT GTC TAC CAG ATT

G K E T K S N Y R V R V Y Q I

CCT GGG AAG CCA GAA ATT GTA GAT TCT GCC TCT GAA CTC ACG GCT

P G K P E I V D S A S E L T A

GGT GTT CCC AAT AAG GTG GGG ACA TGT GTG TCA GAG GGA AGC TAC

G V P N K V G T C V S E G S Y

CCT GCA GGG ACT CTT AGC TGG CAC TTG GAT GGG AAG CCC CTG GTG

P A G T L S W H L D G K P L V

CCT AAT GAG AAG GGT GAG TCC TAA

P N E K G E S *

>EU826618,021C06,splice variant of RAGE,RAGE-387

ATG GCA GCC GGA ACA GCA GTT GGA GCC TGG GTG CTG GTC CTC AGT

M A A G T A V G A W V L V L S

CTG TGG GGG GCA GTA GTA GGT GCT CAA AAC ATC ACA GCC CGG ATT

L W G A V V G A Q N I T A R I

GGC GAG CCA CTG GTG CTG AAG TGT AAG GGG GCC CCC AAG AAA CCA

G E P L V L K C K G A P K K P

CCC CAG CGG CTG GAA TGG AAA CTG AAC ACA GGC CGG ACA GAA GCT

P Q R L E W K L N T G R T E A

TGG AAG GTC CTG TCT CCC CAG GGA GGA GGC CCC TGG GAC AGT GTG

W K V L S P Q G G G P W D S V

GCT CGT GTC CTT CCC AAC GGC TCC CTC TTC CTT CCG GCT GTC GGG

A R V L P N G S L F L P A V G

ATC CAG GAT GAG GGG ATT TTC CGG TGC CAG GCA ATG AAC AGG AAT

I Q D E G I F R C Q A M N R N

GGA AAG GAG ACC AAG TCC AAC TAC CGA GTC CGT GTC TAC CAG ATT

G K E T K S N Y R V R V Y Q I

CCT GGG AAG CCA GAA ATT GTA GAT TCT GCC TCT GAA CTC ACG GCT

P G K P E I V D S A S E L T A

GGT GTT CCC AAT AAG GTG GGG ACA TGT GTG TCG GAG GGA AGC TAC

G V P N K V G T C V S E G S Y

CCT GCA GGG ACT CTT AGC TGG CAC TTG GAT GGG AAG CCC CTG GTG

P A G T L S W H L D G K P L V

CCT AAT GAG AAG GGA GTA TCT GTG AAG GAA CAG ACC AGG AGA CAC

P N E K G V S V K E Q T R R H

CCT GAG ACA GGG CTC TTC ACA CTG CAG TCG GAG CTA ATG GTG ACC

P E T G L F T L Q S E L M V T

CCA GCC CGG GGA GGA GAT CCC CGT CCC ACC TTC TCC TGT AGC TTC

P A R G G D P R P T F S C S F

AGC CCA GGC CTT CCC CGA CAC CGG GCC TTG CGC ACA GCC CCC ATC

S P G L P R H R A L R T A P I

CAG CCC CGT GTC TGG GAG CCT GTG CCT CTG GAG GAG GTC CAA TTG

Q P R V W E P V P L E E V Q L

GTG GTG GAG CCA GAA GGT GGA GCA GTA GCT CCT GGT GGA ACC GTA

V V E P E G G A V A P G G T V

ACC CTG ACC TGT GAA GTC CCT GCC CAG CCC TCT CCT CAA ATC CAC

T L T C E V P A Q P S P Q I H

TGG ATG AAG GAT GGG GTG CCC TTG CCC CTT CCC CCC AGC CCT GTG

W M K D G V P L P L P P S P V

CTG ATC CTC CCT GAG ATA GGG CCT CAG GAC CAG GGA ACC TAC AGC

L I L P E I G P Q D Q G T Y S

TGT GTG GCC ACC CAT TCC AGC CAC GGG CCC CAG GAA AGC CGT GCT

C V A T H S S H G P Q E S R A

GTC AGC ATC AGC ATC ATC GGT GAG ACC TCT CCC CAA GCC CTA CAG

V S I S I I G E T S P Q A L Q

ACC CTG GGA CTA GGG TGC AGG ACA GCA CAG GCT CTA ATT TCC TGC

T L G L G C R T A Q A L I S C

CCC ATT CTG GCC TTA TCC CTA ACA GCC ACC CCA CCT CTC CCT CCA

P I L A L S L T A T P P L P P

TGC ACC CAC ACC CAA GCC TCC CCT GCC CCA CCC AAA TTC TGC CAA

C T H T Q A S P A P P K F C Q

GAG AGC AGC CAA GCC TCT CCC TTC TTC CCT CTG AGC TAA

E S S Q A S P F F P L S *

>EU826619,021C02,splice variant of RAGE,RAGE-266

ATG GCA GCC GGA ACA GCA GTT GGA GCC TGG GTG CTG GTC CTC AGT

M A A G T A V G A W V L V L S

CTG TGG GGG GCA GTA GTA GGT GCT CAA AAC ATC ACA GCC CGG ATT

L W G A V V G A Q N I T A R I

GGC GAG CCA CTG GTG CTG AAG TGT AAG GGG GCC CCC AAG AAA CCA

G E P L V L K C K G A P K K P

CCC CAG CGG CTG GAA TGG AAA CTG AAC ACA GGC CGG ACA GAA GCT

P Q R L E W K L N T G R T E A

TGG AAG GTC CTG TCT CCC CAG GGA GGA GGC CCC TGG GAC AGT GTG

W K V L S P Q G G G P W D S V

GCT CGT GTC CTT CCC AAC GGC TCC CTC TTC CTT CCG GCT GTC GGG

A R V L P N G S L F L P A V G

ATC CAG GAT GAG GGG ATT TTC CGG TGC CAG GCA ATG AAC AGG AAT

I Q D E G I F R C Q A M N R N

GGA AAG GAG ACC AAG TCC AAC TAC CGA GTC CGT GTC TAC CAG ATT

G K E T K S N Y R V R V Y Q I

CCT GGG AAG CCA GAA ATT GTA GAT TCT GCC TCT GAA CTC ACG GCT

P G K P E I V D S A S E L T A

GGT GTT CCC AAT AAG GTA GTG GAA GAA AGC AGG AGA AGT AGA AAA

G V P N K V V E E S R R S R K

CGG CCC TGT GAA CAG GAG GTG GGG ACA TGT GTG TCA GAG GGA AGC

R P C E Q E V G T C V S E G S

TAC CCT GCA GGG ACT CTT AGC TGG CAC TTG GAT GGG AAG CCC CTG

Y P A G T L S W H L D G K P L

GTG CCT AAT GAG AAG GGA GTA TCT GTG AAG GAA CAG ACC AGG AGA

V P N E K G V S V K E Q T R R

CAC CCT GAG ACA GGG CTC TTC ACA CTG CAG TCG GAG CTA ATG GTG

H P E T G L F T L Q S E L M V

ACC CCA GCC CGG GGA GGA GAT CCC CGT CCC ACC TTC TCC TGT AGC

T P A R G G D P R P T F S C S

TTC AGC CCA GGC CTT CCC CGA CAC CGG GCC TTG CGC ACA GCC CCC

F S P G L P R H R A L R T A P

ATC CAG CCC CGT GTC TGG GGT GAG CAT AGG TGG GGA GGG CCC CAA

I Q P R V W G E H R W G G P Q

GCT CAC GTG AGC ACG TTC TGG AAG TCT GAC CCT TAG

A H V S T F W K S D P *

>EU826620,021A11,splice variant of RAGE,RAGE-128

ATG GCA GCC GGA ACA GCA GTT GGA GCC TGG GTG CTG GTC CTC AGT

M A A G T A V G A W V L V L S

CTG TGG GGG GCA GTA GTA GGT GCT CAA AAC ATC ACA GCC CGG ATT

L W G A V V G A Q N I T A R I

GGC GAG CCA CTG GTG CTG AAG TGT AAG GGG GCC CCC AAG AAA CCA

G E P L V L K C K G A P K K P

CCC CAG CGG CTG GAA TGG AAA CTG AAC ACA GGC CGG ACA GAA GCT

P Q R L E W K L N T G R T E A

TGG AAG GTC CTG TCT CCC CAG GGA GGA GGC CCC TGG GAC AGT GTG

W K V L S P Q G G G P W D S V

GCT CGT GTC CTT CCC AAC GGC TCC CTC TTC CTT CCG GCT GTC GGG

A R V L P N G S L F L P A V G

ATC CAG GAT GAG GGG ATT TTC CGG TGC CAG GCA ATG AAC AGG AAT

I Q D E G I F R C Q A M N R N

GGA AAG GAG ACC AAG TCC AAC TAC CGA GTC CGT GTC TAC CAG TTT

G K E T K S N Y R V R V Y Q F

GAG AAC CTT CAC AAT TAC AGC CTC TGA

E N L H N Y S L *
